# Large-scale detection of drug off-targets: hypotheses for drug repurposing and understanding side-effects

**DOI:** 10.1186/s40360-017-0128-7

**Published:** 2017-04-28

**Authors:** Matthieu Chartier, Louis-Philippe Morency, María Inés Zylber, Rafael J. Najmanovich

**Affiliations:** 1 Department of Biochemistry, Faculty of Medicine and Health Sciences, Université de Sherbrooke, Québec, Canada; 2 Department of Pharmacology and Physiology, Faculty of Medicine, Université de Montréal, Québec, Canada

**Keywords:** Molecularinteraction field similarities, Binding-site similarities, Drug repurposing, Side-effects, Cross-reactivity, Promiscuity, Large-scale analysis

## Abstract

**Background:**

Promiscuity in molecular interactions between small-molecules, including drugs, and proteins is widespread. Such unintended interactions can be exploited to suggest drug repurposing possibilities as well as to identify potential molecular mechanisms responsible for observed side-effects.

**Methods:**

We perform a large-scale analysis to detect binding-site molecular interaction field similarities between the binding-sites of the primary target of 400 drugs against a dataset of 14082 cavities within 7895 different proteins representing a non-redundant dataset of all proteins with known structure. Statistically-significant cases with high levels of similarities represent potential cases where the drugs that bind the original target may in principle bind the suggested off-target. Such cases are further analysed with docking simulations to verify if indeed the drug could, in principle, bind the off-target. Diverse sources of data are integrated to associated potential cross-reactivity targets with side-effects.

**Results:**

We observe that promiscuous binding-sites tend to display higher levels of hydrophobic and aromatic similarities. Focusing on the most statistically significant similarities (Z-score ≥ 3.0) and corroborating docking results (RMSD < 2.0 Å), we find 2923 cases involving 140 unique drugs and 1216 unique potential cross-reactivity protein targets. We highlight a few cases with a potential for drug repurposing (acetazolamide as a chorismate pyruvate lyase inhibitor, raloxifene as a bacterial quorum sensing inhibitor) as well as to explain the side-effects of zanamivir and captopril. A web-interface permits to explore the detected similarities for each of the 400 binding-sites of the primary drug targets and visualise them for the most statistically significant cases.

**Conclusions:**

The detection of molecular interaction field similarities provide the opportunity to suggest drug repurposing opportunities as well as to identify potential molecular mechanisms responsible for side-effects. All methods utilized are freely available and can be readily applied to new query binding-sites. All data is freely available and represents an invaluable source to identify further candidates for repurposing and suggest potential mechanisms responsible for side-effects.

**Electronic supplementary material:**

The online version of this article (doi:10.1186/s40360-017-0128-7) contains supplementary material, which is available to authorized users.

## Background

Molecular promiscuity can be described as the situation in which small molecules and proteins participate in molecular interactions beyond those naturally selected or, in the case of drugs, designed. Small molecules, even FDA-approved drugs, are often more promiscuous than initially anticipated as a result of the complexity of cellular environments. Experimental assays of 72 inhibitors with 442 kinases showed that 64% of the compounds bind 20% of kinases with an affinity threshold of 3 μM [1]. Among these 72 inhibitors, 11 are FDA-approved drugs. Another inter-family large-scale study with data for 238 655 compounds and 2876 targets, showed that promiscuity is often within the same protein family, but also among members of different protein families [2]. Promiscuity can play a role in the appearance of side-effects, but could also be leveraged in polypharmacological strategies or repurposing.

Promiscuity is often perceived negatively because of side-effects that can occur when the drug modulates the activity of off-targets. Toxicity issues are responsible for nearly 30% of failures in drug development programs [3]. The side-effects associated with common off-targets are well documented and these targets are often screened during drug development to decrease the risks of side-effects during subsequent development phases [4].

The predominant dogma was often one disease, one target, one drug, where the drug had to be as selective as possible. The increasing comprehension of metabolic networks and their properties [5], like the redundancy of signaling pathways, can influence the targeting strategy. Indeed, the modulation of multiple key targets in the network by a single therapeutic drug, a strategy termed polypharmacology, could be more efficient than the one-drug one-target approach [6]. The promiscuous nature of certain drugs could therefore be leveraged for these multi-target tactics targeting the same condition or a different one.

Large-scale analyses of promiscuity can lead to interesting discoveries and novel treatment avenues. For example, an approved drug capable of modulating the activity of an off-target could suggest a repurposing of this drug. This is particularly interesting if the compound is an FDA-approved drug as it could be brought to market more rapidly and economically.

In order to exploit promiscuity, its underlying factors must be understood more clearly. The ability of a ligand to bind multiple targets likely depends on ligand-based and target-based properties, as both are inter-dependent. Ligand hydrophobicity is generally correlated with promiscuity [7]. Haupt et al. also found a correlation between binding promiscuity and ligand flexibility [8]. In the latter study, target binding-site similarity was also shown to correlate with promiscuity, at least for the PDB structure dataset used. Environmental conditions, post-translational modifications and target structural plasticity are factors known to play a role in promiscuity not only for protein-ligand, but also for protein-protein interactions [9]. Ultimately, in order to understand promiscuity, the cellular contexts of such interactions must be taken in consideration [10].

The importance of both ligand and protein binding-site features is reflected on the existence of ligand-based and target-based methods to predict off-targets. Paolini et al. built Bayesian classification models for 698 targets using the structures of their ligands, obtaining a 153-fold enrichment compared to random in the prediction of targets for such drugs [2]. Another ligand-based method, the Similarity Ensemble Approach (SEA) [11] compares a ligand to ligand ensembles. The SEA method was employed on 3665 approved or investigational drugs against 246 targets. Some predictions made were validated experimentally [12]. The method was also used to find targets related to observed side-effects for 656 drugs [13].

There are a number of target-based methods for the detection of binding-site similarities [14]. Among these, SOIPPA [15], CavBase [16], eMatchSite [17], IsoCleft [10, 18] and IsoMIF [19, 20]. Such methods can be used to predict protein function from structure [21–23], understand promiscuity within a protein family [24, 25], assess drugability [26], and explain the cross-reactivity of drugs. The ability of CavBase to predict off-targets for 16 kinase inhibitors was evaluated with ROC (Receiving Operating Characteristic) curves giving an average AUC (Area Under the Curve) of 0.70. SOIPPA predicted off-targets of selective estrogen receptor modulators [27] and of torcetrapib [28] in order to explain side-effect mechanisms. There are also inverse docking methods, such as Target Fishing Dock [29], where a ligand is screened against a panel of target structures and the ones with the best scores are retained.

In the current work we perform a large-scale analysis of binding-sites of targets for an ensemble of drugs using IsoMIF, a method that detects molecular interaction field (MIF) similarities between binding-sites. IsoMIF was shown to outperform existing methods on a variety of datasets providing a higher and more robust measure of average AUC values across datasets [19]. The binding-sites of drug targets are compared to cavities in a non-redundant subset of proteins with known structures. The resulting predictions are used to generate hypotheses of two types. First, the new targets predicted could represent drug repurposing avenues and, second, they could be used to explain known side-effects of the drugs. For the most significant predictions, molecular docking simulations were performed using the FlexAID algorithm [30] to determine the potential docking pose of the drug in the potential cross-reactivity target. Poses of the ligand obtained by superimposing the drug bound target on the predicted cross-reactivity target using MIF similarities were compared to the pose obtained by the docking algorithm allowing to rationalize the prediction by looking at potential interactions in the target binding-site.

We provide specific examples of hypotheses regarding repurposing and side-effect mechanisms. Furthermore, all the data of the analyses is made available through a web interface including PyMOL sessions representing the detected MIF similarities and docking poses. Lists of interesting cases, i.e. those with high levels of MIF similarities and small RMSD between the IsoMIF and the FlexAID docking poses are made available through the interface at bcb.med.usherbrooke.ca/drugs.php.

## Methods

### Definition of binding-sites

#### Drug dataset targets

The list of ligands was obtained from the Drug and Drug Target Mapping, an RCSB resource [31] available on the Protein Data Bank web site [32]. This list contains all the PDB structures crystalized with a ligand mapped in Drugbank [33, 34]. The structure does not always represent the primary target of the drug and sometimes one drug can have multiple targets, but a structure is retained only if it has at least 30% sequence identity with one of the known primary targets of the respective drug. The list is filtered to remove structures containing RNA or DNA structures. Each entry is named with the following nomenclature 2ITY_IRE_2020_A_-, where 2ITY is the 4 letter PDB code and IRE 2020 A – the PDB ligand code, number, chain and alternate location (‘-’ if none) respectively. The dataset is available for download from our site. For simplicity, we refer to this dataset as Drugs dataset.

#### Non-redundant protein structure dataset

The binding-site of every drug was compared against a dataset of potential target binding-sites obtained from the PISCES server [35]. This dataset represents the largest non-redundant set of structures currently known from a sequence point-of-view according to PSI-BLAST with a 30% sequence identity threshold. The structures also respect certain quality criteria. They have at least a 2.0 Å resolution and an R-factor of 2.0. The list contains 8016 PDB chains. The dataset is available for download from our site. For simplicity, we refer to this dataset as Pisces dataset.

#### Detection of binding-site similarities and docking simulations

The similarities between each drug binding-site and each Pisces binding-site were detected using IsoMIF and its default parameters, and a grid with 1.5 Å spacing. Cavities were identified using the GetCleft algorithm [36]. IsoMIF offers several advantages over existing methods for the detection of similarities, particularly across protein families and between binding-sites that share little or no evolutionary relationships. One notable advantage of using IsoMIF is that it is agnostic to the nature of amino acids lining the cavities under comparison but instead it relies on the detection of similarities in the interactions that are deemed favourable in particular positions in the two cavities. In the Drugs dataset, the MIFs were defined using a 3 Å threshold around ligands. For the structures in Pisces, the top 2 largest cavities found using GetCleft in contact with the Pisces PDB chain and in contact with at most 250 residues were retained. Another advantage of the IsoMIF method is that it can handle such large cavities and still find the largest sub-volume of MIF similarities.

For each target, two measures of binding-site similarity are calculated by IsoMIF, the Tanimoto coefficient and the fraction of significant *MIF* *P* robes in *C*ommon of the *q*uery (MPC_q_). The Tanimoto coefficient is the same as described in [19] and MPC_q_ represents the fraction of the significant MIF probes (as defined in [19]) identified in the Drugs binding-site found similar to the MIF of the Pisces binding-site. As opposed to the Tanimoto, MPC_q_ is not affected by the initial volume of the Pisces cavity, an uncontrolled parameter in this study.

For each drug, the top targets were sorted using the Z-score calculated for the Tanimoto coefficient and the MPC_q_ measure for each Drug-Pisces binding-site combination. Whenever a Drug binding-site and a Pisces cavity have a Tanimoto coefficient of similarity or MPC_q_ with Z-score ≥ 3.0 (further referred as Z_3_), the two were superimposed using the transformation matrix that best superimposes the detected MIF similarities. This allows us to obtain a rough pose of the ligand in the Pisces cavity.

Docking simulations for targets with Z_3_ were performed using FlexAID [30]. FlexAID is a probabilistic genetic-algorithm based method. To ensure a satisfactory coverage of the search space, each simulation was repeated 10 times with a population size of 1000 chromosomes and for 1000 generations for a total of 10^6^ energy evaluations. The RMSD between the top 25 poses for each Drug-Pisces combination and the pose obtained after the superimposition with the similarities detected by IsoMIF were calculated. For each Drug-Pisces combination the pose with the best RMSD and with the best docking score was retained. Docked ligands with a small RMSD with respect to the pose superimposed using the binding-site similarities represent independent corroborating evidence that the ligand could bind the cross-reactivity binding-site. Specifically, it indicates that those groups responsible for the conserved molecular interaction field similarities are also responsible for favourable interactions with the ligand.

#### Drug side-effects and target data

For each drug entry, the toxicity information was obtained from Drugbank when available. Also, using the PubChem identifier of the drug, side-effects from Sider [37] were fetched with the observed frequency when available. For each Pisces protein, cross-referenced information was retrieved from the Uniprot database [38]. We also provide links to Pubmed articles related to the target, the associated diseases found on Disgenet [39], rare diseases on Orphanet [40], metabolic pathways from Reactome [41, 42] and gene ontology information, namely cellular function, localisation and biological processes [43]. A list of target-related keywords was also retrieved from Uniprot.

#### Web-interface

A web interface was built to make the sorted target list available for each drug entry. For each entry, the Drugbank toxicity and Sider side-effects with their frequency is given. Side effects are sorted by frequency with a color-code, towards red as the frequency increases. The sequence identity between the drug-bound protein and the primary target(s) of the drug is displayed.

For each drug entry, the Pisces targets with Z-score ≥ 2.0 (Z_2_) are shown by default, although this threshold can be defined by the user. Also, the list can be filtered to show only human structures. The cross-referenced information is given for each Pisces target when available. A yellow exclamation mark icon tags the reference list of the target when the title contains one of the following keywords: inhibit, agonist, target, drug, resistance, treatment, therapy, cancer, disease, ligand, pathogen, toxic, side effect, adverse effect. The keywords are (put) in bold in the title of the reference for easy identification.

For each target in the Z_3_ category, a PyMOL session showing the similarities detected by IsoMIF can be downloaded and a PNG image can be seen showing the color-coded similarities detected. Furthermore, for Z_3_ category targets, the results of the docking simulations are shown and a PyMOL session can be downloaded, similar to the one with the MIF similarities, but with the docking pose in the target binding-site.

## Results

### Statistics of the datasets

The Drugs dataset contained 400 binding-sites for which the structures had an average sequence identity of 69% ± 25% to the primary targets. These 400 entries represent binding-sites of 186 unique drugs. Redundant drugs include acetazolamide (14 entries), tretinoin (7 entries) as well as zanamivir, progesterone, sirolimus, liothyronine, vorinostat, estradiol and tetrahydrobiopterine represented in 6 entries each (Additional file 1: Table S1).

The final Pisces dataset contains 14082 cavities with 39 residues on average (Additional file 1: Figure S1). These 14082 cavities represent 7633 different PDB entries, 3539 Pfam families, 7895 Uniprot entries, and 1445 different organisms with a total of 2007 binding-sites from *Homo sapiens* proteins.

Drugbank toxicity information was available for 262 of the 400 drug entries and Sider side effects for 241 of the 400 entries. There was on average 163 side effects per Sider entry. Additional file 1: Table S1 shows the list of unique ligands with the number of representative binding-sites in the Drugs dataset, and the number of side recorded side effects.

### Binding-site similarity and docking simulations

More than 5,632,800 binding-site comparisons were performed using IsoMIF. For all the Drugs binding-sites, the number of targets predicted with Z_2_ and Z_3_ were 168,906 and 9845, respectively. A total of 9845 docking simulations were performed (for each Drug/Pisces combination with Z_3_) among which 4764 (48.4%) had a top pose with an RMSD of at most 3.0 Å. This number decreases to 2923 (29.6%) for an RMSD threshold of 2.0 Å. In such cases the binding-site MIF similarities likely represent important interactions responsible for binding in the primary target and that are conserved in the potential cross-reactivity target. The targets predicted for each drug with Z_3_ and with an RMSD of at most 3.0 Å or 2.0 Å are given in two Excel files available as supplementary data containing respectively 4764 (154 unique drugs and 1410 unique potential cross-reactivity protein targets) and 2923 (140 unique drugs and 1216 unique potential cross-reactivity protein targets, representing approximately 15% of all entries in the Pisces dataset). Additional file 1: Table S2 shows each of the 400 Drug entries sorted by number of predicted targets at Z_3_ and the number of ligand heavy atoms (i.e., non-Hydrogen atoms) of the drug, the number of Pfam families represented by the predicted targets, and the number of references with at least one special keyword in the title. Whereas we only discuss a few such targets in this work, the online repository represents a valuable source of data for further analyses and a source of hypotheses to be tested experimentally.

Potential cross-reactivity targets predicted at least twice for the same drug using different query entries are listed in Additional file 1: Table S3. For simplicity, only ligands represented in at least 4 different PDB structures are listed. The number of times the target is predicted with a Z-score higher than 3.0, 2.5 and 2.0 is given with the name of the target protein and the Drug entry ID for which the target is predicted with Z_3_. Looking at the predicted targets for the top 3 most common Drugs, namely acetazolamide, tretinoin and zanamivir, at least one of their primary targets is predicted by IsoMIF, them being carbonic anhydrase 2, retinoic acid receptor RXR-beta and neuraminidase, respectively. For 14 query binding-sites of the Drugs dataset bound to acetazolamide, carbonic anhydrase 2 is predicted 8 times with Z_3_, and 13 times with a Z_2_. For 7 query binding-sites bound to tretinoin, the retinoic acid receptor RXR-beta is predicted 3 times with Z_3_ and 7 times with Z_2_. Neuraminidase is predicted with Z_3_ for all six query binding-sites of zanamivir. Additional file 1: Table S4 shows the 554 most common binding-sites, that is, those predicted with Z_3,_ for at least 5 Drug entries. SEC14-Like protein 3, mineralocorticoid receptor, ring finger protein 4 and leukotriene C4 synthase were predicted 78, 60, 44 and 43 times, respectively, with the Z_3_ threshold. The specific z-score values for each of the drugs above and images of the detected similarities can be found in the online depository.

### Non-polar interactions are over represented in promiscuous binding-sites

**Fig. 1:**
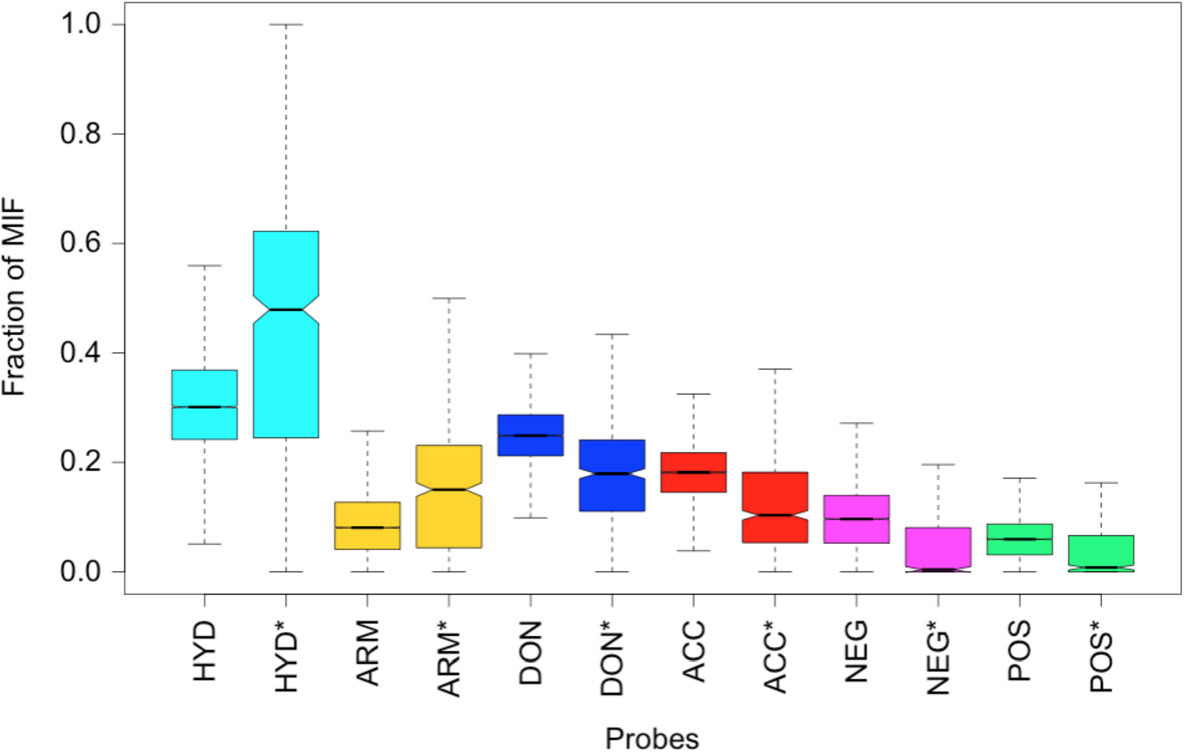
Fraction of MIFs for the common Pisces entries. Boxplots showing the fraction of the MIFs represented by the 6 probe types in the 14082 Pisces binding-sites compared to the ones measured using only the 554 most commonly predicted similar target binding-sites for each probe type (marked by *). HYD: Hydrophobic, ARM: Aromatic, DON: Hydrogen bond donor, ACC: Hydrogen bond acceptor, NEG: Negatively charged, and POS: Positively charged

Figure 1 shows the fraction of MIFs represented by each probe type for the 14082 Pisces binding-sites and for the subset of 554 among these that were most commonly found to be similar to query binding-sites. These common binding-sites have significantly more fractions of hydrophobic and aromatic probes than the average fraction in all binding-sites (parametric p-value < 2.2 × 10^−16^). Other probes are on average less represented in the subset of ‘promiscuous’ binding-sites.

### Ligand promiscuity

There are 25 targets in average predicted per Drug entry in the Z_3_ category. Among ligands that have the most predicted targets (Additional file 1: Table S2), are ethanol, acetohydroxamic acid, dichloroacetic acid and salicylic acid. These are small in terms of number of heavy atoms. Thus, it is more likely to detect binding-sites that contain atomic arrangements that satisfy the limited number of favourable interactions to bind such ligands.

**Fig. 2:**
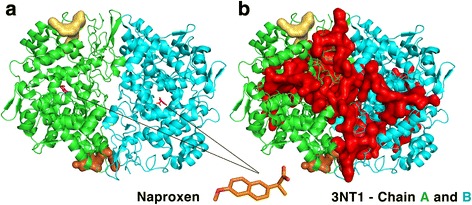
Cavities of Prostaglandin-endoperoxide synthase 2 (PDB 3NT1). **a** The two cavities 3NT1_2 (*pale yellow*) and 3NT1_3 (*orange*) define the two binding-sites of this protein in the Pisces dataset. The bound naproxen is also shown. **b** Cavity 3NT1_1, in *red*, covers the naproxen binding-site, but was excluded because of its size

The two measures of binding-site similarity used have their merits and disadvantages. For a fixed detected number of common probes between query and target search cavities, the Tanimoto coefficient is affected by differences in the volume of cavities whereas the MPC_q_ (measure) is not affected. Biologically, the two similarity measures are relevant, but MPC_q_ is used to identify cases where the Tanimoto coefficient would fail to yield a high similarity score because of the binding-site volume difference. This difference would occur especially if GetCleft identifies one large cavity composed of small interconnected cavities as shown below, but these were filtered out using the 250 residues cavity size limit. Naproxen with 17 heavy atoms is the next ligand identified in Additional file 1: Table S2 with 122 targets predicted at Z_3_. Naproxen is a nonsteroidal anti-inflammatory drug capable of binding only via aromatic and nonpolar interactions [44]. Its primary target, Prostaglandin-endoperoxide synthase 2, was identified at a low rank of 3687 with a weak Z-score of 0.55. The reason behind this low similarity is that the naproxen binding-site on the primary target was part of a large cavity that was filtered out (Fig. 2) by the 250 residues size filter. This shows that, in some cases, biologically relevant binding-sites were excluded through the filtering process. The next most promiscuous ligand in Additional file 1: Table S2 is meloxicam, another nonsteroidal anti-inflammatory drug with 110 predicted targets. As in the case of naproxen, the similarities identified are mostly hydrophobic in nature, sometimes with aromatic probes and hydrogen bond donor probes.

The similarities of other promiscuous ligands in Additional file 1: Table S2 also seem to be mainly hydrophobic and this is consistent with previous observations that promiscuity correlates with hydrophobicity [7, 45]. This correlation between promiscuity and hydrophobicity seems to hold for targets as well. While the set of targets of the most promiscuous ligands do not necessarily overlap the set of the most common targets found for all ligands (Additional file 1: Table S4), the most commonly predicted targets have binding-sites that are significantly more hydrophobic and aromatic than the average, as illustrated in Fig. 1.

### Target promiscuity

**Fig. 3:**
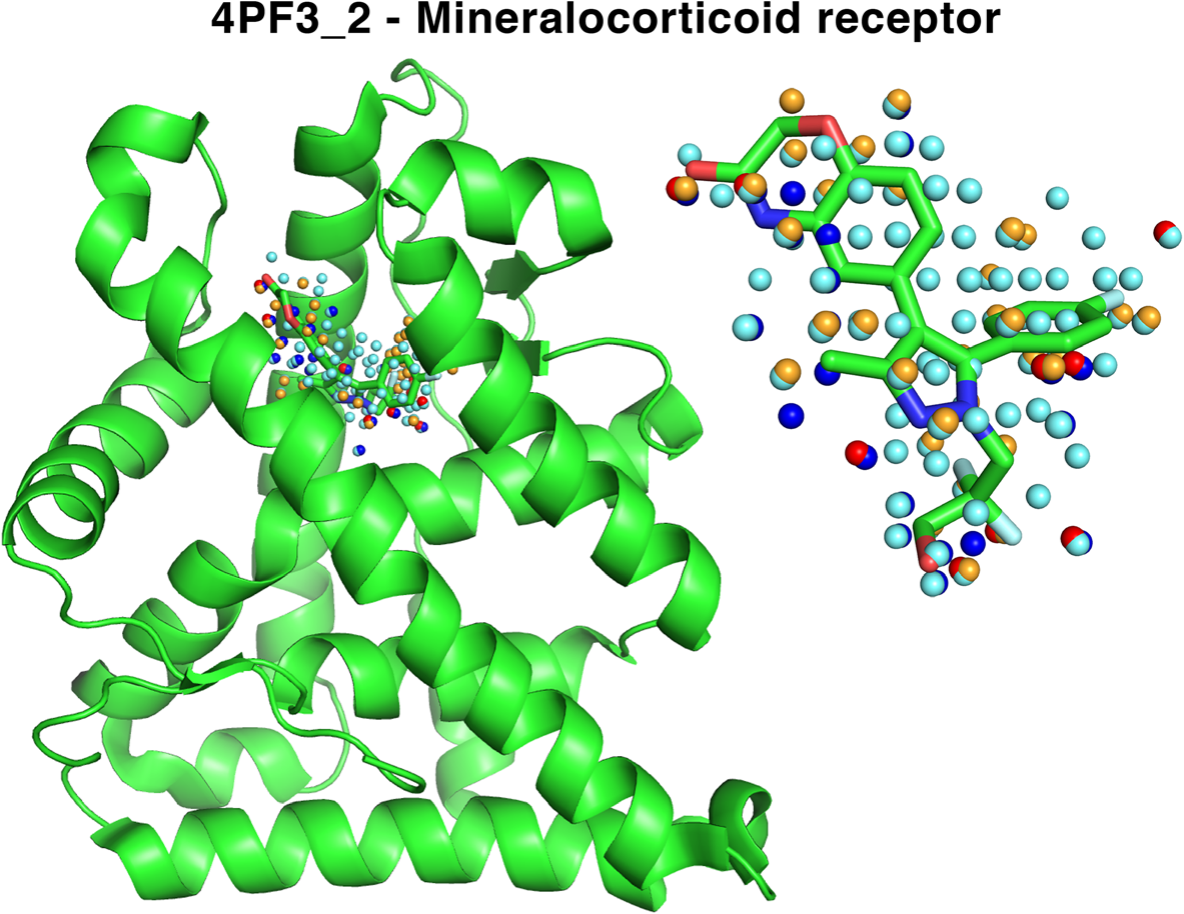
MIF of the mineralocorticoid receptor in the 4PF3_2 binding-site. The MIF is defined in the substrate binding-site and is composed of hydrophobic (*cyan*), aromatic (*orange*), hydrogen bond donor (*blue*) and acceptor (*red*)

Among common predicted targets, the mineralocorticoid nuclear receptor (MR), represented by binding-site 4PF3_2, is a receptor expressed in many human tissues that binds steroid hormones, more specifically mineralocorticoids and glucocorticoids. Aldosterone is the principal hormone that binds to this receptor. The MIF of 4PF3_2 (Fig. 3) is measured in the substrate binding-site where the compound 6-[1-(2,2-difluoro-3-hydroxypropyl)-5-(4-fluorophenyl)-3-methyl-1*H*-pyrazol-4-yl]-2*H*-1,4-benzoxazin-3(4*H*)-one is bound in the crystal structure 4PF3 [46]. The MIF contains hydrogen bond donor and acceptor probes as well as hydrophobic and aromatic probes. Table 1 shows the most commonly predicted ligands for the MR for which the docking simulations yielded an RMSD of less than 3 Å with the pose predicted by IsoMIF. Progesterone is found 6 times, tretinoin 5 times, and testosterone, spironolactone, mifepristone, estradiol and colchicine are predicted 3 times each. The structures of these ligands are shown in Additional file 1: Figure S2. The majority are steroid hormones or structurally similar molecules including approved drugs. For example, spironolactone is an aldosterone antagonist and mifepristone is a glucocorticoid antagonist. From the 55 predictions, 3 predictions of progesterone and 2 of spironolactone were trivial as the query binding-sites of these ligands in the Drugs dataset were derived from structures of MR bound to these ligands.

**Table 1:** Ligands predicted for the mineralocorticoid receptor (4PF3_2)

Ligand^a^	Predictions
Progesterone	6
Tretinoin	5
Testosterone, Spironolactone, Mifepristone, Estradiol, Colchicine	3
Meloxicam, Fluconazole, Diethylstilbestrol, Betamethasone	2
Tadalafil, Podofilox, Pentoxifylline, Papaverine, Liothyronine, Levonorgestrel, Hydrocortisone, Fluticasone furoate, Flurbiprofen, Fludrocortisone, Exemestane, Estrone, Estriol, Cyproterone acetate, Celecoxib, Calcitriol, Caffeine, Bicalutamide, Bexarotene, Atovaquone, Alitretinoin	1

^a^Ligands predicted for the target binding-site 4PF3_2 with Z_3_ for which the RMSD is at most 3 Å

Among the top common targets (Additional file 1: Table S4), rhodopsin II with two binding-sites, 1H2S_6 and 1H2S_7, is predicted 90 and 76 times for the two cavities analysed, respectively. These two cavities are at the surface of chain B (cyan cartoon in Additional file 1: Figure S3), which is responsible for transferring the photo signal into the cytoplasm. These sites are mainly occupied by hydrophobic and aromatic probes as well as donor and acceptor probes. Rhodopsin II is a membrane protein and both cavities 1H2S_6 and 1H2S_7 are not solvent exposed. It highly unlikely that the cavities are biologically relevant or that they may bind so many different ligands. By the nature of hydrophobic cavities, the smaller number of favourable interactions differentiating such cavities lead to an increase in the detection of similarities.

### Drug repurposing candidates

Binding-sites with statistically significant high levels of similarity (Z_3_ cases) that can accommodate the drug based on low RMSD docking poses are a source of potentially interesting drug repurposing hypotheses. Predictions made multiple times for the same drug using different query binding-sites (Additional file +1: Table S3) increase the strength of the prediction as these implicitly account for both variations in binding-site amino-acid composition as well as conformational variability. Some of these cases are discussed in what follows.

#### Acetazolamide as a Chorismate pyruvate lyase inhibitor

**Fig. 4:**
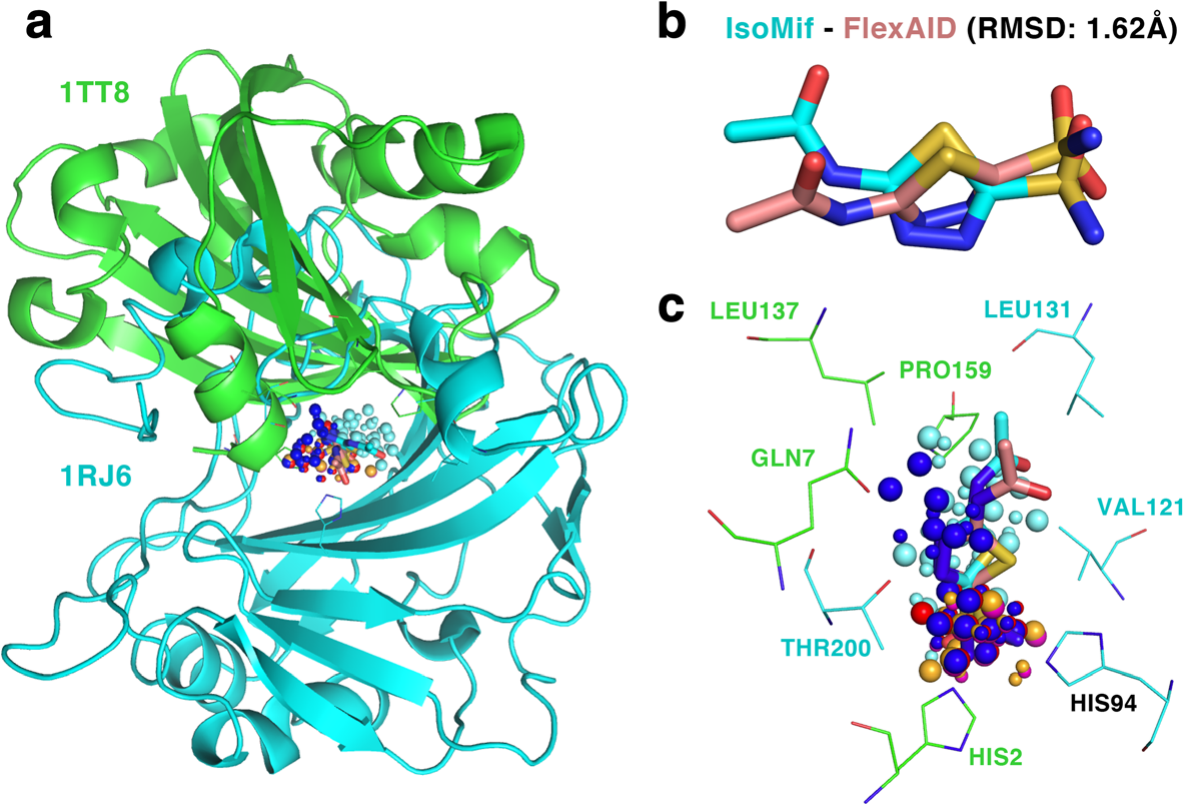
Similarities between carbonic anhydrase and chorismate pyruvate lyase (**a**) Both structures have different folds and are superimposed using the MIF similarities. **b** The IsoMif pose (*cyan*) and the FlexAID pose (salmon), RMSD of 1.62 Å. **c** Similarities for hydrophobic (*cyan*), aromatic (*orange*), donor (*blue*), acceptor (*red*) and negative charge (*magenta*). Large spheres represent probes of 1RJ6 and small ones of 1TT8

Acetazolamide is represented by 14 binding-sites in the Drugs dataset. The Pisces target 1TT8_6 was predicted for 8 of the 14 binding-sites with a Z_3_ threshold and 13 times with a Z_2_ threshold. Target 1TT8_6 represents the chorismate pyruvate lyase protein, present in Gram positive bacteria such as *Escherichia coli* and *Mycobacterium tuberculosis* and is essential for CoQ biosynthesis, an essential cofactor [47]. Fig. 4 shows the superposition of the structures and the detected IsoMIF similarities between a binding-site of acetazolamide (1RJ6_AZM400A-) and 1TT8_6. The figure also shows the ligand poses predicted by IsoMIF (cyan) and FlexAID docking (salmon) with an RMSD of 1.62 Å. Interestingly, despite different CATH structural folds (3.40.1410.10 for 1TT8 and 3.10.200.10 for 1RJ6), many corresponding residues on each structure are found to produce similar MIFs yielding a Tanimoto coefficient of 0.4915 (Z-score 3.3802). Binding-site residues LEU137 and PRO159 (1TT8) as well as LEU131 and VAL121 (1RJ6) yield hydrophobic similarities, GLN7 (1TT8) and THR200 (1RJ6) result in Hydrogen-bond donor similarities and histidine 2 and 94 in 1TT8 and 1RJ6 respectively, produce negatively charged probe similarities. For clarity, not all corresponding residues in the vicinity of the binding-sites are shown. The PyMOL session can be downloaded from the online interface. Whereas the results above point to the possibility that acetazolamide might serve as an antibiotic, the affinity, the effect of acetazolamide on the biosynthesis of CoQ and ultimate its potential as an antibiotic remains to be validated experimentally.

#### Raloxifene and CviR

A second example of repurposing involves a rare pathogen, *Chromobacterium violaceum*, a Gram-negative anaerobic coccobacillus. This pathogen is found in tropical and subtropical regions and was the cause of many deaths in different regions of the globe [48]. *C. violaceum* has a quorum sensing mechanism that allows the activation of virulence genes when the population reaches a certain density. It is a communication mechanism that requires a chemical signal perceived by receptors like LuxR-CviR in the case of *C. violaceum*. The binding of the signal molecules induces homo-dimerization allowing DNA binding and activation the RNA-polymerase. Antagonists of the LuxR-CviR are susceptible to inhibit the quorum sensing mechanism [49]. Raloxifen is a selective estrogen receptor modulator commercialized under the name of Evista and is represented by 4 binding-sites in the drugs Dataset. Table 3 shows that the CviR quorum sensing binding-site (3QP4_1) is a target predicted twice with Z_3_ and twice with Z-scores of 2.99 and 2.82. This is a prediction that wouldn’t be possible with sequence or structure based methods as the two unrelated proteins and have different folds with CATH codes 1.10.565.10 and 3.30.450.80 for the estrogen receptor and CviR respectively.

**Fig. 5:**
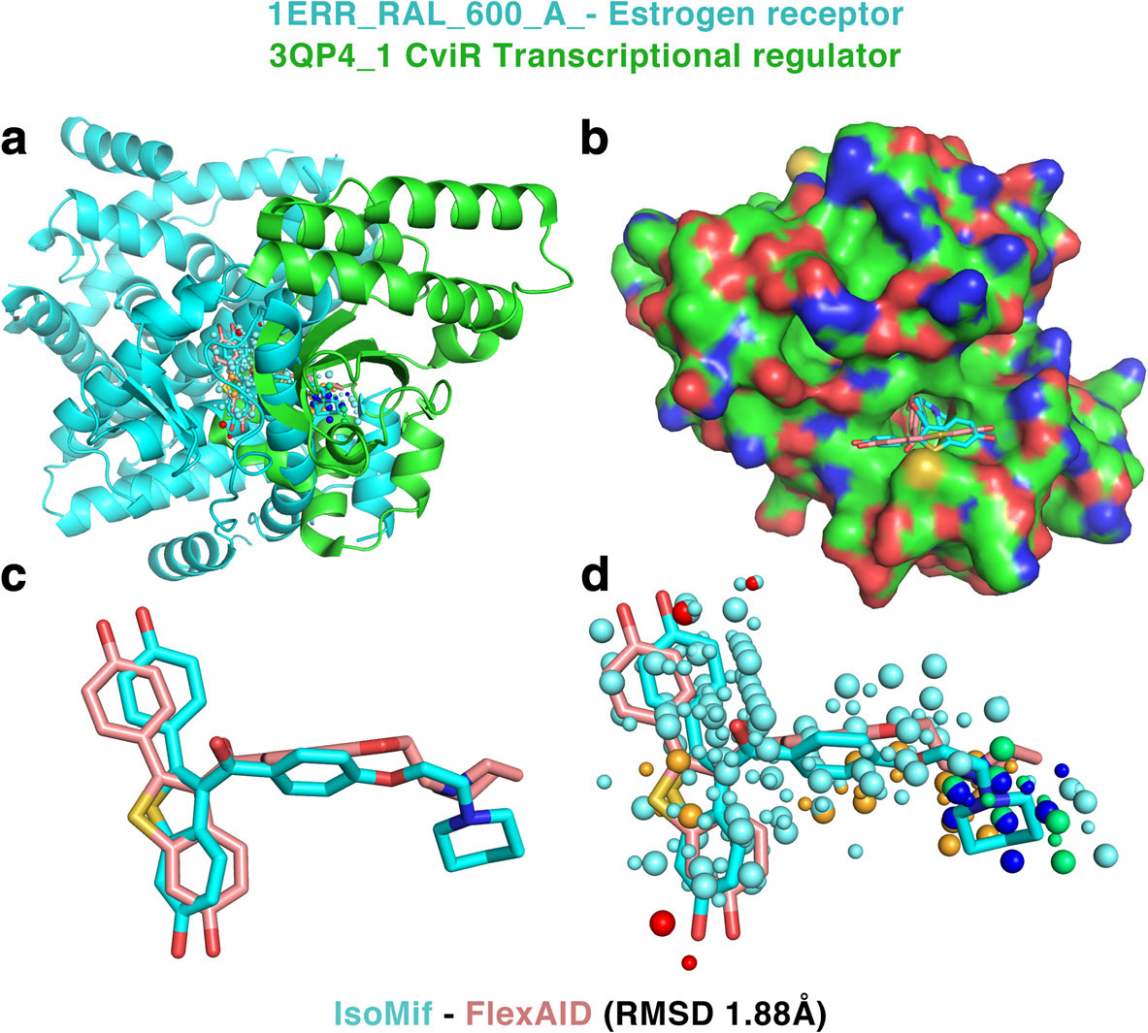
Superimposition of the estrogen receptor and CviR receptor. **a** The two structures of different folds are superimposed using the MIF similarities. **b** A surface representation of 3QP4 shows the deep pocket where raloxifen would bind in CviR. **c** The pose predicted by IsoMIF (*cyan*) and FlexAID (salmon) and **d** the similarities identified by IsoMIF are shown

**Fig. 6:**
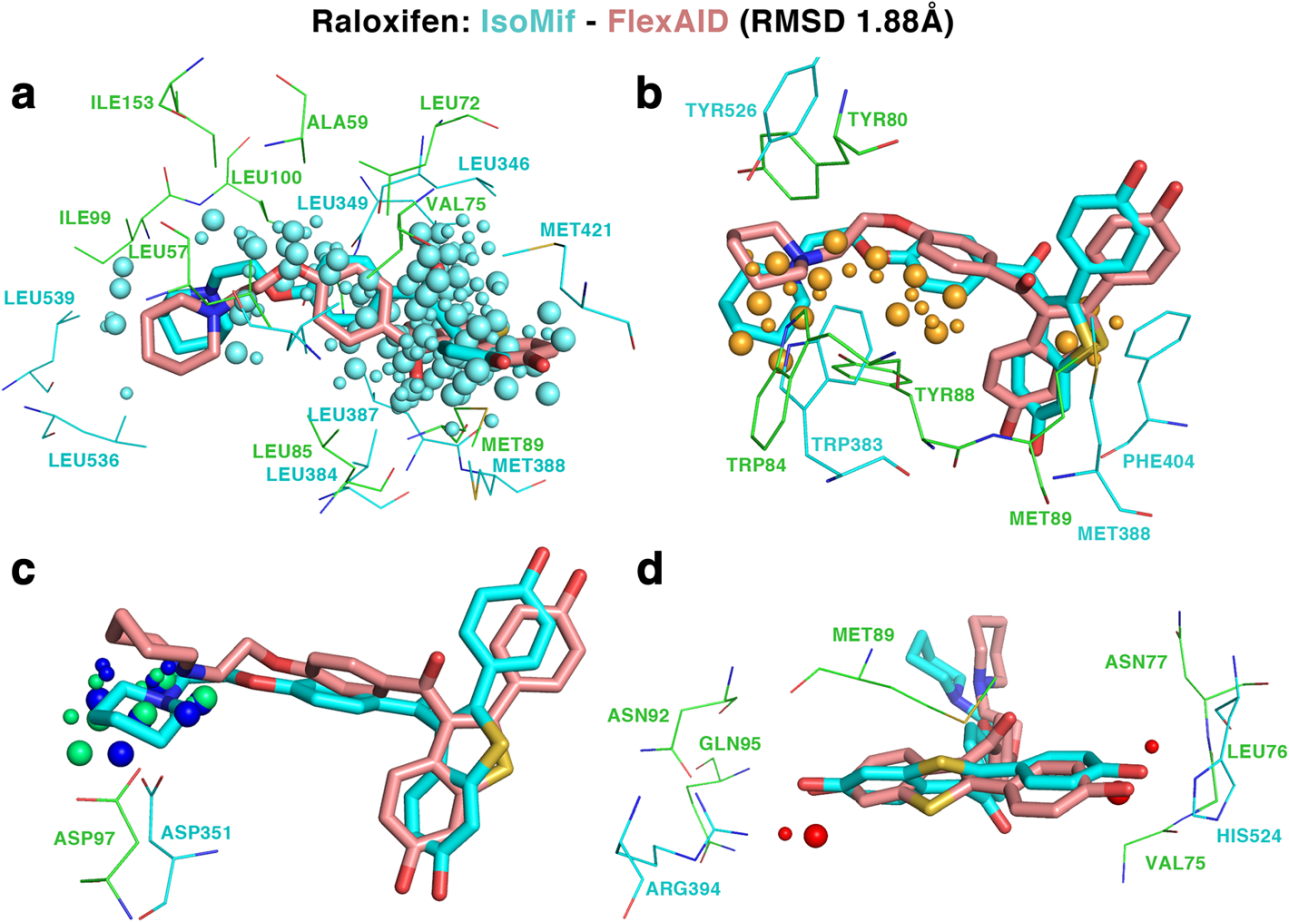
Similarities between the estrogen receptor and the CviR receptor. **a** Hydrophobic similarities are shown in *cyan*, **b** aromatic in *orange*, **c** hydrogen bond donor in *blue* and positive charge in green and **d** hydrogen bond acceptor in *red*

Table 2 shows the similarities between the binding-sites of raloxifen and 3QP4_1 and the results of the docking simulations only for the IsoMIF predictions with Z_3_. The RMSD varies from 1.88 to 4.84 Å and the CF from −103.69 to −238.85. Predictions of raloxifen with a high IsoMIF score and low docking RMSD suggest that raloxifen could bind to the 3QP4_1 binding-site and compete with the signalling molecules, thereby potentially inhibiting the quorum sensing mechanism of gram-negative bacteria. Figure 5 shows the structure of 1ERR_RAL_600_A_- and 3QP4_ 1 superimposed using the MIF similarities and the poses of IsoMIF and FlexAID (1.88 Å). Fig. 6 shows the similarities of different probe types and their underlying residues in both structures. Many corresponding hydrophobic residues create hydrophobic probe similarities mainly in one extremity of raloxifen (Fig. 6a). A pair of tryptophan residues and a pair of tyrosine residues can engage in stacking interactions with the ligand and two corresponding methionine residues suggest sulfur-π interactions. Tyrosine 88 of the CviR receptor does not seem to have a residue that is in a geometrically corresponding position in the query 1ERR binding-site, but could engage in face-to-face stacking with raloxifen (Fig. 6b) after a slight side-chain rearrangement.

**Table 2:** Drugs dataset binding-sites bound to raloxifen found similar to 3QP4_1

Drugs binding-site	Name of the structure	Tanimoto	Z_x_	Pose^a^	RMSD Å	CF
1ERR_RAL_600_A_-	Estrogen receptor	0.3679	3.26	Best RMSD	1.88	−103.69
				Best CF	2.97	−225.67
1QKN_RAL_600_A_-	Estrogen receptor beta	0.3629	3.07	Best RMSD	1.99	−207.33
				Best CF	4.84	−238.85
2QXS_RAL_600_A_-	Estrogen receptor	0.3542	2.99	-	-	-
2JFA_RAL_600_A_-	Estrogen receptor	0.3449	2.82	-	-	-

^a^Indicates if the RMSD and CF information given in the adjacent columns are from the pose with the best RMSD or with the best docking score (CF)

Two aspartates ASP97 and ASP351 could engage in a Hydrogen-bonds with the nitrogen atom of raloxifen (Fig. 6c). Two hydroxyl groups at opposite extremities of raloxifen are stabilized by ARG394 and HIS524 in the binding-site of 1ERR and the corresponding interactions in CviR (3QP4_1) could be made in different ways (Fig. 6d). First, with a Hydrogen-bond involving the backbone amine of GLN95. Second, with a hydrogen bond network involving the carbonyl backbone of MET89, the side chain of ASN92 and potentially a water molecule. On the other side, a hydrogen bond could be made with the backbone of VAL75 and LEU76 or via the side chain of ASN77. In the latter scenario, the side-chain would require to undergo a slight reorientation to optimize contacts upon binding. The poses and side chain arrangement might not represent the ideal conformations as the docking simulations with FlexAID were performed by considering only the ligand structure as flexible.

### Molecular mechanisms responsible for side effects

The detection of binding-site similarities towards the identification of potential candidate cross-reactivity targets for drugs that may be responsible for observed side-effects required matching listed side-effects with equivalent terms associated to the potential cross-reactivity targets through manual inspection. As such, only a few cases are described here.

#### Zanamivir

**Fig. 7:**
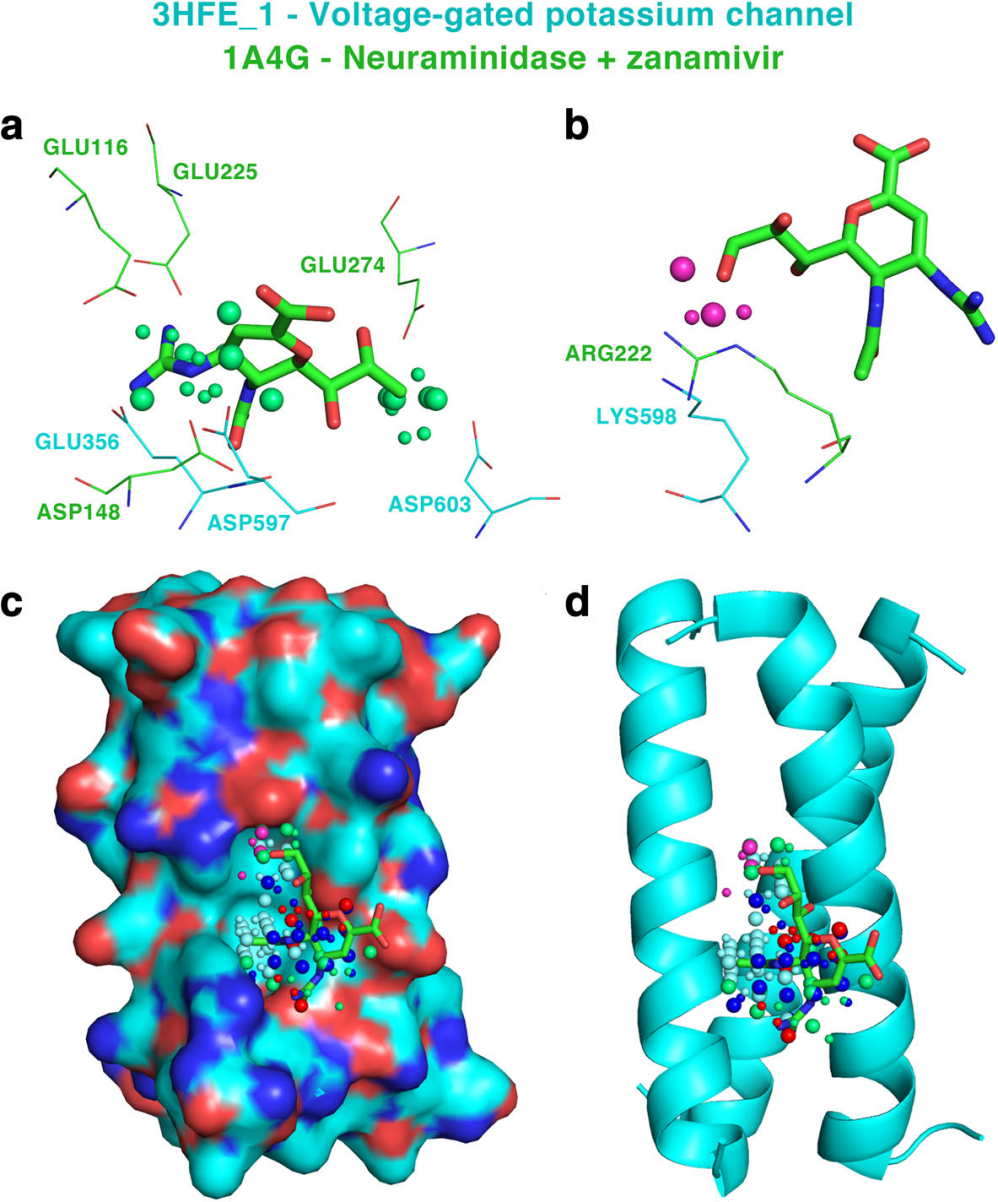
Similarities between the neuraminidase query binding-site and potassium voltage-gated channel 3HFE_1, the potential target. **a** Positively and **b** negatively charged probe similarities. **c** Surface and **d** cartoon representation of a subunit of the channel showing the binding-site of zanamivir and the identified similarities

This drug is an antiviral agent used to treat and prevent influenza. Cardiovascular side effects, including arrhythmias, have been reported spontaneously during post-marketing experience. For the query binding-site 1A4G_ZMR_466_B_- bound to zanamivir, the potential cross-reactivity target binding-site 3HFE_1 is found at rank 19, with a Tanimoto coefficient of 0.3475 (Z-score of 2.6039). The best docking pose of zanamivir on KCNQ1 gives an RMSD of 2.24 Å suggesting that the drug could potentially bind the cross-reactivity binding-site. This structure represents the tail domain of potassium voltage-gated channel KCNQ1 involved in repolarization of cardiac cells and trans-epithelial potassium secretion in the internal ear. Mutations of this gene are associated with the Jervell and Lange-Nielsen syndrome and the Romano-Ward syndrome, recessive and dominant autosomal variants of familial long QT syndrome, respectively, familial atrial fibrillation, and familial short QT syndrome. These rare diseases are all characterized by cardiac arrhythmias. Another mutation in a gene of the same family, KCNQ4, was recently associated to deafness and hearing loss [50]. These associations suggest that the interaction of zanamivir with KCNQ1 (3HFE_1) and perhaps other members of this family such as KCNQ4 could cause the observed side effects. In particular, a single threonine to glutamine amino-acid variation differentiates the region where our analysis suggests that zanamivir could bind to KCNQ1 and that in KCNQ4. The similarities identified for the positively (A) and negatively (B) charged probes and the location of the 3HFE_1 binding-site relative to the whole structure are shown in Fig. 7. The 3HFE structure shows the biological assembly of the domain tail of the potassium channel. The observed side effects of zanamivir could result from the disruption of the assembly of the complex. This could alter potassium flow across the membrane affecting the its repolarization and possibly leading to the observed arrhythmias.

#### Captopril

**Fig. 8:**
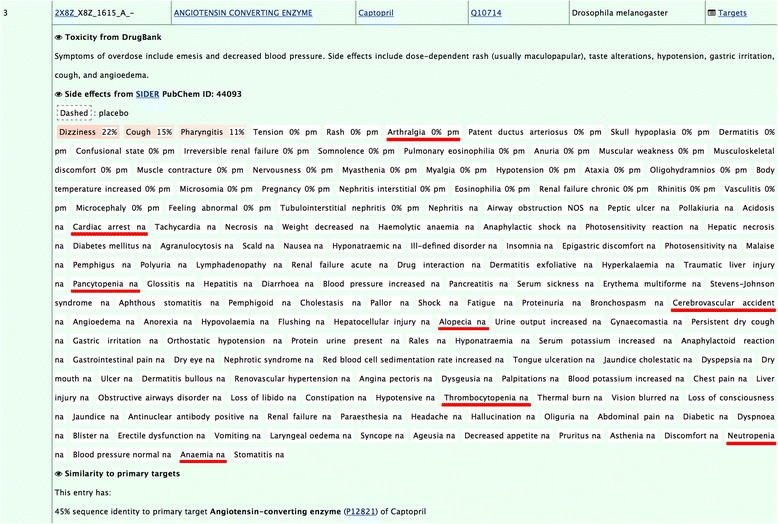
Entry 2X8Z_X8Z_1615_A_- in the online interface showing side effects retrieved from Sider. Hyperlinks bring to external resources: the page of the PDB structure on the RCSB website, Pubmed showing articles where the name of the protein appears in their title, Drugbank page of the drug, Uniprot page, and the side effect resource in Sider. The ‘Targets’ link on the top right leads to the sorted list of predicted targets

The query used is the entry 2X8Z_X8Z_1615_A_- of the drugs dataset represents the angiotensin converting enzyme (ACE) bound to captopril. The reported side effects of captopril include pancytopenia, a deficiency of red blood cells (anemia), white blood cells (leukopenia), and platelets (thrombocytopenia) [51]. The drug was also associated to alopecia [52], cardiac arrest [53], cerebrovascular accidents [54], and arthralgia [55]. These side effects, among others, are retrieved from the Sider database and appear underlined in red in Fig. 8.

**Fig. 9:**
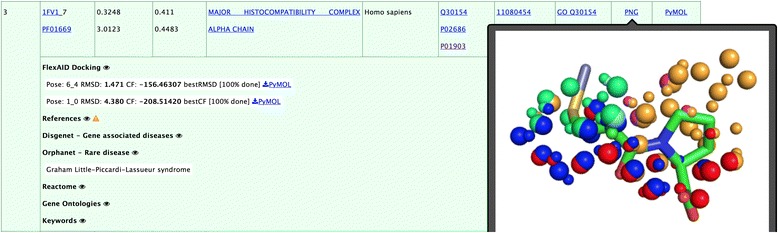
Cross-reactivity target 1FV1_7 identified with Drugs dataset entry 2X8Z_X8Z_1615_A_-. For each off-target, the Tanimoto coefficient and MPC_q_ are given with their Z-score. When available, cross-referenced information can be clicked to expand (Pubmed references, Disgenet, Orphanet, Reactome and Keyowrds in this example). Hovering the mouse on the PNG hyperlink shows a glimpse of the similarities identified by IsoMif and the PyMOL session can be downloaded for the similarities alone and next to the docking results (that contain, in addition to similarities, the docking pose predicted). The information for this off-target is available at the http://bcb.med.usherbrooke.ca/drugs.php?id=2X8Z_X8Z1615A-#1FV1_7

Table 3 shows the top 4 human targets with the highest MIF similarity to ACE. Interestingly, some of these potential cross-reactivity targets are known to be associated to conditions that have phenotypes similar to the observed side effects of the drug. For example, dihydroorotase, found at rank 2 (4C6E_2, Tanimoto coefficient 0.3297, Z-score 3.08), is a protein associated to congenital hypoplastic anemia, an inborn condition characterized by deficiencies of red cell precursors that sometimes also includes leukopenia and thrombocytopenia. Another potential cross-reactivity target found at rank 3 (1FV1_7, Tanimoto coefficient 0.3248, Z-score 3.01) is the alpha chain of the major histocompatibility complex. The data from Orphanet show that the protein is associated to the Graham Little-Piccardi-Lassueur syndrome, which is a disease characterized by cicatricial alopecia of the scalp and noncicatricial alopecia of the axilla and groin. The target is also associated to arthritis. A screenshot of the online interface for the hypothesized cross-reactivity target 1FV1_7, is shown in Fig. 9. The figure also displays how the identified molecular interaction field similarities can be visualized directly from the interface. Finally, tankyrase-2 is found at rank 4 (4PNL_5, Tanimoto coefficient 0.3214, Z-score 2.96) and is associated to cardiovascular diseases and cerebrovascular disorders.

**Table 3:** Top 4 predicted *Homo sapiens* targets for captopril

Rank	Pisces entry	Protein	Tanimoto	Z_x_	Pose^a^	RMSD Å	CF
1	2VIF_2	Suppressor of cytokine signalling 6	0.3503	3.41	Best RMSD	1.08	−66.59
					Best CF	3.86	−187.56
2	4C6E_2	Dihydroorotase	0.3297	3.09	Best RMSD	2.38	−110.18
					Best CF	7.42	−281.23
3	1FV1_7	Major histocompatibility complex alpha chain	0.3248	3.01	Best RMSD	1.47	−156.46
					Best CF	4.38	−208.51
4	4PNL_5	Tankyrase-2	0.3214	2.96	Best RMSD	1.25	−153.22
					Best CF	4.83	−209.89

^a^Indicates if the RMSD and CF information given in the adjacent columns are from the pose with the best RMSD or with the best docking score (CF)

**Fig. 10:**
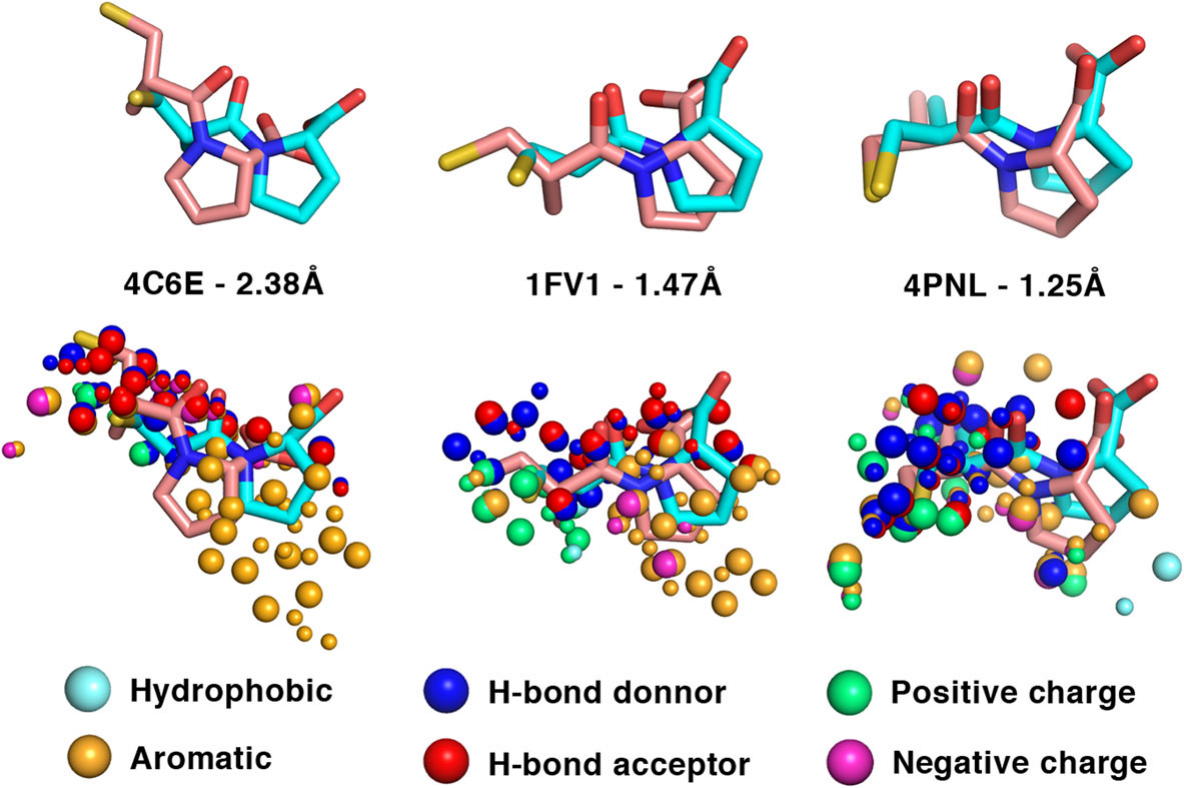
Comparison of captopril docking and ACE MIF similarity based ligand superimposition for the top three captopril cross-reactivity targets. The RMSD is that between ligand poses predicted by FlexAID (salmon) compared to those obtained upon the superimposition of the target and potential cross-reactivity target using the MIF similarities obtained with IsoMIF (*cyan*). The color-coded similarities identified by IsoMIF for specific probe types are shown as spheres. Pairs of large and small spheres represent corresponding (similar) probes in the query and target binding-sites respectively

Table 3 also shows the docking results for the top hits. The best RMSD of the top scored poses predicted by FlexAID for dihydroorotase, the major histocompatibility complex alpha chain and tankyrase-2 are 2.38 Å, 1.47 Å and 1.25 Å, respectively. For these three cross-reactivity targets, Fig. 10 shows the docking pose obtained by FlexAID superimposed to the pose obtained after the superimposition of the MIF similarities (Fig. 10).

## Discussion

The results above highlight some examples obtained from the large-scale analysis of binding-site similarities between drug targets and a non-redundant set of known protein structures. Whereas these examples describe potential drug repurposing candidates or potential molecular explanations for observed side-effects, a number of caveats are in place. First and foremost, the computational data suggest molecular interactions between small-molecules and proteins but as computational hypotheses, must still be validated experimentally.

In the case of drug repurposing hypotheses, the bioavailability of the drug needs to be determined to assess the capacity of the drug to interact with the target. Yet, even if the specific molecule in question may not itself be a good candidate for drug repurposing due to bioavailability issues, it may open novel venues to inhibit the new target.

In the case of hypotheses of mechanisms that explain side-effects, in addition to bioavailability considerations, it is likely that the proposed molecular mechanism is only partially responsible for the side-effect. This seems likely to be so for side-effects that tend to be common and, thus, more likely to arise from several different molecular mechanisms.

From a technical point of view, the fact that some ligands are represented by multiple entries in the Drugs dataset can compensate for certain limitations of binding-site similarity detection methods, specifically conformational differences between the structures of the different binding-sites or the existence of different ligand binding modes. If the performance of ligand-based drug target prediction methods increases when ligand ensembles are used, as in the SEA method, target-based methods that use multiple binding-sites of the same drug could, in principle, yield a higher true positive hit rate when using multiple inputs. Some entries of the original Drugs dataset from RCSB contained unbound structures of primary targets or primary target homologs that were not included in the present work, mainly because the methodology used to define the drug binding-sites required a bound ligand. However, it must be stressed that this is entirely an experimental design choice in the present study and not a requirement to perform binding-site comparisons with IsoMIF. Indeed, whole cavities as defined by GetCleft [36] could have been used.

Because of the non-redundant nature of the Pisces dataset, each binding-site is represented by a single structure. However, including multiple binding-sites from different structures of the same target in the Pisces dataset could be beneficial as conformational changes between structures can affect the detection of similarities. Furthermore, In order to filter out large cavities, a limit of 250 residues in contact with a cavity was used. The reason behind this threshold is that large cavities generate MIFs with many grid vertices and, thus, large association graphs during the search step, increasing significantly the computational time necessary to perform clique detection. More importantly, the whole volume of such large cavities most probably do not represent biologically-relevant binding-sites while sub-regions of these large cavities do. However, as we don’t know in principle where the potentially biologically-relevant subsection of a cavity is, the fact that IsoMIF can handle such large input cavities without affecting the detection of similarities is an advantage in the present study. While this filtering procedure helps us to decrease the computational time required for the detection of similarities and increase the signal to noise ratio, it could exclude potentially interesting binding-sites. Target datasets like the Potential Drug Target Database (PDTD) [56], which contains more than 1100 PDB structures with cross-referenced information or scPDB [57, 58] which represents more than 8000 druggable binding-sites or PDID [59], a database representing 3746 druggable human protein structures could be used in a combined or alternative fashion to the Pisces dataset. Considering that binding-sites are more conserved than other regions of proteins [14] and that the Pisces dataset includes structures of proteins for which there are no human ortholog structures available, the use of the Pisces dataset may help increase the coverage of unique binding-sites. High levels of similarity found with a non-human protein that is a member of a protein family of interest to human health, opens the way to scrutinize all members of that family in detail using the alternative datasets above.

Despite the above limitations regarding the exclusion of potentially important binding-sites and the lack of structural variability in the Pisces target dataset, several hypotheses were proposed regarding potential drug repurposing avenues and side effect mechanisms. The examples presented demonstrate how a target-based MIF similarity method can be used to identify potential off-targets. The results of the docking simulations provide additional information to help assess if the drug could bind the predicted target. The docking predictions leading to small RMSD values represent cases where there are no steric clashes that prevent binding in the off-target binding-site and where the similarities found likely represent important favourable interactions to bind the ligand that are conserved between the target and off-target binding-sites. However, as no docking method is infallible, a consensus docking score using different docking methods could detect false-negative cases that were missed. Furthermore, beyond information regarding the binding of the single molecule of interest (the drug in this case), docking a diverse dataset of small molecules could be used to generate a binding profile that can be compared to the binding-site similarity measure [24]. Finally, docking scores in principle cannot be directly related to binding-affinities but methods such as MM/GBSA [60] try to assess binding free-energies and could provide further information on the potential drug-target interaction. It is interesting to note however that given that 29.6% of cases with high levels of similarities (z-score > 3.0) do have an RMSD below 2.0 Å shows that the important interactions that stabilize a ligand pose in the docking simulation are shared between the two binding-sites.

The off-targets identified could also represent polypharmacological targets if they happen to be associated to a biological process relevant to the same condition. Considering the challenge represented by the design of a potent ligand for a single target, it is reasonable to assume that the probability of finding an already existing ligand that can potently inhibit multiple targets is low. As the ligand would most probably need to go through a medicinal chemistry program to increase selectivity and potency for the polypharmacological target(s), all the required steps involved in drug development such as clinical evaluation of toxicity will be required. Despite this, the data presented here may contain interesting cases from a polypharmacological perspective.

Identifying the molecular causes responsible for the side effects of drugs is a complex task. The phenotype is not necessarily a direct cause of the modulation of an off-target. It could be the result of a cascade of effects across the biological network, sometimes involving the primary target. Systems biology methods, such as flux balance analysis [61] in the case of metabolic networks can be used to suggest if the inhibition of a given protein may be deleterious to the organism [62]. However, the integration of systems and structural methods remains an important challenge in bioinformatics [63]. With the increasing accessibility of exome sequencing, in the future it is likely that we will be able to integrate such data with off-target predictions as generated here towards the goal of precision medicine.

## Conclusions

In this work we utilise the detection of molecular interaction field similarities in what is to our knowledge the first large scale analysis prediction of off-target effects to suggest potential cases of drug repurposing and determine molecular mechanisms responsible for side effects. Drug off-targets were identified in a number of different ways using other binding-site similarity methods based for example on the detection of C-alpha similarities [28], side effect similarity [64] or data mined from social networks [65]. All these predictions can be combined with experimental data and systems biology approaches to yield promising tools to better understand the biological response of an organism exposed to a drug. Lastly, the data generated in the present work represents a useful resource to identify additional cases of protein pairs that may interact with the same small-molecules in order to repurpose existing drugs and understand observed side effects.

## Additional file

Additional file 1: This file contains supplementary figures S1, S2 and S3 as well as supplementary tables S1, S2, S3 and S4. (DOCX 1287 kb)

## Abbreviations

MIF: Molecular Interaction Field
MPC_q_: MIF Probes in Common of the query
PDB: Protein DataBank
RMSD: Root Mean Square Difference
