# Supplementary material for: Large-scale detection of drug off-targets: hypotheses for drug repurposing and understanding side-effects
Source: BMC Pharmacol Toxicol. 2017 Apr 28;18:18. doi: 10.1186/s40360-017-0128-7 (PMC5408384; doi:10.1186/s40360-017-0128-7)
Supplement: Additional file 1: — This file contains supplementary figures S1, S2 and S3 as well as supplementary tables S1, S2, S3 and S4. (DOCX 1287 kb) [file 40360_2017_128_MOESM1_ESM.docx]

**Supplementary data for**

**Large-scale detection of drug off-targets: Hypotheses for drug repurposing and understanding side-effects**

Matthieu Chartier, Louis-Philippe Morency, María Inés Zylber & Rafael J. Najmanovich*

*corresponding author: rafael.najmanovich@umontreal.ca


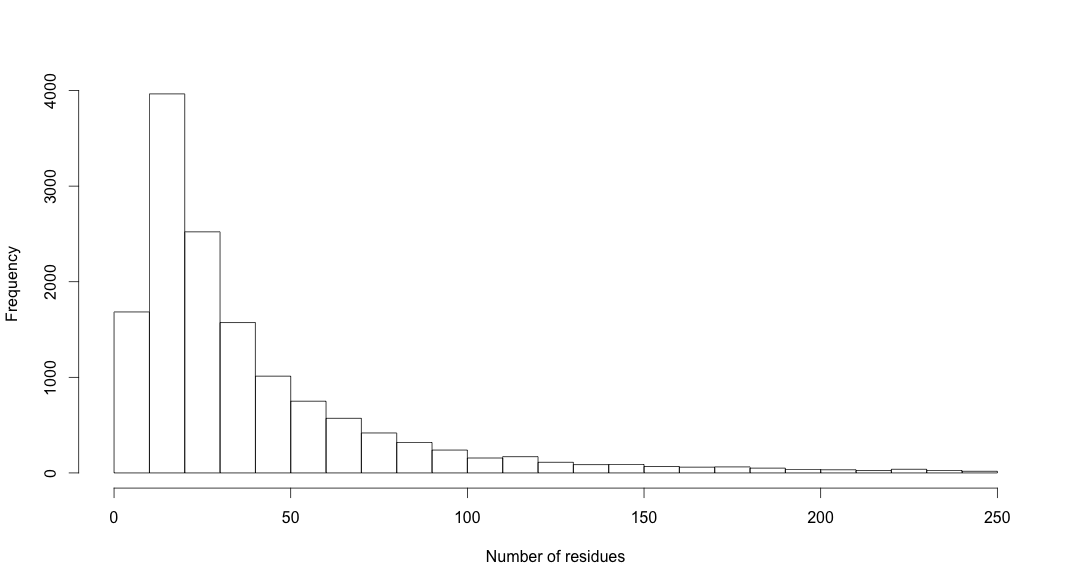


**Figure S1**. **Distribution of number of residues defining Pisces cavities**. The average number of residues is 39.


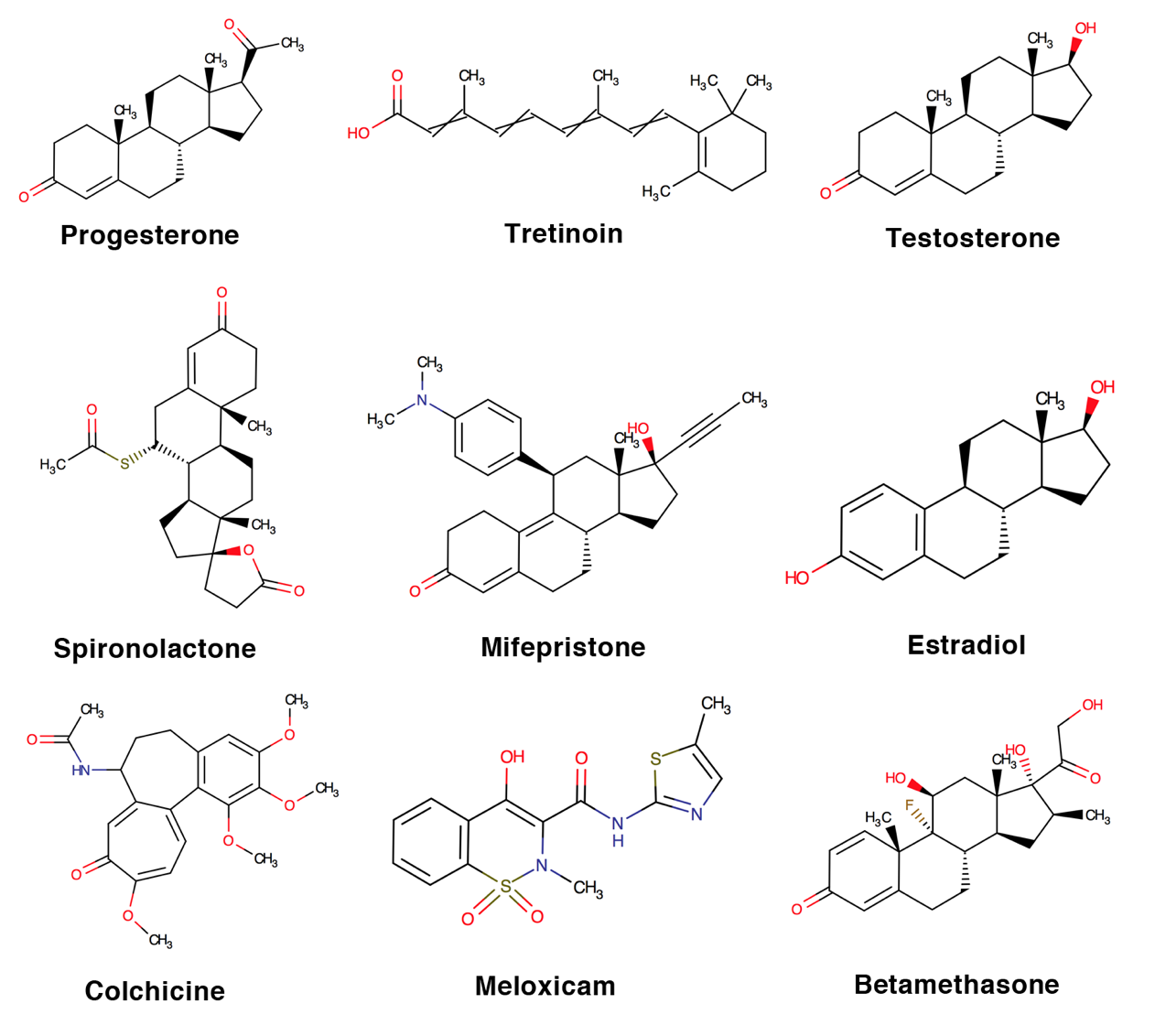


Figure S2 – Structures of ligands predicted for 4PF3_2. Some of the ligands predicted for the 4PF2_2 binding site of the mineralocorticoid receptor.


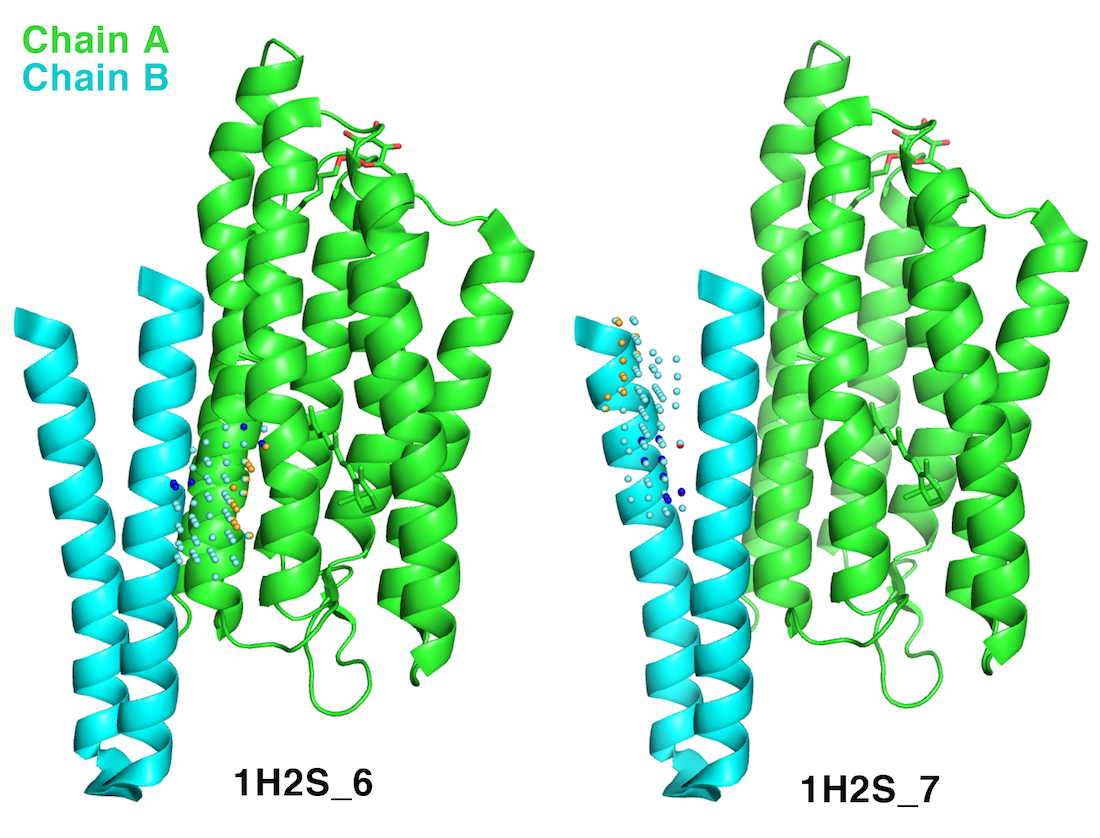


Figure S3 – Rhodopsin II binding sites. The two binding sites of the sensory rhodopsin II in contact with chain B (cyan) of PDB structure 1H2S.

Table S1 – Non-redundant list of ligands of the Drugs dataset

| **Drugbank** | **Ligand** | **Nb. of entries^a^** | **Targets^b^** | **SE^c^** | **SE >5%^d^** | **Drugs entries** |
| --- | --- | --- | --- | --- | --- | --- |
| DB00819 | Acetazolamide | 14 | 348 | 74 | 0 | 1RJ6_AZM400A-,1JD0_AZM1400A-,1ZSB_AZM264A-,1AZM_AZM262A-,4LU3_AZM302A-,3W6H_AZM303A-,1YDA_AZM264A-,3V2M_AZM303A-,3DC3_AZM263A-,3ML5_AZM264A-,1YDB_AZM264A-,3CZV_AZM263A-,3V2J_AZM302A-,3HS4_AZM701A- |
| DB00755 | Tretinoin | 7 | 527 | 301 | 101 | 3LBD_REA424A-,4DM8_REA501A-,3OAP_REA500A-,2LBD_REA500A-,1G5Y_REA502C-,1XLS_REA801A-,3FC6_REA501C- |
| DB00558 | Zanamivir | 6 | 56 | 70 | 16 | 4CPZ_ZMR1470C-,4I00_ZMR509A-,2CML_ZMR4477D-,1A4G_ZMR466B-,4CPN_ZMR700A-,3TIC_ZMR1002C- |
| DB00396 | Progesterone | 6 | 304 | 317 | 40 | 4FN9_STR301A-,1YA3_STR3001C-,4LTW_STR301A-,1A28_STR1A-,2AA6_STR401A-,2AA5_STR301A- |
| DB00877 | Sirolimus | 6 | 10 | 0 | 0 | 1FAP_RAP108A-,1FKB_RAP108A-,4DRJ_RAP201A-,4DRI_RAP201A-,2DG4_RAP501A-,4DRH_RAP201A- |
| DB00279 | Liothyronine | 6 | 82 | 12 | 0 | 2H79_T31A-,4LNW_T3501A-,3GWS_T3500X-,1BSX_T31A-,1XZX_T3500X-,4LNX_T3502A- |
| DB02546 | Vorinostat | 6 | 55 | 68 | 0 | 3C0Z_SHH301B-,1ZZ1_SHH2452A-,1C3S_SHH952A-,4QA2_SHH404A-,4LXZ_SHH408B-,4QA0_SHH404A- |
| DB00783 | Estradiol | 6 | 277 | 447 | 85 | 3OLS_EST600B-,4J24_EST600C-,2YJA_EST1550B-,3OLL_EST600B-,3UUD_EST600B-,2OCF_EST596A- |
| DB00360 | Tetrahydrobiopterin | 6 | 39 | 57 | 22 | 4D1O_H4B1481B-,1J8U_H4B429A-,1MMK_H4B1427A-,4D1P_H4B600B-,1KW0_H4B429A-,3NOS_H4B511A- |
| DB08865 | Crizotinib | 5 | 33 | 0 | 0 | 3ZBF_VGH3000A-,2WGJ_VGH2346A-,4ANQ_VGH9000A-,2XP2_VGH9000A-,2YFX_VGH9000A- |
| DB00140 | Riboflavin | 5 | 2 | 0 | 0 | 1HZE_RBF99B-,1NB9_RBF401A-,4E0F_RBF301A-,1T6Z_RBF596B-,1PKV_RBF101B- |
| DB01254 | Dasatinib | 5 | 41 | 292 | 53 | 2ZVA_1N1513A-,2GQG_1N1501A-,2Y6O_1N11892A-,3G5D_1N11A-,3QLG_1N1601AA |
| DB00398 | Sorafenib | 5 | 5 | 201 | 44 | 1UWJ_BAX1723B-,3HEG_BAX1A-,4ASD_BAX1500A-,3GCS_BAX401A-,1UWH_BAX1723B- |
| DB00513 | Aminocaproic Acid | 5 | 220 | 55 | 0 | 3KIV_ACA100A-,1CEA_ACA90A-,1HPK_ACA80A-,2PK4_ACA100A-,1PK2_ACA91A- |
| DB00205 | Pyrimethamine | 5 | 4 | 15 | 0 | 1J3J_CP6709B-,2BLA_CP61240A-,3QFX_CP6602A-,3UM5_CP6709B-,4KM0_CP6201B- |
| DB00311 | Ethoxzolamide | 4 | 28 | 0 | 0 | 3CAJ_EZL265A-,3DD0_EZL301A-,3MDZ_EZL264A-,3DCW_EZL301A- |
| DB00834 | Mifepristone | 4 | 97 | 92 | 26 | 1NHZ_486800A-,4LTW_486303A-,3H52_4864C-,2W8Y_4861000A- |
| DB00284 | Acarbose | 4 | 4 | 0 | 0 | 3TOP_ACR1B-,3W37_ACR1001A-,1KXH_ACR598A-,2QMJ_ACR1001A- |
| DB00703 | Methazolamide | 4 | 55 | 59 | 0 | 3DCS_MZM263A-,1BZM_MZM262A-,3DAZ_MZM263A-,4K0Z_MZM308A- |
| DB00399 | Zoledronate | 4 | 77 | 235 | 58 | 2E91_ZOL901A-,3N45_ZOL354F-,2F9K_ZOL9001F-,4KFA_ZOL404A- |
| DB00481 | Raloxifene | 4 | 34 | 99 | 33 | 1ERR_RAL600A-,1QKN_RAL600A-,2QXS_RAL600A-,2JFA_RAL600A- |
| DB00642 | Pemetrexed | 3 | 7 | 126 | 46 | 1JU6_LYA315B-,3K2H_LYA513A-,1JUJ_LYA315B- |
| DB00136 | Calcitriol | 3 | 70 | 0 | 0 | 1DB1_VDX428A-,1IE9_VDX500A-,3M7R_VDX425A- |
| DB00862 | Vardenafil | 3 | 25 | 169 | 3 | 1XP0_VDN201A-,3B2R_VDN1A-,1UHO_VDN1000A- |
| DB00619 | Imatinib | 3 | 47 | 358 | 123 | 1XBB_STI1A-,1T46_STI3A-,2PL0_STI200A- |
| DB00722 | Lisinopril | 3 | 33 | 0 | 0 | 2X91_LPR1615A-,2C6N_LPR705B-,1O86_LPR702A- |
| DB01045 | Rifampicin | 3 | 1 | 0 | 0 | 4KMU_RFP1401C-,1I6V_RFP1640C-,1YNN_RFP1120C- |
| DB00864 | Tacrolimus | 3 | 13 | 0 | 0 | 1TCO_FK5509C-,2FKE_FK5108A-,1FKF_FK5108A- |
| DB01092 | Ouabain | 3 | 0 | 0 | 0 | 3A3Y_OBN6000A-,3N23_OBN1C-,4HYT_OBN2004C- |
| DB00196 | Fluconazole | 3 | 159 | 161 | 5 | 2WUZ_TPF1460B-,3KHM_TPF501A-,2WX2_TPF1460B- |
| DB05812 | Abiraterone | 3 | 115 | 0 | 0 | 3RUK_AER601D-,4R1Z_AER601A-,4NKV_AER601D- |
| DB00712 | Flurbiprofen | 3 | 254 | 257 | 0 | 2AYL_FLP2701BA,3PGH_FLP701C-,3RR3_FLR700A- |
| DB00696 | Ergotamine | 3 | 4 | 24 | 0 | 4NC3_ERM1202A-,4IB4_ERM2001A-,4IAR_ERM2001A- |
| DB08901 | Ponatinib | 3 | 1 | 0 | 0 | 3IK3_0LI1A-,3OXZ_0LI1A-,4UXQ_0LI1752A- |
| DB00328 | Indomethacin | 3 | 3 | 201 | 7 | 4COX_IMN701B-,3FO7_IMN301A-,2OTH_IMN301A- |
| DB00293 | Raltitrexed | 3 | 25 | 56 | 21 | 1I00_D16409B-,1HVY_D16414A-,4EB4_D16402A- |
| DB01229 | Paclitaxel | 3 | 8 | 0 | 0 | 3J6G_TA1502B-,2HXF_TA1601B-,1JFF_TA1601B- |
| DB00869 | Dorzolamide | 3 | 23 | 0 | 0 | 4K13_ETS304A-,4M2U_ETS302A-,1CIL_ETS263A- |
| DB01024 | Mycophenolic acid | 3 | 0 | 407 | 75 | 4AF0_MOA1526B-,1JR1_MOA1332A-,4FO4_MOA502B- |
| DB01051 | Novobiocin | 3 | 15 | 0 | 0 | 4URN_NOV2000A-,1AJ6_NOV1A-,4URO_NOV2000A- |
| DB00382 | Tacrine | 3 | 67 | 0 | 0 | 1ACJ_THA999A-,1MX1_THA2BA,4BDS_THA701A- |
| DB00421 | Spironolactone | 3 | 130 | 56 | 0 | 3VHU_SNL1001A-,2OAX_SNL5001E-,2AB2_SNL502A- |
| DB00412 | Rosiglitazone | 3 | 9 | 0 | 0 | 1ZGY_BRL503A-,3DZY_BRL478D-,1FM6_BRL504X- |
| DB01128 | Bicalutamide | 3 | 74 | 202 | 45 | 4OJB_1981001A-,1Z95_198501A-,4OK1_1981001A- |
| DB01197 | Captopril | 3 | 138 | 148 | 3 | 2X8Z_X8Z1615A-,1J37_X8Z801A-,4C2P_X8Z709A- |
| DB00624 | Testosterone | 3 | 204 | 408 | 21 | 2AM9_TES1000A-,2Q7K_TES304A-,2Q7I_TES205A- |
| DB00674 | Galantamine | 3 | 47 | 0 | 0 | 1W6R_GNT1536A-,4EY6_GNT604A-,1DX6_GNT1537A- |
| DB01367 | Rasagiline | 3 | 78 | 0 | 0 | 2C72_RSA601A-,2C75_RSA601A-,2C73_RSA601A- |
| DB01409 | Tiotropium | 3 | 73 | 0 | 0 | 4U14_0HK2000A-,4DAJ_0HK2000C-,4U15_0HK2001A- |
| DB00282 | Pamidronate | 3 | 68 | 0 | 0 | 2F89_2109001F-,4KPJ_210901A-,4NKF_210404A- |
| DB00443 | Betamethasone | 3 | 29 | 143 | 0 | 3MNE_DEX784A-,1M2Z_DEX301A-,1P93_DEX4999D- |
| DB06616 | Bosutinib | 3 | 24 | 0 | 0 | 4MXY_DB8601A-,4MXO_DB8601A-,3UE4_DB8601A- |
| DB00630 | Alendronate | 3 | 55 | 94 | 2 | 2F92_AHD9001F-,4KQU_AHD405A-,1YHM_AHD901A- |
| DB00951 | Isoniazid | 3 | 248 | 54 | 0 | 4KA6_NIZ805A-,3N3P_NIZ749B-,4KWQ_NIZ815B- |
| DB00563 | Methotrexate | 3 | 1 | 321 | 17 | 1DLS_MTX188A-,1U72_MTX188A-,3EIG_MTX200A- |
| DB00203 | Sildenafil | 3 | 14 | 344 | 30 | 3JWQ_VIA901D-,1TBF_VIA501A-,1UDT_VIA1000A- |
| DB01268 | Sunitinib | 3 | 29 | 329 | 17 | 3G0F_B499001A-,4AGD_B492000A-,3G0E_B499000A- |
| DB00884 | Risedronate | 3 | 18 | 165 | 38 | 4KQS_RIS405A-,4KPD_RIS405A-,2QIS_RIS901A- |
| DB00551 | Acetohydroxamic Acid | 3 | 617 | 37 | 1 | 4UBP_HAE800C-,1FWE_HAE989C-,1E9Y_HAE800B- |
| DB01394 | Colchicine | 3 | 227 | 0 | 0 | 3E22_LOC700D-,3UT5_LOC502B-,4O2B_LOC503B- |
| DB01194 | Brinzolamide | 3 | 16 | 185 | 3 | 3ZNC_BZ1500A-,4M2V_BZ1302A-,4M2R_BZ1302A- |
| DB00898 | Ethanol | 3 | 749 | 0 | 0 | 1ADB_EOH378B-,1ADC_EOH378B-,1F8F_EOH374A- |
| DB00317 | Gefitinib | 3 | 15 | 129 | 36 | 2ITY_IRE2020A-,3UG2_IRE1A-,4WKQ_IRE1101A- |
| DB00278 | Argatroban | 3 | 5 | 0 | 0 | 4HFP_15U402B-,1ETR_MIT1H-,1DWC_MIT1H- |
| DB00159 | Icosapent | 3 | 26 | 0 | 0 | 3HS6_EPA1B-,1IGX_EPA700A-,3GWX_EPA3BA |
| DB08809 | Dichloroacetic Acid | 2 | 440 | 0 | 0 | 2BU8_TF41379A-,2Q8H_TF4438A- |
| DB00509 | Dextrothyroxine | 2 | 38 | 0 | 0 | 1Y0X_T44500X-,4LNX_T44501A- |
| DB00843 | Donepezil | 2 | 4 | 348 | 21 | 1EVE_E202001A-,4EY7_E20604A- |
| DB01248 | Docetaxel | 2 | 10 | 0 | 0 | 1TUB_TXL501B-,1IA0_TXL502B- |
| DB00841 | Dobutamine | 2 | 0 | 16 | 0 | 2Y00_Y00601B-,2Y01_Y00601B- |
| DB00273 | Topiramate | 2 | 35 | 540 | 109 | 3HKU_TOR300A-,3LXE_TOR262A- |
| DB00820 | Tadalafil | 2 | 49 | 157 | 31 | 1XOZ_CIA501A-,1UDU_CIA1003A- |
| DB00218 | Moxifloxacin | 2 | 27 | 0 | 0 | 2XKK_MFX1100E-,3FOF_MFX0F- |
| DB01157 | Trimetrexate | 2 | 22 | 0 | 0 | 4M2X_TMQ202C-,3CLB_TMQ611A- |
| DB01261 | Sitagliptin | 2 | 5 | 129 | 2 | 4FFW_715801B-,1X70_715801B- |
| DB00302 | Tranexamic Acid | 2 | 168 | 0 | 0 | 1CEB_AMH90B-,1B2I_AMH84A- |
| DB00814 | Meloxicam | 2 | 198 | 0 | 0 | 4M11_MXM606BA,4O1Z_MXM807AA |
| DB00936 | Salicylic acid | 2 | 307 | 49 | 0 | 1PTH_SAL711B-,3N8Y_SAL900BA |
| DB01010 | Edrophonium | 2 | 76 | 31 | 0 | 1AX9_EDR999A-,2ACK_EDR999A- |
| DB01199 | Tubocurarine | 2 | 5 | 0 | 0 | 2XYT_TC91206B-,3PMZ_TUB220E- |
| DB00147 | Pyridoxal | 2 | 58 | 0 | 0 | 1TD2_PXL288A-,3FHX_PXL313B- |
| DB00530 | Erlotinib | 2 | 2 | 153 | 36 | 1M17_AQ4999A-,4HJO_AQ41001A- |
| DB00255 | Diethylstilbestrol | 2 | 101 | 0 | 0 | 1S9P_DES459B-,3ERD_DES600A- |
| DB01050 | Ibuprofen | 2 | 142 | 337 | 41 | 1EQG_IBP1701B-,4PH9_IBP601A- |
| DB08915 | Aleglitazar | 2 | 42 | 0 | 0 | 3G9E_RO71A-,3G8I_RO71A- |
| DB00523 | Alitretinoin | 2 | 159 | 0 | 0 | 1K74_9CR463A-,1FM6_9CR501A- |
| DB00323 | Tolcapone | 2 | 60 | 275 | 29 | 3S68_TCW227A-,4PYL_TCW303A- |
| DB00188 | Bortezomib | 2 | 0 | 0 | 0 | 2F16_BO21402K-,3MG0_BO21402K- |
| DB01259 | Lapatinib | 2 | 4 | 74 | 39 | 1XKK_FMM91A-,3BBT_FMM91B- |
| DB00570 | Vinblastine | 2 | 0 | 0 | 0 | 4EB6_VLB503C-,1Z2B_VLB800C- |
| DB01127 | Econazole | 2 | 30 | 6 | 0 | 3JUS_ECL600BB,3JUS_ECN602AA |
| DB01137 | Levofloxacin | 2 | 30 | 389 | 0 | 4JUO_LFX101F-,3K9F_LFX0H- |
| DB00127 | Spermine | 2 | 13 | 0 | 0 | 3C6M_SPM501C-,3KU9_SPM700A- |
| DB00482 | Celecoxib | 2 | 61 | 346 | 26 | 3LN1_CEL682A-,3KK6_CEL701A- |
| DB00586 | Diclofenac | 2 | 55 | 0 | 0 | 3N8Y_DIF585BB,1PXX_DIF701A- |
| DB08881 | Vemurafenib | 1 | 1 | 0 | 0 | 3OG7_0321A- |
| DB00181 | Baclofen | 1 | 33 | 236 | 29 | 4MS4_2C0501A- |
| DB04573 | Estriol | 1 | 23 | 30 | 0 | 3Q95_ESL700B- |
| DB00259 | Sulfanilamide | 1 | 1 | 2 | 0 | 1AJ0_SAN561A- |
| DB01331 | Cefoxitin | 1 | 14 | 66 | 0 | 3MZE_CFX364A- |
| DB01015 | Sulfamethoxazole | 1 | 33 | 91 | 0 | 3TZF_08D280B- |
| DB01004 | Ganciclovir | 1 | 13 | 0 | 0 | 1KI2_GA21A- |
| DB00148 | Creatine | 1 | 18 | 0 | 0 | 3B6R_CRN603B- |
| DB00710 | Ibandronate | 1 | 1 | 203 | 22 | 2F94_BFQ9001FA |
| DB01764 | Dalfopristin | 1 | 0 | 0 | 0 | 1MRL_DOL302C- |
| DB01201 | Rifapentine | 1 | 0 | 218 | 0 | 2A69_RPT8001C- |
| DB01026 | Ketoconazole | 1 | 15 | 0 | 0 | 3LD6_KKK602B- |
| DB00540 | Nortriptyline | 1 | 3 | 153 | 0 | 4M48_21B704A- |
| DB01273 | Varenicline | 1 | 12 | 0 | 0 | 4AFG_QMR1214A- |
| DB00367 | Levonorgestrel | 1 | 50 | 220 | 29 | 3D90_NOG2001B- |
| DB09030 | Vorapaxar | 1 | 3 | 0 | 0 | 3VW7_VPX2001A- |
| DB06693 | Mevastatin | 1 | 0 | 0 | 0 | 1HW8_1142A- |
| DB01536 | 4-Androstenedione | 1 | 1 | 0 | 0 | 1QYX_ASD500A- |
| DB00307 | Bexarotene | 1 | 55 | 291 | 121 | 3H0A_9RA500A- |
| DB00668 | Epinephrine | 1 | 3 | 0 | 0 | 4LDO_ALE1402A- |
| DB00773 | Etoposide | 1 | 0 | 155 | 26 | 3QX3_EVP1D- |
| DB04552 | Niflumic Acid | 1 | 2 | 0 | 0 | 1TD7_NFL2001A- |
| DB00552 | Pentostatin | 1 | 16 | 179 | 51 | 1A4L_DCF1353C- |
| DB06335 | Saxagliptin | 1 | 22 | 58 | 3 | 3BJM_BJM2B- |
| DB01204 | Mitoxantrone | 1 | 0 | 177 | 86 | 4G0V_MIX1301A- |
| DB01034 | Cerulenin | 1 | 16 | 0 | 0 | 4LS7_1X9504A- |
| DB01283 | Lumiracoxib | 1 | 59 | 0 | 0 | 4OTY_LUR705A- |
| DB00787 | Aciclovir | 1 | 35 | 178 | 10 | 2KI5_AC21AA |
| DB00201 | Caffeine | 1 | 93 | 59 | 2 | 3RFM_CFF330A- |
| DB01136 | Carvedilol | 1 | 0 | 251 | 43 | 4AMJ_CVD1359A- |
| DB01064 | Isoprenaline | 1 | 2 | 29 | 0 | 2Y03_5FW601A- |
| DB00788 | Naproxen | 1 | 122 | 0 | 0 | 3NT1_NPS4B- |
| DB06700 | Desvenlafaxine | 1 | 10 | 110 | 26 | 4MM7_29J603A- |
| DB00640 | Adenosine | 1 | 25 | 111 | 11 | 2YDO_ADN400A- |
| DB00806 | Pentoxifylline | 1 | 46 | 111 | 11 | 3TVX_PNX902B- |
| DB01076 | Atorvastatin | 1 | 0 | 0 | 0 | 1HWK_1173D- |
| DB00730 | Thiabendazole | 1 | 3 | 53 | 0 | 3SFE_TMG1C- |
| DB00655 | Estrone | 1 | 63 | 74 | 0 | 3HM1_J3Z2A- |
| DB04839 | Cyproterone acetate | 1 | 44 | 0 | 0 | 2OZ7_CA41A- |
| DB00763 | Methimazole | 1 | 62 | 36 | 0 | 4IG5_MMZ617B- |
| DB00741 | Hydrocortisone | 1 | 4 | 231 | 9 | 4P6X_HCY900I- |
| DB01597 | Cilastatin | 1 | 31 | 0 | 0 | 1ITU_CIL451A- |
| DB00775 | Tirofiban | 1 | 2 | 0 | 0 | 2VDM_AGG1462B- |
| DB00687 | Fludrocortisone | 1 | 28 | 86 | 0 | 1GS4_ZK51918A- |
| DB00152 | Thiamine | 1 | 4 | 21 | 0 | 1IG3_VIB502A- |
| DB01167 | Itraconazole | 1 | 0 | 232 | 25 | 4K0F_1YN602A- |
| DB00380 | Dexrazoxane | 1 | 24 | 105 | 22 | 1QZR_CDX901BA |
| DB00154 | Dihomo-Î³-linolenic acid | 1 | 4 | 0 | 0 | 1FE2_LAX700A- |
| DB08877 | Ruxolitinib | 1 | 20 | 0 | 0 | 4U5J_RXT601B- |
| DB00428 | Streptozocin | 1 | 9 | 58 | 7 | 2W4X_STZ1591A- |
| DB00476 | Duloxetine | 1 | 4 | 0 | 0 | 4MM6_29E603A- |
| DB00685 | Trovafloxacin | 1 | 0 | 212 | 3 | 4KOE_TR6101F- |
| DB08906 | Fluticasone furoate | 1 | 32 | 0 | 0 | 3CLD_GW61A- |
| DB00615 | Rifabutin | 1 | 0 | 82 | 12 | 2A68_RBT8001C- |
| DB01142 | Doxepin | 1 | 10 | 0 | 0 | 3RZE_D7V1201AB |
| DB04743 | Nimesulide | 1 | 10 | 0 | 0 | 3N8X_NIM701A- |
| DB01117 | Atovaquone | 1 | 89 | 72 | 39 | 4PD4_AOQ4003C- |
| DB00260 | Cycloserine | 1 | 102 | 0 | 0 | 1XQL_4AX505BE |
| DB00299 | Penciclovir | 1 | 5 | 57 | 6 | 1KI3_PE21A- |
| DB04835 | Maraviroc | 1 | 0 | 271 | 20 | 4MBS_MRV1101B- |
| DB06605 | Apixaban | 1 | 1 | 102 | 0 | 2P16_GG2298A- |
| DB01606 | Tazobactam | 1 | 13 | 0 | 0 | 1VM1_TAZ504A- |
| DB00491 | Miglitol | 1 | 30 | 23 | 0 | 3L4W_MIG1001A- |
| DB01586 | Ursodeoxycholic acid | 1 | 3 | 223 | 40 | 1IHI_IU5327B- |
| DB01336 | Metocurine | 1 | 3 | 0 | 0 | 3PEO_CU9220F- |
| DB06203 | Alogliptin | 1 | 7 | 0 | 0 | 3G0B_T22800D- |
| DB01011 | Metyrapone | 1 | 47 | 24 | 0 | 1W0G_MYT1499A- |
| DB01030 | Topotecan | 1 | 2 | 0 | 0 | 1K4T_TTC990DA |
| DB00650 | Leucovorin | 1 | 0 | 0 | 0 | 3UWL_FOZ316B- |
| DB01144 | Diclofenamide | 1 | 22 | 48 | 0 | 2POU_I7A1000A- |
| DB01258 | Aliskiren | 1 | 0 | 0 | 0 | 2V0Z_C411327O- |
| DB00320 | Dihydroergotamine | 1 | 4 | 126 | 3 | 4IAQ_2GM2001A- |
| DB00456 | Cefalotin | 1 | 0 | 0 | 0 | 1KVL_CLS371AA |
| DB00373 | Timolol | 1 | 1 | 0 | 0 | 3D4S_TIM401A- |
| DB01179 | Podofilox | 1 | 67 | 0 | 0 | 1SA1_POD700B- |
| DB08916 | Afatinib | 1 | 47 | 0 | 0 | 4G5J_0WM1102A- |
| DB00537 | Ciprofloxacin | 1 | 4 | 519 | 4 | 2XCT_CPF1020X- |
| DB00966 | Telmisartan | 1 | 16 | 211 | 3 | 3VN2_TLS501A- |
| DB08882 | Linagliptin | 1 | 2 | 0 | 0 | 2RGU_356902B- |
| DB00866 | Alprenolol | 1 | 4 | 0 | 0 | 3NYA_JTZ1203A- |
| DB00212 | Remikiren | 1 | 1 | 0 | 0 | 3D91_REM350A- |
| DB01001 | Salbutamol | 1 | 1 | 217 | 40 | 2Y04_68H601A- |
| DB00990 | Exemestane | 1 | 38 | 128 | 66 | 3S7S_EXM601A- |
| DB06228 | Rivaroxaban | 1 | 2 | 0 | 0 | 2W26_RIV1001A- |
| DB01113 | Papaverine | 1 | 16 | 20 | 0 | 3IAK_EV1415A- |
| DB00550 | Propylthiouracil | 1 | 12 | 54 | 0 | 4QYQ_3CJ607C- |
| DB01132 | Pioglitazone | 1 | 5 | 121 | 20 | 2XKW_P1B1478AA |
| DB04786 | Suramin | 1 | 2 | 0 | 0 | 2NYR_SVR401B- |
| DB00276 | Amsacrine | 1 | 2 | 109 | 9 | 4G0U_ASW101F- |
| DB04876 | Vildagliptin | 1 | 12 | 42 | 0 | 3W2T_LF7801A- |
| DB04868 | Nilotinib | 1 | 1 | 354 | 51 | 3CS9_NIL600A- |

^a^ Number of Drugs binding sites with this ligand.

^b^ Number of targets predicted with Z_3_

^c^ Number of Sider side effects for this ligand

^d^ Number of Sider side effects with a frequency > 5%

Table S2 – List of the 400 entries of the Drugs dataset ordered by number of targets

| **PDB** | **Res.** | **Num.** | **Chaîne** | **Alt.** | **Ligand** | **Nb. atoms^a^** | **Targets^b^** | **Nb. Pfams^c^** | **Refs.^d^** |
| --- | --- | --- | --- | --- | --- | --- | --- | --- | --- |
| 1ADC | EOH | 378 | B | - | Ethanol | 3 | 282 | 177 | 73 |
| 4UBP | HAE | 800 | C | - | Acetohydroxamic Acid | 5 | 249 | 182 | 84 |
| 1F8F | EOH | 374 | A | - | Ethanol | 3 | 242 | 168 | 73 |
| 1ADB | EOH | 378 | B | - | Ethanol | 3 | 225 | 153 | 65 |
| 2BU8 | TF4 | 1379 | A | - | Dichloroacetic Acid | 6 | 220 | 148 | 57 |
| 2Q8H | TF4 | 438 | A | - | Dichloroacetic Acid | 6 | 220 | 148 | 58 |
| 1FWE | HAE | 989 | C | - | Acetohydroxamic Acid | 5 | 217 | 173 | 76 |
| 3N8Y | SAL | 900 | B | A | Salicylic acid | 10 | 159 | 107 | 44 |
| 1E9Y | HAE | 800 | B | - | Acetohydroxamic Acid | 5 | 151 | 122 | 56 |
| 1PTH | SAL | 711 | B | - | Salicylic acid | 10 | 148 | 99 | 42 |
| 3NT1 | NPS | 4 | B | - | Naproxen | 17 | 122 | 91 | 29 |
| 4O1Z | MXM | 807 | A | A | Meloxicam | 23 | 110 | 79 | 30 |
| 1XLS | REA | 801 | A | - | Tretinoin | 22 | 105 | 70 | 25 |
| 3UT5 | LOC | 502 | B | - | Colchicine | 29 | 104 | 79 | 28 |
| 1XQL | 4AX | 505 | B | E | Cycloserine | 7 | 102 | 86 | 36 |
| 3N3P | NIZ | 749 | B | - | Isoniazid | 10 | 99 | 77 | 29 |
| 4KWQ | NIZ | 815 | B | - | Isoniazid | 10 | 98 | 77 | 32 |
| 2AYL | FLP | 2701 | B | A | Flurbiprofen | 18 | 97 | 73 | 23 |
| 3RFM | CFF | 330 | A | - | Caffeine | 14 | 93 | 68 | 24 |
| 4PD4 | AOQ | 4003 | C | - | Atovaquone | 26 | 89 | 71 | 23 |
| 4M11 | MXM | 606 | B | A | Meloxicam | 23 | 88 | 67 | 17 |
| 1B2I | AMH | 84 | A | - | Tranexamic Acid | 220 | 88 | 70 | 34 |
| 3FC6 | REA | 501 | C | - | Tretinoin | 22 | 86 | 64 | 22 |
| 3PGH | FLP | 701 | C | - | Flurbiprofen | 18 | 83 | 65 | 21 |
| 1CEB | AMH | 90 | B | - | Tranexamic Acid | 11 | 80 | 68 | 32 |
| 1K74 | 9CR | 463 | A | - | Alitretinoin | 22 | 80 | 54 | 21 |
| 1FM6 | 9CR | 501 | A | - | Alitretinoin | 22 | 79 | 55 | 21 |
| 3OAP | REA | 500 | A | - | Tretinoin | 22 | 77 | 55 | 21 |
| 3RR3 | FLR | 700 | A | - | Flurbiprofen | 18 | 74 | 59 | 20 |
| 4PH9 | IBP | 601 | A | - | Ibuprofen | 15 | 73 | 56 | 20 |
| 4DM8 | REA | 501 | A | - | Tretinoin | 22 | 72 | 50 | 19 |
| 2Q7K | TES | 304 | A | - | Testosterone | 21 | 71 | 54 | 21 |
| 2Q7I | TES | 205 | A | - | Testosterone | 21 | 70 | 55 | 21 |
| 3OLS | EST | 600 | B | - | Estradiol | 20 | 69 | 53 | 20 |
| 1EQG | IBP | 1701 | B | - | Ibuprofen | 15 | 69 | 54 | 18 |
| 3LBD | REA | 424 | A | - | Tretinoin | 22 | 67 | 52 | 16 |
| 1SA1 | POD | 700 | B | - | Podofilox | 30 | 67 | 50 | 11 |
| 1HPK | ACA | 80 | A | - | Aminocaproic Acid | 9 | 64 | 52 | 16 |
| 2WX2 | TPF | 1460 | B | - | Fluconazole | 22 | 64 | 47 | 14 |
| 2AM9 | TES | 1000 | A | - | Testosterone | 21 | 63 | 52 | 18 |
| 3HM1 | J3Z | 2 | A | - | Estrone | 20 | 63 | 48 | 15 |
| 1G5Y | REA | 502 | C | - | Tretinoin | 22 | 63 | 43 | 11 |
| 4O2B | LOC | 503 | B | - | Colchicine | 29 | 63 | 47 | 13 |
| 4IG5 | MMZ | 617 | B | - | Methimazole | 7 | 62 | 54 | 26 |
| 3.00E+22 | LOC | 700 | D | - | Colchicine | 29 | 60 | 45 | 9 |
| 4FN9 | STR | 301 | A | - | Progesterone | 23 | 59 | 47 | 17 |
| 4OTY | LUR | 705 | A | - | Lumiracoxib | 20 | 59 | 46 | 16 |
| 3KHM | TPF | 501 | A | - | Fluconazole | 22 | 59 | 44 | 11 |
| 1A28 | STR | 1 | A | - | Progesterone | 23 | 59 | 48 | 20 |
| 3KIV | ACA | 100 | A | - | Aminocaproic Acid | 9 | 57 | 49 | 25 |
| 2LBD | REA | 500 | A | - | Tretinoin | 22 | 57 | 41 | 14 |
| 2AA5 | STR | 301 | A | - | Progesterone | 23 | 56 | 45 | 21 |
| 3H0A | 9RA | 500 | A | - | Bexarotene | 26 | 55 | 39 | 15 |
| 1YA3 | STR | 3001 | C | - | Progesterone | 23 | 55 | 43 | 15 |
| 3KK6 | CEL | 701 | A | - | Celecoxib | 26 | 54 | 38 | 10 |
| 2OCF | EST | 596 | A | - | Estradiol | 20 | 53 | 41 | 16 |
| 1S9P | DES | 459 | B | - | Diethylstilbestrol | 20 | 52 | 47 | 14 |
| 3FHX | PXL | 313 | B | - | Pyridoxal | 12 | 52 | 42 | 21 |
| 4KA6 | NIZ | 805 | A | - | Isoniazid | 10 | 51 | 35 | 14 |
| 4C2P | X8Z | 709 | A | - | Captopril | 14 | 51 | 40 | 17 |
| 3D90 | NOG | 2001 | B | - | Levonorgestrel | 23 | 50 | 37 | 10 |
| 3ERD | DES | 600 | A | - | Diethylstilbestrol | 20 | 49 | 40 | 13 |
| 1W0G | MYT | 1499 | A | - | Metyrapone | 17 | 47 | 36 | 15 |
| 3N8Y | DIF | 585 | B | B | Diclofenac | 19 | 47 | 38 | 14 |
| 4G5J | 0WM | 1102 | A | - | Afatinib | 34 | 47 | 37 | 14 |
| 2X8Z | X8Z | 1615 | A | - | Captopril | 14 | 46 | 40 | 17 |
| 3TVX | PNX | 902 | B | - | Pentoxifylline | 20 | 46 | 38 | 10 |
| 3S68 | TCW | 227 | A | - | Tolcapone | 20 | 45 | 37 | 19 |
| 2AB2 | SNL | 502 | A | - | Spironolactone | 29 | 45 | 37 | 10 |
| 2OZ7 | CA4 | 1 | A | - | Cyproterone acetate | 29 | 44 | 33 | 7 |
| 2OAX | SNL | 5001 | E | - | Spironolactone | 29 | 44 | 34 | 9 |
| 4NKV | AER | 601 | D | - | Abiraterone | 26 | 44 | 31 | 8 |
| 4LTW | STR | 301 | A | - | Progesterone | 23 | 43 | 33 | 10 |
| 3UUD | EST | 600 | B | - | Estradiol | 20 | 43 | 36 | 10 |
| 1YDB | AZM | 264 | A | - | Acetazolamide | 13 | 43 | 38 | 16 |
| 3CZV | AZM | 263 | A | - | Acetazolamide | 13 | 43 | 33 | 14 |
| 3OLL | EST | 600 | B | - | Estradiol | 20 | 42 | 33 | 10 |
| 1XBB | STI | 1 | A | - | Imatinib | 37 | 42 | 33 | 8 |
| 3VHU | SNL | 1001 | A | - | Spironolactone | 29 | 41 | 32 | 10 |
| 1J37 | X8Z | 801 | A | - | Captopril | 14 | 41 | 33 | 18 |
| 3W6H | AZM | 303 | A | - | Acetazolamide | 13 | 41 | 35 | 9 |
| 4DAJ | 0HK | 2000 | C | - | Tiotropium | 26 | 41 | 33 | 12 |
| 1AX9 | EDR | 999 | A | - | Edrophonium | 12 | 40 | 35 | 15 |
| 1MX1 | THA | 2 | B | A | Tacrine | 15 | 40 | 31 | 11 |
| 3M7R | VDX | 425 | A | - | Calcitriol | 30 | 39 | 29 | 11 |
| 4R1Z | AER | 601 | A | - | Abiraterone | 26 | 38 | 30 | 4 |
| 3S7S | EXM | 601 | A | - | Exemestane | 22 | 38 | 30 | 10 |
| 2PK4 | ACA | 100 | A | - | Aminocaproic Acid | 9 | 38 | 34 | 17 |
| 4J24 | EST | 600 | C | - | Estradiol | 20 | 36 | 33 | 6 |
| 1ZSB | AZM | 264 | A | - | Acetazolamide | 13 | 36 | 34 | 15 |
| 2WUZ | TPF | 1460 | B | - | Fluconazole | 22 | 36 | 27 | 12 |
| 2ACK | EDR | 999 | A | - | Edrophonium | 12 | 36 | 32 | 7 |
| 2KI5 | AC2 | 1 | A | A | Aciclovir | 5 | 35 | 32 | 15 |
| 4NKF | 210 | 404 | A | - | Pamidronate | 13 | 35 | 29 | 10 |
| 2YJA | EST | 1550 | B | - | Estradiol | 20 | 34 | 32 | 11 |
| 3TZF | 08D | 280 | B | - | Sulfamethoxazole | 17 | 33 | 29 | 13 |
| 3RUK | AER | 601 | D | - | Abiraterone | 26 | 33 | 25 | 9 |
| 4OK1 | 198 | 1001 | A | - | Bicalutamide | 29 | 33 | 25 | 6 |
| 2C72 | RSA | 601 | A | - | Rasagiline | 13 | 33 | 29 | 13 |
| 2W8Y | 486 | 1000 | A | - | Mifepristone | 32 | 33 | 25 | 8 |
| 4MS4 | 2C0 | 501 | A | - | Baclofen | 14 | 33 | 29 | 9 |
| 3CLD | GW6 | 1 | A | - | Fluticasone furoate | 37 | 32 | 25 | 9 |
| 1PK2 | ACA | 91 | A | - | Aminocaproic Acid | 9 | 32 | 29 | 9 |
| 2AA6 | STR | 401 | A | - | Progesterone | 23 | 32 | 28 | 9 |
| 1JD0 | AZM | 1400 | A | - | Acetazolamide | 13 | 31 | 29 | 13 |
| 1ITU | CIL | 451 | A | - | Cilastatin | 16 | 31 | 30 | 8 |
| 1Z95 | 198 | 501 | A | - | Bicalutamide | 29 | 31 | 22 | 3 |
| 1XOZ | CIA | 501 | A | - | Tadalafil | 29 | 31 | 23 | 5 |
| 3H52 | 486 | 4 | C | - | Mifepristone | 32 | 30 | 22 | 5 |
| 3L4W | MIG | 1001 | A | - | Miglitol | 14 | 30 | 29 | 15 |
| 1CEA | ACA | 90 | A | - | Aminocaproic Acid | 9 | 29 | 26 | 12 |
| 1YHM | AHD | 901 | A | - | Alendronate | 14 | 29 | 21 | 7 |
| 2E91 | ZOL | 901 | A | - | Zoledronate | 16 | 29 | 27 | 7 |
| 3G9E | RO7 | 1 | A | - | Aleglitazar | 31 | 28 | 25 | 7 |
| 1GS4 | ZK5 | 1918 | A | - | Fludrocortisone | 27 | 28 | 24 | 5 |
| 3C0Z | SHH | 301 | B | - | Vorinostat | 9 | 27 | 26 | 6 |
| 4JUO | LFX | 101 | F | - | Levofloxacin | 26 | 27 | 16 | 6 |
| 2C73 | RSA | 601 | A | - | Rasagiline | 13 | 27 | 26 | 9 |
| 1XZX | T3 | 500 | X | - | Liothyronine | 23 | 27 | 22 | 5 |
| 2YDO | ADN | 400 | A | - | Adenosine | 19 | 25 | 22 | 6 |
| 1BZM | MZM | 262 | A | - | Methazolamide | 14 | 24 | 21 | 6 |
| 2C6N | LPR | 705 | B | - | Lisinopril | 29 | 24 | 22 | 7 |
| 3DC3 | AZM | 263 | A | - | Acetazolamide | 13 | 24 | 23 | 8 |
| 1QZR | CDX | 901 | B | A | Dexrazoxane | 19 | 24 | 19 | 6 |
| 3V2M | AZM | 303 | A | - | Acetazolamide | 13 | 23 | 22 | 6 |
| 3Q95 | ESL | 700 | B | - | Estriol | 21 | 23 | 21 | 7 |
| 2F89 | 210 | 9001 | F | - | Pamidronate | 13 | 22 | 18 | 4 |
| 1AZM | AZM | 262 | A | - | Acetazolamide | 13 | 22 | 18 | 8 |
| 2POU | I7A | 1000 | A | - | Diclofenamide | 16 | 22 | 19 | 9 |
| 1ACJ | THA | 999 | A | - | Tacrine | 15 | 22 | 21 | 6 |
| 4U14 | 0HK | 2000 | A | - | Tiotropium | 26 | 22 | 18 | 8 |
| 3BJM | BJM | 2 | B | - | Saxagliptin | 23 | 22 | 20 | 10 |
| 1I00 | D16 | 409 | B | - | Raltitrexed | 32 | 21 | 17 | 5 |
| 3FOF | MFX | 0 | F | - | Moxifloxacin | 29 | 21 | 19 | 6 |
| 4LNX | T44 | 501 | A | - | Dextrothyroxine | 24 | 21 | 12 | 3 |
| 3CLB | TMQ | 611 | A | - | Trimetrexate | 27 | 20 | 15 | 3 |
| 4U5J | RXT | 601 | B | - | Ruxolitinib | 23 | 20 | 19 | 6 |
| 3HS6 | EPA | 1 | B | - | Icosapent | 22 | 20 | 15 | 7 |
| 1M2Z | DEX | 301 | A | - | Betamethasone | 28 | 20 | 18 | 6 |
| 3LXE | TOR | 262 | A | - | Topiramate | 22 | 20 | 18 | 7 |
| 4AGD | B49 | 2000 | A | - | Sunitinib | 29 | 20 | 19 | 6 |
| 1YDA | AZM | 264 | A | - | Acetazolamide | 13 | 19 | 19 | 6 |
| 4KFA | ZOL | 404 | A | - | Zoledronate | 16 | 19 | 16 | 3 |
| 3B6R | CRN | 603 | B | - | Creatine | 9 | 18 | 16 | 8 |
| 1XP0 | VDN | 201 | A | - | Vardenafil | 34 | 18 | 14 | 4 |
| 4LTW | 486 | 303 | A | - | Mifepristone | 32 | 18 | 17 | 5 |
| 1UDU | CIA | 1003 | A | - | Tadalafil | 29 | 18 | 15 | 3 |
| 2C75 | RSA | 601 | A | - | Rasagiline | 13 | 18 | 17 | 5 |
| 1Y0X | T44 | 500 | X | - | Dextrothyroxine | 24 | 17 | 12 | 6 |
| 4LNX | T3 | 502 | A | - | Liothyronine | 23 | 17 | 12 | 5 |
| 3JUS | ECN | 602 | A | A | Econazole | 24 | 16 | 10 | 6 |
| 1W6R | GNT | 1536 | A | - | Galantamine | 21 | 16 | 15 | 5 |
| 1A4L | DCF | 1353 | C | - | Pentostatin | 19 | 16 | 13 | 5 |
| 1DB1 | VDX | 428 | A | - | Calcitriol | 30 | 16 | 16 | 6 |
| 4LS7 | 1X9 | 504 | A | - | Cerulenin | 16 | 16 | 16 | 4 |
| 2H79 | T3 | 1 | A | - | Liothyronine | 23 | 16 | 14 | 4 |
| 1NHZ | 486 | 800 | A | - | Mifepristone | 32 | 16 | 15 | 5 |
| 3G5D | 1N1 | 1 | A | - | Dasatinib | 33 | 16 | 14 | 3 |
| 3VN2 | TLS | 501 | A | - | Telmisartan | 39 | 16 | 15 | 7 |
| 1DX6 | GNT | 1537 | A | - | Galantamine | 21 | 16 | 14 | 4 |
| 3IAK | EV1 | 415 | A | - | Papaverine | 25 | 16 | 12 | 3 |
| 2F9K | ZOL | 9001 | F | - | Zoledronate | 16 | 16 | 14 | 3 |
| 3V2J | AZM | 302 | A | - | Acetazolamide | 13 | 16 | 16 | 8 |
| 3HS4 | AZM | 701 | A | - | Acetazolamide | 13 | 16 | 15 | 8 |
| 3HKU | TOR | 300 | A | - | Topiramate | 22 | 15 | 14 | 5 |
| 3DAZ | MZM | 263 | A | - | Methazolamide | 14 | 15 | 12 | 5 |
| 1IE9 | VDX | 500 | A | - | Calcitriol | 30 | 15 | 14 | 4 |
| 1AJ6 | NOV | 1 | A | - | Novobiocin | 44 | 15 | 12 | 2 |
| 4PYL | TCW | 303 | A | - | Tolcapone | 20 | 15 | 13 | 6 |
| 3ML5 | AZM | 264 | A | - | Acetazolamide | 13 | 15 | 15 | 8 |
| 4EY6 | GNT | 604 | A | - | Galantamine | 21 | 15 | 13 | 3 |
| 3LD6 | KKK | 602 | B | - | Ketoconazole | 36 | 15 | 15 | 4 |
| 3JUS | ECL | 600 | B | B | Econazole | 24 | 14 | 11 | 2 |
| 3G8I | RO7 | 1 | A | - | Aleglitazar | 31 | 14 | 13 | 4 |
| 3MZE | CFX | 364 | A | - | Cefoxitin | 24 | 14 | 13 | 6 |
| 1RJ6 | AZM | 400 | A | - | Acetazolamide | 13 | 13 | 13 | 6 |
| 2F92 | AHD | 9001 | F | - | Alendronate | 14 | 13 | 11 | 5 |
| 1ERR | RAL | 600 | A | - | Raloxifene | 34 | 13 | 13 | 3 |
| 1KI2 | GA2 | 1 | A | - | Ganciclovir | 18 | 13 | 12 | 6 |
| 4KQU | AHD | 405 | A | - | Alendronate | 14 | 13 | 12 | 4 |
| 3N45 | ZOL | 354 | F | - | Zoledronate | 16 | 13 | 11 | 4 |
| 4CPN | ZMR | 700 | A | - | Zanamivir | 23 | 13 | 10 | 3 |
| 1VM1 | TAZ | 504 | A | - | Tazobactam | 20 | 13 | 13 | 5 |
| 4QYQ | 3CJ | 607 | C | - | Propylthiouracil | 11 | 12 | 12 | 3 |
| 4MXY | DB8 | 601 | A | - | Bosutinib | 36 | 12 | 10 | 3 |
| 1QKN | RAL | 600 | A | - | Raloxifene | 34 | 12 | 11 | 1 |
| 3QLG | 1N1 | 601 | A | A | Dasatinib | 8 | 12 | 10 | 2 |
| 4AFG | QMR | 1214 | A | - | Varenicline | 16 | 12 | 11 | 4 |
| 3ZNC | BZ1 | 500 | A | - | Brinzolamide | 23 | 12 | 12 | 3 |
| 1BSX | T3 | 1 | A | - | Liothyronine | 23 | 12 | 11 | 4 |
| 3W2T | LF7 | 801 | A | - | Vildagliptin | 22 | 12 | 11 | 5 |
| 4CPZ | ZMR | 1470 | C | - | Zanamivir | 23 | 11 | 11 | 3 |
| 4D1O | H4B | 1481 | B | - | Tetrahydrobiopterin | 17 | 11 | 10 | 2 |
| 4KPJ | 210 | 901 | A | - | Pamidronate | 13 | 11 | 11 | 3 |
| 2XP2 | VGH | 9000 | A | - | Crizotinib | 30 | 11 | 9 | 4 |
| 3KU9 | SPM | 700 | A | - | Spermine | 14 | 11 | 7 | 1 |
| 3UE4 | DB8 | 601 | A | - | Bosutinib | 36 | 11 | 10 | 3 |
| 4D1P | H4B | 600 | B | - | Tetrahydrobiopterin | 17 | 11 | 11 | 2 |
| 2ZVA | 1N1 | 513 | A | - | Dasatinib | 33 | 10 | 9 | 1 |
| 3RZE | D7V | 1201 | A | B | Doxepin | 21 | 10 | 10 | 3 |
| 4I00 | ZMR | 509 | A | - | Zanamivir | 23 | 10 | 8 | 3 |
| 4OJB | 198 | 1001 | A | - | Bicalutamide | 29 | 10 | 6 | 0 |
| 1C3S | SHH | 952 | A | - | Vorinostat | 19 | 10 | 9 | 3 |
| 4MM7 | 29J | 603 | A | - | Desvenlafaxine | 19 | 10 | 9 | 1 |
| 3N8X | NIM | 701 | A | - | Nimesulide | 21 | 10 | 10 | 3 |
| 4U15 | 0HK | 2001 | A | - | Tiotropium | 26 | 10 | 9 | 2 |
| 3CAJ | EZL | 265 | A | - | Ethoxzolamide | 16 | 9 | 8 | 3 |
| 1A4G | ZMR | 466 | B | - | Zanamivir | 23 | 9 | 9 | 3 |
| 2W4X | STZ | 1591 | A | - | Streptozocin | 18 | 9 | 7 | 3 |
| 4K0Z | MZM | 308 | A | - | Methazolamide | 14 | 9 | 9 | 2 |
| 3ZBF | VGH | 3000 | A | - | Crizotinib | 30 | 8 | 7 | 1 |
| 2CML | ZMR | 4477 | D | - | Zanamivir | 23 | 8 | 8 | 2 |
| 4ANQ | VGH | 9000 | A | - | Crizotinib | 30 | 8 | 6 | 0 |
| 4M2U | ETS | 302 | A | - | Dorzolamide | 19 | 8 | 8 | 4 |
| 4WKQ | IRE | 1101 | A | - | Gefitinib | 31 | 8 | 7 | 0 |
| 1CIL | ETS | 263 | A | - | Dorzolamide | 19 | 8 | 7 | 4 |
| 3DCW | EZL | 301 | A | - | Ethoxzolamide | 16 | 8 | 8 | 2 |
| 1PXX | DIF | 701 | A | - | Diclofenac | 19 | 8 | 6 | 3 |
| 3DCS | MZM | 263 | A | - | Methazolamide | 14 | 7 | 7 | 2 |
| 3MNE | DEX | 784 | A | - | Betamethasone | 28 | 7 | 7 | 3 |
| 3LN1 | CEL | 682 | A | - | Celecoxib | 26 | 7 | 7 | 3 |
| 1IA0 | TXL | 502 | B | - | Docetaxel | 58 | 7 | 7 | 4 |
| 4K13 | ETS | 304 | A | - | Dorzolamide | 19 | 7 | 7 | 3 |
| 3B2R | VDN | 1 | A | - | Vardenafil | 34 | 7 | 7 | 2 |
| 3G0B | T22 | 800 | D | - | Alogliptin | 25 | 7 | 6 | 1 |
| 2ITY | IRE | 2020 | A | - | Gefitinib | 31 | 6 | 4 | 0 |
| 1TD2 | PXL | 288 | A | - | Pyridoxal | 12 | 6 | 6 | 2 |
| 2XKK | MFX | 1100 | E | - | Moxifloxacin | 29 | 6 | 6 | 2 |
| 4LU3 | AZM | 302 | A | - | Acetazolamide | 13 | 6 | 6 | 2 |
| 4QA2 | SHH | 404 | A | - | Vorinostat | 19 | 6 | 5 | 1 |
| 3MDZ | EZL | 264 | A | - | Ethoxzolamide | 16 | 6 | 6 | 4 |
| 1TBF | VIA | 501 | A | - | Sildenafil | 33 | 6 | 6 | 1 |
| 4KQS | RIS | 405 | A | - | Risedronate | 17 | 6 | 5 | 2 |
| 4KPD | RIS | 405 | A | - | Risedronate | 17 | 6 | 4 | 2 |
| 1FKF | FK5 | 108 | A | - | Tacrolimus | 57 | 6 | 6 | 3 |
| 3NOS | H4B | 511 | A | - | Tetrahydrobiopterin | 17 | 6 | 6 | 2 |
| 2QIS | RIS | 901 | A | - | Risedronate | 17 | 6 | 4 | 1 |
| 1JU6 | LYA | 315 | B | - | Pemetrexed | 31 | 5 | 4 | 1 |
| 2XKW | P1B | 1478 | A | A | Pioglitazone | 25 | 5 | 5 | 1 |
| 2X91 | LPR | 1615 | A | - | Lisinopril | 29 | 5 | 5 | 2 |
| 3DD0 | EZL | 301 | A | - | Ethoxzolamide | 16 | 5 | 5 | 3 |
| 1KI3 | PE2 | 1 | A | - | Penciclovir | 18 | 5 | 5 | 1 |
| 3G0F | B49 | 9001 | A | - | Sunitinib | 22 | 5 | 5 | 2 |
| 3JWQ | VIA | 901 | D | - | Sildenafil | 33 | 5 | 5 | 1 |
| 4LNW | T3 | 501 | A | - | Liothyronine | 23 | 5 | 4 | 1 |
| 3TIC | ZMR | 1002 | C | - | Zanamivir | 23 | 5 | 5 | 2 |
| 2YFX | VGH | 9000 | A | - | Crizotinib | 30 | 5 | 4 | 0 |
| 1MMK | H4B | 1427 | A | - | Tetrahydrobiopterin | 17 | 5 | 5 | 2 |
| 2QXS | RAL | 600 | A | - | Raloxifene | 34 | 5 | 5 | 0 |
| 3GWS | T3 | 500 | X | - | Liothyronine | 23 | 5 | 5 | 1 |
| 1T46 | STI | 3 | A | - | Imatinib | 37 | 5 | 4 | 1 |
| 1KW0 | H4B | 429 | A | - | Tetrahydrobiopterin | 17 | 5 | 5 | 0 |
| 4QA0 | SHH | 404 | A | - | Vorinostat | 19 | 5 | 4 | 1 |
| 1FM6 | BRL | 504 | X | - | Rosiglitazone | 25 | 5 | 5 | 2 |
| 4BDS | THA | 701 | A | - | Tacrine | 15 | 5 | 5 | 2 |
| 4IAQ | 2GM | 2001 | A | - | Dihydroergotamine | 43 | 4 | 4 | 1 |
| 1ZGY | BRL | 503 | A | - | Rosiglitazone | 25 | 4 | 4 | 0 |
| 4MM6 | 29E | 603 | A | - | Duloxetine | 21 | 4 | 4 | 0 |
| 2XCT | CPF | 1020 | X | - | Ciprofloxacin | 24 | 4 | 4 | 0 |
| 1ZZ1 | SHH | 2452 | A | - | Vorinostat | 19 | 4 | 4 | 1 |
| 3NYA | JTZ | 1203 | A | - | Alprenolol | 18 | 4 | 3 | 1 |
| 1TCO | FK5 | 509 | C | - | Tacrolimus | 57 | 4 | 4 | 1 |
| 1O86 | LPR | 702 | A | - | Lisinopril | 29 | 4 | 4 | 1 |
| 1IG3 | VIB | 502 | A | - | Thiamine | 18 | 4 | 4 | 2 |
| 4ASD | BAX | 1500 | A | - | Sorafenib | 32 | 4 | 4 | 2 |
| 4IAR | ERM | 2001 | A | - | Ergotamine | 43 | 4 | 4 | 2 |
| 4P6X | HCY | 900 | I | - | Hydrocortisone | 26 | 4 | 4 | 2 |
| 3PMZ | TUB | 220 | E | - | Tubocurarine | 45 | 4 | 4 | 2 |
| 1IGX | EPA | 700 | A | - | Icosapent | 22 | 4 | 4 | 1 |
| 1FE2 | LAX | 700 | A | - | Dihomo-Î³-linolenic acid | 22 | 4 | 4 | 1 |
| 3G0E | B49 | 9000 | A | - | Sunitinib | 22 | 4 | 4 | 1 |
| 2JFA | RAL | 600 | A | - | Raloxifene | 34 | 4 | 4 | 0 |
| 3SFE | TMG | 1 | C | - | Thiabendazole | 14 | 3 | 3 | 0 |
| 4FFW | 715 | 801 | B | - | Sitagliptin | 28 | 3 | 3 | 0 |
| 3J6G | TA1 | 502 | B | - | Paclitaxel | 62 | 3 | 3 | 1 |
| 4COX | IMN | 701 | B | - | Indomethacin | 25 | 3 | 3 | 0 |
| 1TUB | TXL | 501 | B | - | Docetaxel | 58 | 3 | 3 | 0 |
| 4LDO | ALE | 1402 | A | - | Epinephrine | 13 | 3 | 3 | 1 |
| 4LXZ | SHH | 408 | B | - | Vorinostat | 19 | 3 | 3 | 0 |
| 3K9F | LFX | 0 | H | - | Levofloxacin | 26 | 3 | 3 | 0 |
| 2FKE | FK5 | 108 | A | - | Tacrolimus | 57 | 3 | 3 | 0 |
| 4M2V | BZ1 | 302 | A | - | Brinzolamide | 23 | 3 | 3 | 0 |
| 1IHI | IU5 | 327 | B | - | Ursodeoxycholic acid | 28 | 3 | 2 | 0 |
| 3PEO | CU9 | 220 | F | - | Metocurine | 48 | 3 | 3 | 1 |
| 1UDT | VIA | 1000 | A | - | Sildenafil | 33 | 3 | 3 | 1 |
| 1XKK | FMM | 91 | A | - | Lapatinib | 40 | 3 | 3 | 2 |
| 2DG4 | RAP | 501 | A | - | Sirolimus | 65 | 3 | 3 | 2 |
| 4M48 | 21B | 704 | A | - | Nortriptyline | 20 | 3 | 3 | 1 |
| 3VW7 | VPX | 2001 | A | - | Vorapaxar | 36 | 3 | 3 | 2 |
| 1JFF | TA1 | 601 | B | - | Paclitaxel | 62 | 3 | 3 | 1 |
| 4EB4 | D16 | 402 | A | - | Raltitrexed | 32 | 3 | 3 | 1 |
| 2NYR | SVR | 401 | B | - | Suramin | 86 | 2 | 2 | 1 |
| 4HFP | 15U | 402 | B | - | Argatroban | 35 | 2 | 2 | 1 |
| 1X70 | 715 | 801 | B | - | Sitagliptin | 28 | 2 | 2 | 0 |
| 2Y03 | 5FW | 601 | A | - | Isoprenaline | 15 | 2 | 2 | 1 |
| 4M2X | TMQ | 202 | C | - | Trimetrexate | 27 | 2 | 2 | 1 |
| 1FAP | RAP | 108 | A | - | Sirolimus | 65 | 2 | 2 | 1 |
| 1FKB | RAP | 108 | A | - | Sirolimus | 65 | 2 | 2 | 2 |
| 2GQG | 1N1 | 501 | A | - | Dasatinib | 33 | 2 | 2 | 1 |
| 1EVE | E20 | 2001 | A | - | Donepezil | 28 | 2 | 2 | 1 |
| 3C6M | SPM | 501 | C | - | Spermine | 14 | 2 | 2 | 1 |
| 2HXF | TA1 | 601 | B | - | Paclitaxel | 62 | 2 | 2 | 0 |
| 3UM5 | CP6 | 709 | B | - | Pyrimethamine | 17 | 2 | 2 | 1 |
| 4G0U | ASW | 101 | F | - | Amsacrine | 28 | 2 | 2 | 1 |
| 1K4T | TTC | 990 | D | A | Topotecan | 31 | 2 | 2 | 0 |
| 1TD7 | NFL | 2001 | A | - | Niflumic Acid | 20 | 2 | 2 | 0 |
| 4DRI | RAP | 201 | A | - | Sirolimus | 65 | 2 | 2 | 2 |
| 2W26 | RIV | 1001 | A | - | Rivaroxaban | 29 | 2 | 2 | 1 |
| 2VDM | AGG | 1462 | B | - | Tirofiban | 30 | 2 | 2 | 0 |
| 2QMJ | ACR | 1001 | A | - | Acarbose | 44 | 2 | 2 | 1 |
| 2RGU | 356 | 902 | B | - | Linagliptin | 35 | 2 | 2 | 1 |
| 4EY7 | E20 | 604 | A | - | Donepezil | 28 | 2 | 2 | 0 |
| 1DWC | MIT | 1 | H | - | Argatroban | 35 | 2 | 2 | 1 |
| 1P93 | DEX | 4999 | D | - | Betamethasone | 28 | 2 | 2 | 0 |
| 3GWX | EPA | 3 | B | A | Icosapent | 22 | 2 | 2 | 0 |
| 2WGJ | VGH | 2346 | A | - | Crizotinib | 30 | 1 | 1 | 1 |
| 1ETR | MIT | 1 | H | - | Argatroban | 35 | 1 | 1 | 0 |
| 3K2H | LYA | 513 | A | - | Pemetrexed | 31 | 1 | 1 | 0 |
| 2BLA | CP6 | 1240 | A | - | Pyrimethamine | 17 | 1 | 1 | 0 |
| 1JUJ | LYA | 315 | B | - | Pemetrexed | 31 | 1 | 1 | 0 |
| 2Y6O | 1N1 | 1892 | A | - | Dasatinib | 33 | 1 | 1 | 0 |
| 1QYX | ASD | 500 | A | - | 4-Androstenedione | 21 | 1 | 1 | 0 |
| 1HVY | D16 | 414 | A | - | Raltitrexed | 32 | 1 | 1 | 0 |
| 4MXO | DB8 | 601 | A | - | Bosutinib | 36 | 1 | 1 | 0 |
| 2F94 | BFQ | 9001 | F | A | Ibandronate | 19 | 1 | 1 | 0 |
| 1J8U | H4B | 429 | A | - | Tetrahydrobiopterin | 17 | 1 | 1 | 0 |
| 2P16 | GG2 | 298 | A | - | Apixaban | 34 | 1 | 1 | 1 |
| 3W37 | ACR | 1001 | A | - | Acarbose | 44 | 1 | 1 | 0 |
| 1HZE | RBF | 99 | B | - | Riboflavin | 27 | 1 | 1 | 0 |
| 3UG2 | IRE | 1 | A | - | Gefitinib | 31 | 1 | 1 | 0 |
| 3OG7 | 32 | 1 | A | - | Vemurafenib | 33 | 1 | 1 | 0 |
| 3CS9 | NIL | 600 | A | - | Nilotinib | 39 | 1 | 1 | 1 |
| 4KM0 | CP6 | 201 | B | - | Pyrimethamine | 17 | 1 | 1 | 0 |
| 1KXH | ACR | 598 | A | - | Acarbose | 44 | 1 | 1 | 1 |
| 3D91 | REM | 350 | A | - | Remikiren | 44 | 1 | 1 | 0 |
| 2XYT | TC9 | 1206 | B | - | Tubocurarine | 45 | 1 | 1 | 1 |
| 2Y04 | 68H | 601 | A | - | Salbutamol | 17 | 1 | 1 | 0 |
| 1M17 | AQ4 | 999 | A | - | Erlotinib | 29 | 1 | 1 | 1 |
| 3EIG | MTX | 200 | A | - | Methotrexate | 33 | 1 | 1 | 0 |
| 4E0F | RBF | 301 | A | - | Riboflavin | 27 | 1 | 1 | 0 |
| 1YNN | RFP | 1120 | C | - | Rifampicin | 59 | 1 | 1 | 0 |
| 4M2R | BZ1 | 302 | A | - | Brinzolamide | 23 | 1 | 1 | 0 |
| 1AJ0 | SAN | 561 | A | - | Sulfanilamide | 11 | 1 | 1 | 0 |
| 4DRH | RAP | 201 | A | - | Sirolimus | 65 | 1 | 1 | 0 |
| 3D4S | TIM | 401 | A | - | Timolol | 21 | 1 | 1 | 0 |
| 1UWH | BAX | 1723 | B | - | Sorafenib | 32 | 1 | 1 | 1 |
| 4HJO | AQ4 | 1001 | A | - | Erlotinib | 29 | 1 | 1 | 0 |
| 3BBT | FMM | 91 | B | - | Lapatinib | 27 | 1 | 1 | 0 |
| 4UXQ | 0LI | 1752 | A | - | Ponatinib | 39 | 1 | 1 | 0 |
| 1J3J | CP6 | 709 | B | - | Pyrimethamine | 17 | 0 | 0 | 0 |
| 1DLS | MTX | 188 | A | - | Methotrexate | 33 | 0 | 0 | 0 |
| 4KMU | RFP | 1401 | C | - | Rifampicin | 59 | 0 | 0 | 0 |
| 3A3Y | OBN | 6000 | A | - | Ouabain | 41 | 0 | 0 | 0 |
| 4AF0 | MOA | 1526 | B | - | Mycophenolic acid | 23 | 0 | 0 | 0 |
| 1JR1 | MOA | 1332 | A | - | Mycophenolic acid | 23 | 0 | 0 | 0 |
| 2V0Z | C41 | 1327 | O | - | Aliskiren | 39 | 0 | 0 | 0 |
| 1UWJ | BAX | 1723 | B | - | Sorafenib | 32 | 0 | 0 | 0 |
| 2F16 | BO2 | 1402 | K | - | Bortezomib | 28 | 0 | 0 | 0 |
| 4NC3 | ERM | 1202 | A | - | Ergotamine | 43 | 0 | 0 | 0 |
| 3MG0 | BO2 | 1402 | K | - | Bortezomib | 28 | 0 | 0 | 0 |
| 3TOP | ACR | 1 | B | - | Acarbose | 44 | 0 | 0 | 0 |
| 4MBS | MRV | 1101 | B | - | Maraviroc | 37 | 0 | 0 | 0 |
| 4K0F | 1YN | 602 | A | - | Itraconazole | 49 | 0 | 0 | 0 |
| 3QFX | CP6 | 602 | A | - | Pyrimethamine | 17 | 0 | 0 | 0 |
| 3FO7 | IMN | 301 | A | - | Indomethacin | 25 | 0 | 0 | 0 |
| 1U72 | MTX | 188 | A | - | Methotrexate | 33 | 0 | 0 | 0 |
| 4IB4 | ERM | 2001 | A | - | Ergotamine | 43 | 0 | 0 | 0 |
| 2A69 | RPT | 8001 | C | - | Rifapentine | 63 | 0 | 0 | 0 |
| 1HWK | 117 | 3 | D | - | Atorvastatin | 41 | 0 | 0 | 0 |
| 3N23 | OBN | 1 | C | - | Ouabain | 41 | 0 | 0 | 0 |
| 3UWL | FOZ | 316 | B | - | Leucovorin | 34 | 0 | 0 | 0 |
| 1MRL | DOL | 302 | C | - | Dalfopristin | 48 | 0 | 0 | 0 |
| 2OTH | IMN | 301 | A | - | Indomethacin | 25 | 0 | 0 | 0 |
| 4DRJ | RAP | 201 | A | - | Sirolimus | 65 | 0 | 0 | 0 |
| 4EB6 | VLB | 503 | C | - | Vinblastine | 59 | 0 | 0 | 0 |
| 2Y00 | Y00 | 601 | B | - | Dobutamine | 22 | 0 | 0 | 0 |
| 4URN | NOV | 2000 | A | - | Novobiocin | 44 | 0 | 0 | 0 |
| 1I6V | RFP | 1640 | C | - | Rifampicin | 59 | 0 | 0 | 0 |
| 1NB9 | RBF | 401 | A | - | Riboflavin | 27 | 0 | 0 | 0 |
| 3DZY | BRL | 478 | D | - | Rosiglitazone | 25 | 0 | 0 | 0 |
| 3HEG | BAX | 1 | A | - | Sorafenib | 32 | 0 | 0 | 0 |
| 3IK3 | 0LI | 1 | A | - | Ponatinib | 39 | 0 | 0 | 0 |
| 3QX3 | EVP | 1 | D | - | Etoposide | 42 | 0 | 0 | 0 |
| 1Z2B | VLB | 800 | C | - | Vinblastine | 59 | 0 | 0 | 0 |
| 2Y01 | Y00 | 601 | B | - | Dobutamine | 22 | 0 | 0 | 0 |
| 3GCS | BAX | 401 | A | - | Sorafenib | 32 | 0 | 0 | 0 |
| 4AMJ | CVD | 1359 | A | - | Carvedilol | 30 | 0 | 0 | 0 |
| 1KVL | CLS | 371 | A | A | Cefalotin | 26 | 0 | 0 | 0 |
| 2A68 | RBT | 8001 | C | - | Rifabutin | 61 | 0 | 0 | 0 |
| 3OXZ | 0LI | 1 | A | - | Ponatinib | 39 | 0 | 0 | 0 |
| 4G0V | MIX | 1301 | A | - | Mitoxantrone | 32 | 0 | 0 | 0 |
| 4KOE | TR6 | 101 | F | - | Trovafloxacin | 30 | 0 | 0 | 0 |
| 1T6Z | RBF | 596 | B | - | Riboflavin | 27 | 0 | 0 | 0 |
| 4URO | NOV | 2000 | A | - | Novobiocin | 44 | 0 | 0 | 0 |
| 1PKV | RBF | 101 | B | - | Riboflavin | 27 | 0 | 0 | 0 |
| 1HW8 | 114 | 2 | A | - | Mevastatin | 29 | 0 | 0 | 0 |
| 2PL0 | STI | 200 | A | - | Imatinib | 37 | 0 | 0 | 0 |
| 4HYT | OBN | 2004 | C | - | Ouabain | 41 | 0 | 0 | 0 |
| 1UHO | VDN | 1000 | A | - | Vardenafil | 34 | 0 | 0 | 0 |
| 4FO4 | MOA | 502 | B | - | Mycophenolic acid | 23 | 0 | 0 | 0 |

^a^ Number of ligand heavy atoms

^b^ Number of predicted targets with Z_3_

^c^ Number of Pfam families represented by the predicted targets with Z_3_

^d^ Number of references containing a special keyword in the title

Table S3 – Targets predicted multiple times for the same drug

| **Ligand** | **Entries** | **Target** | **3.0^a^** | **2.5^b^** | **2.0^c^** | **Organism** | **Target name** | **Drugs entries (Z_3_)** |
| --- | --- | --- | --- | --- | --- | --- | --- | --- |
| Acetazolamide | 14 | 3KS3_2 | 8 | 10 | 13 | Homo sapiens | CARBONIC ANHYDRASE 2 | 3V2M_AZM303A- 3HS4_AZM701A- 3DC3_AZM263A- 1YDA_AZM264A- 1ZSB_AZM264A- 3V2J_AZM302A- 3ML5_AZM264A- 4LU3_AZM302A- |
| Acetazolamide | 14 | 1TT8_6 | 8 | 11 | 13 | Escherichia coli | CHORISMATE-PYRUVATE LYASE | 1RJ6_AZM400A- 3W6H_AZM303A- 1ZSB_AZM264A- 1JD0_AZM1400A- 3DC3_AZM263A- 3HS4_AZM701A- 3CZV_AZM263A- 1AZM_AZM262A- |
| Acetazolamide | 14 | 3WKX_1 | 7 | 10 | 12 | Bifidobacterium longum | NON-REDUCING END BETA-L-ARABINOFURANOSIDASE | 3V2M_AZM303A- 3HS4_AZM701A- 1YDA_AZM264A- 1ZSB_AZM264A- 1YDB_AZM264A- 3V2J_AZM302A- 3ML5_AZM264A- |
| Acetazolamide | 14 | 4PVK_1 | 7 | 11 | 13 | Phleum pratense | POLLEN ALLERGEN PHL P 4.0202 | 3V2M_AZM303A- 1JD0_AZM1400A- 3HS4_AZM701A- 3DC3_AZM263A- 1RJ6_AZM400A- 1YDA_AZM264A- 3ML5_AZM264A- |
| Acetazolamide | 14 | 1W0N_1 | 7 | 11 | 13 | Paenibacillus polymyxa | ENDO-1,4-BETA-XYLANASE D | 3V2M_AZM303A- 1JD0_AZM1400A- 3HS4_AZM701A- 1YDA_AZM264A- 1ZSB_AZM264A- 1YDB_AZM264A- 4LU3_AZM302A- |
| Acetazolamide | 14 | 3EF8_3 | 6 | 11 | 13 | Novosphingobium aromaticivorans | PUTATIVE SCYALONE DEHYDRATASE | 3V2M_AZM303A- 3DC3_AZM263A- 1YDA_AZM264A- 1ZSB_AZM264A- 3V2J_AZM302A- 3ML5_AZM264A- |
| Acetazolamide | 14 | 1FMJ_2 | 5 | 12 | 13 | Spodoptera frugiperda | RETINOL DEHYDRATASE | 1JD0_AZM1400A- 3HS4_AZM701A- 3DC3_AZM263A- 3W6H_AZM303A- 1ZSB_AZM264A- |
| Acetazolamide | 14 | 1MXR_1 | 5 | 8 | 13 | Escherichia coli | RIBONUCLEOTIDE REDUCTASE R2 | 3V2M_AZM303A- 3DC3_AZM263A- 1ZSB_AZM264A- 3V2J_AZM302A- 3ML5_AZM264A- |
| Acetazolamide | 14 | 2RH2_1 | 5 | 10 | 14 | Escherichia coli | DIHYDROFOLATE REDUCTASE TYPE 2 | 1JD0_AZM1400A- 3DC3_AZM263A- 3W6H_AZM303A- 1ZSB_AZM264A- 1YDB_AZM264A- |
| Acetazolamide | 14 | 1T6U_1 | 5 | 10 | 13 | Streptomyces coelicolor | SUPEROXIDE DISMUTASE [NI] | 3HS4_AZM701A- 3DC3_AZM263A- 1ZSB_AZM264A- 1YDB_AZM264A- 4LU3_AZM302A- |
| Acetazolamide | 14 | 4LPI_1 | 4 | 10 | 12 | Physeter catodon | MYOGLOBIN | 1JD0_AZM1400A- 3HS4_AZM701A- 1RJ6_AZM400A- 1AZM_AZM262A- |
| Acetazolamide | 14 | 4DT5_4 | 4 | 12 | 13 | Rhagium inquisitor | ANTIFREEZE PROTEIN | 3V2M_AZM303A- 1YDA_AZM264A- 3W6H_AZM303A- 1YDB_AZM264A- |
| Acetazolamide | 14 | 2YFU_3 | 4 | 11 | 13 | Ruminiclostridium thermocellum | CARBOHYDRATE BINDING FAMILY 6 | 1JD0_AZM1400A- 3DC3_AZM263A- 1YDB_AZM264A- 3ML5_AZM264A- |
| Acetazolamide | 14 | 4IAX_4 | 4 | 11 | 14 | Homo sapiens | NEUTROPHIL GELATINASE-ASSOCIATED LIPOCALIN | 1JD0_AZM1400A- 3CZV_AZM263A- 1ZSB_AZM264A- 3V2J_AZM302A- |
| Acetazolamide | 14 | 2WGK_1 | 4 | 12 | 13 | - | 3,6-DIKETOCAMPHANE 1,6 MONOOXYGENASE | 1JD0_AZM1400A- 3DC3_AZM263A- 3V2J_AZM302A- 1AZM_AZM262A- |
| Acetazolamide | 14 | 2Z3Q_1 | 4 | 9 | 12 | Homo sapiens | INTERLEUKIN-15 | 1RJ6_AZM400A- 1ZSB_AZM264A- 3V2J_AZM302A- 3ML5_AZM264A- |
| Acetazolamide | 14 | 2OPW_1 | 4 | 8 | 12 | Homo sapiens | PHYHD1 PROTEIN | 3V2M_AZM303A- 1JD0_AZM1400A- 3DC3_AZM263A- 3W6H_AZM303A- |
| Acetazolamide | 14 | 3S8S_3 | 4 | 8 | 13 | Homo sapiens | HISTONE-LYSINE N-METHYLTRANSFERASE SETD1A | 3V2M_AZM303A- 1JD0_AZM1400A- 1YDA_AZM264A- 3ML5_AZM264A- |
| Acetazolamide | 14 | 3HTN_2 | 4 | 10 | 12 | Bacteroides thetaiotaomicron | PUTATIVE DNA BINDING PROTEIN | 3V2M_AZM303A- 3DC3_AZM263A- 3W6H_AZM303A- 3V2J_AZM302A- |
| Acetazolamide | 14 | 3OXP_3 | 3 | 9 | 13 | Yersinia pestis | PHOSPHOTRANSFERASE ENZYME II, A COMPONENT | 3V2M_AZM303A- 1ZSB_AZM264A- 1AZM_AZM262A- |
| Acetazolamide | 14 | 2OXO_2 | 3 | 9 | 12 | unidentified phage | INTEGRASE | 3V2M_AZM303A- 1YDA_AZM264A- 1YDB_AZM264A- |
| Acetazolamide | 14 | 2ZS0_3 | 3 | 4 | 12 | Oligobrachia mashikoi | EXTRACELLULAR GIANT HEMOGLOBIN MAJOR GLOBIN SUBUNIT A1 | 1JD0_AZM1400A- 4LU3_AZM302A- 1AZM_AZM262A- |
| Acetazolamide | 14 | 1RVK_1 | 3 | 9 | 12 | Agrobacterium fabrum | ISOMERASE/LACTONIZING ENZYME | 3V2M_AZM303A- 1JD0_AZM1400A- 1RJ6_AZM400A- |
| Acetazolamide | 14 | 3W0E_2 | 3 | 9 | 13 | Aspergillus fumigatus | ELASTASE INHIBITOR AFUEI | 1JD0_AZM1400A- 3W6H_AZM303A- 1YDB_AZM264A- |
| Acetazolamide | 14 | 2WGK_2 | 3 | 9 | 12 | - | 3,6-DIKETOCAMPHANE 1,6 MONOOXYGENASE | 3HS4_AZM701A- 1ZSB_AZM264A- 3ML5_AZM264A- |
| Acetazolamide | 14 | 3LAG_1 | 3 | 6 | 9 | Rhodopseudomonas palustris | UNCHARACTERIZED PROTEIN RPA4178 | 3HS4_AZM701A- 3DC3_AZM263A- 1ZSB_AZM264A- |
| Acetazolamide | 14 | 1V9Y_2 | 3 | 3 | 6 | Escherichia coli | HEME PAS SENSOR PROTEIN | 3W6H_AZM303A- 1ZSB_AZM264A- 1AZM_AZM262A- |
| Acetazolamide | 14 | 3GIW_1 | 3 | 4 | 10 | Streptomyces avermitilis | PROTEIN OF UNKNOWN FUNCTION DUF574 | 3DC3_AZM263A- 1YDB_AZM264A- 3V2J_AZM302A- |
| Acetazolamide | 14 | 2OZL_2 | 3 | 6 | 11 | Homo sapiens | PYRUVATE DEHYDROGENASE E1 COMPONENT ALPHA | 3W6H_AZM303A- 1ZSB_AZM264A- 3ML5_AZM264A- |
| Acetazolamide | 14 | 2VPT_1 | 3 | 11 | 14 | Ruminiclostridium thermocellum | LIPOLYTIC ENZYME | 1RJ6_AZM400A- 4LU3_AZM302A- 1AZM_AZM262A- |
| Acetazolamide | 14 | 2GDM_1 | 3 | 8 | 11 | Lupinus luteus | LEGHEMOGLOBIN (OXY) | 3W6H_AZM303A- 1ZSB_AZM264A- 1AZM_AZM262A- |
| Acetazolamide | 14 | 1M1Q_1 | 2 | 2 | 5 | Shewanella oneidensis | SMALL TETRAHEME CYTOCHROME C | 3W6H_AZM303A- 3CZV_AZM263A- |
| Acetazolamide | 14 | 3PD7_3 | 2 | 6 | 13 | Homo sapiens | DNA TOPOISOMERASE 2-BINDING PROTEIN 1 | 1JD0_AZM1400A- 3W6H_AZM303A- |
| Acetazolamide | 14 | 1Y0N_2 | 2 | 7 | 14 | Pseudomonas aeruginosa | HYPOTHETICAL UPF0270 PROTEIN PA3463 | 1JD0_AZM1400A- 3DC3_AZM263A- |
| Acetazolamide | 14 | 3F0P_1 | 2 | 8 | 11 | Escherichia coli | ALKYLMERCURY LYASE | 1YDB_AZM264A- 3ML5_AZM264A- |
| Acetazolamide | 14 | 3GT5_2 | 2 | 3 | 10 | Xylella fastidiosa | N-ACETYLGLUCOSAMINE 2-EPIMERASE | 1YDB_AZM264A- 1AZM_AZM262A- |
| Acetazolamide | 14 | 1XSV_3 | 2 | 2 | 5 | Staphylococcus aureus | HYPOTHETICAL UPF0122 PROTEIN SAV1236 | 3CZV_AZM263A- 1AZM_AZM262A- |
| Acetazolamide | 14 | 4LUK_1 | 2 | 8 | 13 | Pyrococcus furiosus | GLUCOSE-6-PHOSPHATE ISOMERASE | 3V2M_AZM303A- 1YDA_AZM264A- |
| Acetazolamide | 14 | 1GMU_1 | 2 | 5 | 7 | Enterobacter aerogenes | UREE | 3V2M_AZM303A- 3HS4_AZM701A- |
| Acetazolamide | 14 | 3PCV_1 | 2 | 3 | 12 | Homo sapiens | LEUKOTRIENE C4 SYNTHASE | 3CZV_AZM263A- 1AZM_AZM262A- |
| Acetazolamide | 14 | 1JNI_1 | 2 | 6 | 9 | Haemophilus influenzae | DIHEME CYTOCHROME C NAPB | 3W6H_AZM303A- 1ZSB_AZM264A- |
| Acetazolamide | 14 | 4NSV_1 | 2 | 7 | 9 | Lysobacter enzymogenes | LYSYL ENDOPEPTIDASE | 3W6H_AZM303A- 1AZM_AZM262A- |
| Acetazolamide | 14 | 1R9W_2 | 2 | 7 | 14 | Alphapapillomavirus 7 | REPLICATION PROTEIN E1 | 3CZV_AZM263A- 1ZSB_AZM264A- |
| Acetazolamide | 14 | 3G0O_1 | 2 | 6 | 9 | Salmonella enterica | 3-HYDROXYISOBUTYRATE DEHYDROGENASE | 3W6H_AZM303A- 3V2J_AZM302A- |
| Acetazolamide | 14 | 3E4W_1 | 2 | 4 | 9 | Mycobacterium avium | PUTATIVE UNCHARACTERIZED PROTEIN | 3V2J_AZM302A- 3ML5_AZM264A- |
| Acetazolamide | 14 | 3H4N_1 | 2 | 3 | 4 | Geobacter sulfurreducens | CYTOCHROME C7 | 3DC3_AZM263A- 3CZV_AZM263A- |
| Acetazolamide | 14 | 4LBH_1 | 2 | 4 | 9 | Burkholderia cepacia | 5-CHLORO-2-HYDROXYHYDROQUINONE DEHYDROCHLORINASE (TFTG) | 1RJ6_AZM400A- 1AZM_AZM262A- |
| Acetazolamide | 14 | 3F7E_6 | 2 | 5 | 12 | Mycobacterium smegmatis | PYRIDOXAMINE 5'-PHOSPHATE OXIDASE-RELATED, FMN- | 1JD0_AZM1400A- 1ZSB_AZM264A- |
| Acetazolamide | 14 | 3Q1N_1 | 2 | 3 | 8 | Lactobacillus casei | GALACTOSE MUTAROTASE RELATED ENZYME | 1YDA_AZM264A- 3W6H_AZM303A- |
| Acetazolamide | 14 | 3QM9_1 | 2 | 9 | 11 | Thunnus atlanticus | MYOGLOBIN | 1JD0_AZM1400A- 1AZM_AZM262A- |
| Acetazolamide | 14 | 3L5L_1 | 2 | 4 | 8 | Pseudomonas putida | XENOBIOTIC REDUCTASE A | 3V2M_AZM303A- 1YDA_AZM264A- |
| Acetazolamide | 14 | 4LLE_4 | 2 | 2 | 9 | Pseudomonas aeruginosa | PROBABLE TWO-COMPONENT SENSOR | 3CZV_AZM263A- 1AZM_AZM262A- |
| Acetazolamide | 14 | 2PA7_2 | 2 | 8 | 11 | Aneurinibacillus thermoaerophilus | DTDP-6-DEOXY-3,4-KETO-HEXULOSE ISOMERASE | 1JD0_AZM1400A- 1ZSB_AZM264A- |
| Acetazolamide | 14 | 3B9T_3 | 2 | 7 | 10 | Methylobacillus flagellatus | TWIN-ARGININE TRANSLOCATION PATHWAY SIGNAL PROTEIN | 3DC3_AZM263A- 1RJ6_AZM400A- |
| Acetazolamide | 14 | 1DCS_1 | 2 | 4 | 10 | Streptomyces clavuligerus | DEACETOXYCEPHALOSPORIN C SYNTHASE | 3V2M_AZM303A- 3HS4_AZM701A- |
| Acetazolamide | 14 | 1ALY_2 | 2 | 9 | 13 | Homo sapiens | CD40 LIGAND | 1RJ6_AZM400A- 4LU3_AZM302A- |
| Acetazolamide | 14 | 3MZ2_1 | 2 | 3 | 9 | Parabacteroides distasonis | GLYCEROPHOSPHORYL DIESTER PHOSPHODIESTERASE | 3DC3_AZM263A- 3W6H_AZM303A- |
| Acetazolamide | 14 | 2CYJ_3 | 2 | 7 | 13 | Pyrococcus horikoshii | HYPOTHETICAL PROTEIN PH1505 | 3W6H_AZM303A- 1YDB_AZM264A- |
| Acetazolamide | 14 | 3TY1_1 | 2 | 7 | 13 | Klebsiella pneumoniae | HYPOTHETICAL ALDOSE 1-EPIMERASE | 1YDA_AZM264A- 1ZSB_AZM264A- |
| Acetazolamide | 14 | 2WJN_1 | 2 | 11 | 13 | Blastochloris viridis | PHOTOSYNTHETIC REACTION CENTER CYTOCHROME C SUBUNIT | 3V2M_AZM303A- 1JD0_AZM1400A- |
| Acetazolamide | 14 | 1ZPD_3 | 2 | 4 | 9 | Zymomonas mobilis | PYRUVATE DECARBOXYLASE | 3V2M_AZM303A- 1YDA_AZM264A- |
| Acetazolamide | 14 | 1BBH_1 | 2 | 3 | 5 | Allochromatium vinosum | CYTOCHROME C' | 3DC3_AZM263A- 3W6H_AZM303A- |
| Acetazolamide | 14 | 3B0T_1 | 2 | 3 | 10 | Homo sapiens | VITAMIN D3 RECEPTOR | 3CZV_AZM263A- 1ZSB_AZM264A- |
| Acetazolamide | 14 | 4OQY_1 | 2 | 3 | 8 | Streptomyces sp. GF3546 | (S)-IMINE REDUCTASE | 3CZV_AZM263A- 1ZSB_AZM264A- |
| Acetazolamide | 14 | 4DOY_3 | 2 | 6 | 10 | Rhodococcus sp. XP | DIBENZOTHIOPHENE DESULFURIZATION ENZYME C | 1JD0_AZM1400A- 3DC3_AZM263A- |
| Acetazolamide | 14 | 4GB5_1 | 2 | 7 | 9 | Kribbella flavida | UNCHARACTERIZED PROTEIN | 3DC3_AZM263A- 1YDB_AZM264A- |
| Acetazolamide | 14 | 3LDC_2 | 2 | 7 | 10 | Methanothermobacter thermautotrophicus | CALCIUM-GATED POTASSIUM CHANNEL MTHK | 1RJ6_AZM400A- 3CZV_AZM263A- |
| Acetazolamide | 14 | 1RFY_1 | 2 | 4 | 9 | Agrobacterium tumefaciens | TRANSCRIPTIONAL REPRESSOR TRAM | 3V2M_AZM303A- 3V2J_AZM302A- |
| Tretinoin | 7 | 3LDC_1 | 7 | 7 | 7 | Methanothermobacter thermautotrophicus | CALCIUM-GATED POTASSIUM CHANNEL MTHK | 3OAP_REA500A- 3LBD_REA424A- 1XLS_REA801A- 4DM8_REA501A- 2LBD_REA500A- 3FC6_REA501C- 1G5Y_REA502C- |
| Tretinoin | 7 | 4PND_2 | 7 | 7 | 7 | synthetic construct | CC-PENT_VARIANT | 3OAP_REA500A- 3LBD_REA424A- 1XLS_REA801A- 4DM8_REA501A- 2LBD_REA500A- 3FC6_REA501C- 1G5Y_REA502C- |
| Tretinoin | 7 | 1H2S_7 | 7 | 7 | 7 | Natronomonas pharaonis | SENSORY RHODOPSIN II | 3OAP_REA500A- 3LBD_REA424A- 1XLS_REA801A- 4DM8_REA501A- 2LBD_REA500A- 3FC6_REA501C- 1G5Y_REA502C- |
| Tretinoin | 7 | 3CLA_2 | 7 | 7 | 7 | Escherichia coli | TYPE III CHLORAMPHENICOL ACETYLTRANSFERASE | 3OAP_REA500A- 3LBD_REA424A- 1XLS_REA801A- 4DM8_REA501A- 2LBD_REA500A- 3FC6_REA501C- 1G5Y_REA502C- |
| Tretinoin | 7 | 3K3V_2 | 7 | 7 | 7 | Saccharomyces cerevisiae | PROTEIN SMY2 | 3OAP_REA500A- 3LBD_REA424A- 1XLS_REA801A- 4DM8_REA501A- 2LBD_REA500A- 3FC6_REA501C- 1G5Y_REA502C- |
| Tretinoin | 7 | 3JR7_5 | 7 | 7 | 7 | [Ruminococcus] gnavus | UNCHARACTERIZED EGV FAMILY PROTEIN COG1307 | 3OAP_REA500A- 3LBD_REA424A- 1XLS_REA801A- 4DM8_REA501A- 2LBD_REA500A- 3FC6_REA501C- 1G5Y_REA502C- |
| Tretinoin | 7 | 4PF3_2 | 7 | 7 | 7 | Homo sapiens | MINERALOCORTICOID RECEPTOR | 3OAP_REA500A- 3LBD_REA424A- 1XLS_REA801A- 4DM8_REA501A- 2LBD_REA500A- 3FC6_REA501C- 1G5Y_REA502C- |
| Tretinoin | 7 | 2P09_3 | 7 | 7 | 7 | unidentified | A NON-BIOLOGICAL ATP BINDING PROTEIN WITH TWO MUTATIONS | 3OAP_REA500A- 3LBD_REA424A- 1XLS_REA801A- 4DM8_REA501A- 2LBD_REA500A- 3FC6_REA501C- 1G5Y_REA502C- |
| Tretinoin | 7 | 1Y6X_1 | 7 | 7 | 7 | Mycobacterium tuberculosis | PHOSPHORIBOSYL-ATP PYROPHOSPHATASE | 3OAP_REA500A- 3LBD_REA424A- 1XLS_REA801A- 4DM8_REA501A- 2LBD_REA500A- 3FC6_REA501C- 1G5Y_REA502C- |
| Tretinoin | 7 | 2J7Q_5 | 7 | 7 | 7 | Murid herpesvirus 1 | UBIQUITIN | 3OAP_REA500A- 3LBD_REA424A- 1XLS_REA801A- 4DM8_REA501A- 2LBD_REA500A- 3FC6_REA501C- 1G5Y_REA502C- |
| Tretinoin | 7 | 4UYB_4 | 7 | 7 | 7 | Homo sapiens | SEC14-LIKE PROTEIN 3 | 3OAP_REA500A- 3LBD_REA424A- 1XLS_REA801A- 4DM8_REA501A- 2LBD_REA500A- 3FC6_REA501C- 1G5Y_REA502C- |
| Tretinoin | 7 | 3FGV_1 | 7 | 7 | 7 | Ruegeria pomeroyi | UNCHARACTERIZED PROTEIN WITH FERREDOXIN-LIKE FOLD | 3OAP_REA500A- 3LBD_REA424A- 1XLS_REA801A- 4DM8_REA501A- 2LBD_REA500A- 3FC6_REA501C- 1G5Y_REA502C- |
| Tretinoin | 7 | 2DWK_5 | 7 | 7 | 7 | Mus musculus | PROTEIN RUFY3 | 3OAP_REA500A- 3LBD_REA424A- 1XLS_REA801A- 4DM8_REA501A- 2LBD_REA500A- 3FC6_REA501C- 1G5Y_REA502C- |
| Tretinoin | 7 | 4TW3_1 | 7 | 7 | 7 | Prochlorococcus marinus | ALDEHYDE DECARBONYLASE | 3OAP_REA500A- 3LBD_REA424A- 1XLS_REA801A- 4DM8_REA501A- 2LBD_REA500A- 3FC6_REA501C- 1G5Y_REA502C- |
| Tretinoin | 7 | 1Y9L_1 | 7 | 7 | 7 | Shigella flexneri | LIPOPROTEIN MXIM | 3OAP_REA500A- 3LBD_REA424A- 1XLS_REA801A- 4DM8_REA501A- 2LBD_REA500A- 3FC6_REA501C- 1G5Y_REA502C- |
| Tretinoin | 7 | 2EH3_1 | 7 | 7 | 7 | Aquifex aeolicus | TRANSCRIPTIONAL REGULATOR | 3OAP_REA500A- 3LBD_REA424A- 1XLS_REA801A- 4DM8_REA501A- 2LBD_REA500A- 3FC6_REA501C- 1G5Y_REA502C- |
| Tretinoin | 7 | 1PZX_4 | 7 | 7 | 7 | Geobacillus stearothermophilus | HYPOTHETICAL PROTEIN APC36103 | 3OAP_REA500A- 3LBD_REA424A- 1XLS_REA801A- 4DM8_REA501A- 2LBD_REA500A- 3FC6_REA501C- 1G5Y_REA502C- |
| Tretinoin | 7 | 3FSS_5 | 7 | 7 | 7 | Saccharomyces cerevisiae | HISTONE CHAPERONE RTT106 | 3OAP_REA500A- 3LBD_REA424A- 1XLS_REA801A- 4DM8_REA501A- 2LBD_REA500A- 3FC6_REA501C- 1G5Y_REA502C- |
| Tretinoin | 7 | 4Q6V_2 | 7 | 7 | 7 | Salmonella enterica | PENICILLIN-BINDING PROTEIN ACTIVATOR LPOB | 3OAP_REA500A- 3LBD_REA424A- 1XLS_REA801A- 4DM8_REA501A- 2LBD_REA500A- 3FC6_REA501C- 1G5Y_REA502C- |
| Tretinoin | 7 | 4PND_1 | 7 | 7 | 7 | synthetic construct | CC-PENT_VARIANT | 3OAP_REA500A- 3LBD_REA424A- 1XLS_REA801A- 4DM8_REA501A- 2LBD_REA500A- 3FC6_REA501C- 1G5Y_REA502C- |
| Tretinoin | 7 | 4U12_2 | 7 | 7 | 7 | Helicobacter pylori | UNCHARACTERIZED PROTEIN HP0242 | 3OAP_REA500A- 3LBD_REA424A- 1XLS_REA801A- 4DM8_REA501A- 2LBD_REA500A- 3FC6_REA501C- 1G5Y_REA502C- |
| Tretinoin | 7 | 3EBY_2 | 7 | 7 | 7 | Novosphingobium aromaticivorans | BETA SUBUNIT OF A PUTATIVE AROMATIC-RING-HYDROXYLATING | 3OAP_REA500A- 3LBD_REA424A- 1XLS_REA801A- 4DM8_REA501A- 2LBD_REA500A- 3FC6_REA501C- 1G5Y_REA502C- |
| Tretinoin | 7 | 4NOH_3 | 7 | 7 | 7 | Bacillus anthracis | LIPOPROTEIN, PUTATIVE | 3OAP_REA500A- 3LBD_REA424A- 1XLS_REA801A- 4DM8_REA501A- 2LBD_REA500A- 3FC6_REA501C- 1G5Y_REA502C- |
| Tretinoin | 7 | 3GWK_3 | 7 | 7 | 7 | Streptococcus agalactiae | PUTATIVE UNCHARACTERIZED PROTEIN SAG1039 | 3OAP_REA500A- 3LBD_REA424A- 1XLS_REA801A- 4DM8_REA501A- 2LBD_REA500A- 3FC6_REA501C- 1G5Y_REA502C- |
| Tretinoin | 7 | 1VMG_2 | 7 | 7 | 7 | Sulfolobus solfataricus | HYPOTHETICAL PROTEIN SSO3215 | 3OAP_REA500A- 3LBD_REA424A- 1XLS_REA801A- 4DM8_REA501A- 2LBD_REA500A- 3FC6_REA501C- 1G5Y_REA502C- |
| Tretinoin | 7 | 2B97_1 | 7 | 7 | 7 | Trichoderma reesei | HYDROPHOBIN II | 3OAP_REA500A- 3LBD_REA424A- 1XLS_REA801A- 4DM8_REA501A- 2LBD_REA500A- 3FC6_REA501C- 1G5Y_REA502C- |
| Tretinoin | 7 | 2XGV_4 | 7 | 7 | 7 | Microcebus murinus | PSIV CAPSID N-TERMINAL DOMAIN | 3OAP_REA500A- 3LBD_REA424A- 1XLS_REA801A- 4DM8_REA501A- 2LBD_REA500A- 3FC6_REA501C- 1G5Y_REA502C- |
| Tretinoin | 7 | 3G0O_2 | 7 | 7 | 7 | Salmonella enterica | 3-HYDROXYISOBUTYRATE DEHYDROGENASE | 3OAP_REA500A- 3LBD_REA424A- 1XLS_REA801A- 4DM8_REA501A- 2LBD_REA500A- 3FC6_REA501C- 1G5Y_REA502C- |
| Tretinoin | 7 | 2XOV_2 | 7 | 7 | 7 | Escherichia coli | RHOMBOID PROTEASE GLPG | 3OAP_REA500A- 3LBD_REA424A- 1XLS_REA801A- 4DM8_REA501A- 2LBD_REA500A- 3FC6_REA501C- 1G5Y_REA502C- |
| Tretinoin | 7 | 2RFF_4 | 7 | 7 | 7 | Sulfolobus solfataricus | PUTATIVE NUCLEOTIDYLTRANSFERASE | 3OAP_REA500A- 3LBD_REA424A- 1XLS_REA801A- 4DM8_REA501A- 2LBD_REA500A- 3FC6_REA501C- 1G5Y_REA502C- |
| Tretinoin | 7 | 4PZJ_2 | 6 | 7 | 7 | Eggerthella lenta | TRANSCRIPTIONAL REGULATOR, LYSR FAMILY | 3OAP_REA500A- 3LBD_REA424A- 1XLS_REA801A- 4DM8_REA501A- 2LBD_REA500A- 3FC6_REA501C- |
| Tretinoin | 7 | 1R5L_1 | 6 | 7 | 7 | Homo sapiens | PROTEIN (ALPHA-TOCOPHEROL TRANSFER PROTEIN) | 3OAP_REA500A- 1XLS_REA801A- 4DM8_REA501A- 2LBD_REA500A- 3FC6_REA501C- 1G5Y_REA502C- |
| Tretinoin | 7 | 1H2S_6 | 6 | 7 | 7 | Natronomonas pharaonis | SENSORY RHODOPSIN II | 3LBD_REA424A- 1XLS_REA801A- 4DM8_REA501A- 2LBD_REA500A- 3FC6_REA501C- 1G5Y_REA502C- |
| Tretinoin | 7 | 3S0A_1 | 6 | 7 | 7 | Apis mellifera | OBP14 | 3OAP_REA500A- 3LBD_REA424A- 1XLS_REA801A- 4DM8_REA501A- 2LBD_REA500A- 3FC6_REA501C- |
| Tretinoin | 7 | 4HYL_1 | 6 | 7 | 7 | Haliangium ochraceum | STAGE II SPORULATION PROTEIN | 3OAP_REA500A- 3LBD_REA424A- 1XLS_REA801A- 4DM8_REA501A- 2LBD_REA500A- 3FC6_REA501C- |
| Tretinoin | 7 | 3RFI_2 | 6 | 7 | 7 | Solanum tuberosum | ASP | 3OAP_REA500A- 1XLS_REA801A- 4DM8_REA501A- 2LBD_REA500A- 3FC6_REA501C- 1G5Y_REA502C- |
| Tretinoin | 7 | 2Q12_1 | 6 | 6 | 7 | Homo sapiens | DCC-INTERACTING PROTEIN 13 ALPHA | 3OAP_REA500A- 3LBD_REA424A- 1XLS_REA801A- 4DM8_REA501A- 2LBD_REA500A- 3FC6_REA501C- |
| Tretinoin | 7 | 2Z1C_3 | 6 | 7 | 7 | Thermococcus kodakarensis | HYDROGENASE EXPRESSION/FORMATION PROTEIN HYPC | 3OAP_REA500A- 3LBD_REA424A- 1XLS_REA801A- 4DM8_REA501A- 2LBD_REA500A- 1G5Y_REA502C- |
| Tretinoin | 7 | 2OU6_4 | 6 | 7 | 7 | Deinococcus radiodurans | HYPOTHETICAL PROTEIN | 3OAP_REA500A- 3LBD_REA424A- 1XLS_REA801A- 4DM8_REA501A- 2LBD_REA500A- 3FC6_REA501C- |
| Tretinoin | 7 | 1XIO_1 | 6 | 7 | 7 | Nostoc sp. PCC 7120 | ANABAENA SENSORY RHODOPSIN | 3OAP_REA500A- 3LBD_REA424A- 1XLS_REA801A- 4DM8_REA501A- 2LBD_REA500A- 3FC6_REA501C- |
| Tretinoin | 7 | 1EW0_5 | 6 | 7 | 7 | Sinorhizobium meliloti | FIXL | 3OAP_REA500A- 3LBD_REA424A- 1XLS_REA801A- 2LBD_REA500A- 3FC6_REA501C- 1G5Y_REA502C- |
| Tretinoin | 7 | 4U9B_3 | 6 | 7 | 7 | Shewanella oneidensis | NO-BINDING HEME-DEPENDENT SENSOR PROTEIN | 3OAP_REA500A- 3LBD_REA424A- 1XLS_REA801A- 4DM8_REA501A- 3FC6_REA501C- 1G5Y_REA502C- |
| Tretinoin | 7 | 4A5X_4 | 6 | 7 | 7 | Homo sapiens | MIT DOMAIN-CONTAINING PROTEIN 1 | 3OAP_REA500A- 3LBD_REA424A- 1XLS_REA801A- 4DM8_REA501A- 2LBD_REA500A- 3FC6_REA501C- |
| Tretinoin | 7 | 1SN9_2 | 6 | 7 | 7 | - | TETRAMERIC BETA-BETA-ALPHA MINI-PROTEIN | 3OAP_REA500A- 3LBD_REA424A- 1XLS_REA801A- 4DM8_REA501A- 3FC6_REA501C- 1G5Y_REA502C- |
| Tretinoin | 7 | 4M5B_4 | 6 | 7 | 7 | Caldanaerobacter subterraneus | COBALAMIN BIOSYNTHESIS PROTEIN CBIM | 3LBD_REA424A- 1XLS_REA801A- 4DM8_REA501A- 2LBD_REA500A- 3FC6_REA501C- 1G5Y_REA502C- |
| Tretinoin | 7 | 3QP4_1 | 5 | 7 | 7 | Chromobacterium violaceum | CVIR TRANSCRIPTIONAL REGULATOR | 3OAP_REA500A- 1XLS_REA801A- 4DM8_REA501A- 2LBD_REA500A- 3FC6_REA501C- |
| Tretinoin | 7 | 3PCV_1 | 5 | 7 | 7 | Homo sapiens | LEUKOTRIENE C4 SYNTHASE | 3LBD_REA424A- 1XLS_REA801A- 4DM8_REA501A- 2LBD_REA500A- 3FC6_REA501C- |
| Tretinoin | 7 | 2CX7_2 | 5 | 7 | 7 | Thermus thermophilus | STEROL CARRIER PROTEIN 2 | 3OAP_REA500A- 3LBD_REA424A- 1XLS_REA801A- 4DM8_REA501A- 1G5Y_REA502C- |
| Tretinoin | 7 | 3O48_2 | 5 | 7 | 7 | Saccharomyces cerevisiae | MITOCHONDRIA FISSION 1 PROTEIN | 3LBD_REA424A- 1XLS_REA801A- 4DM8_REA501A- 2LBD_REA500A- 1G5Y_REA502C- |
| Tretinoin | 7 | 4TW3_5 | 5 | 7 | 7 | Prochlorococcus marinus | ALDEHYDE DECARBONYLASE | 3OAP_REA500A- 1XLS_REA801A- 4DM8_REA501A- 3FC6_REA501C- 1G5Y_REA502C- |
| Tretinoin | 7 | 4I2L_2 | 5 | 7 | 7 | Human immunodeficiency virus 1 | GP41 | 3OAP_REA500A- 1XLS_REA801A- 4DM8_REA501A- 2LBD_REA500A- 3FC6_REA501C- |
| Tretinoin | 7 | 4J8C_1 | 5 | 7 | 7 | Rattus norvegicus | HSC70-INTERACTING PROTEIN | 3OAP_REA500A- 3LBD_REA424A- 1XLS_REA801A- 2LBD_REA500A- 1G5Y_REA502C- |
| Tretinoin | 7 | 3DKR_2 | 4 | 7 | 7 | Lactobacillus rhamnosus | ESTERASE D | 3OAP_REA500A- 1XLS_REA801A- 3FC6_REA501C- 1G5Y_REA502C- |
| Tretinoin | 7 | 2QIB_2 | 4 | 7 | 7 | Streptomyces coelicolor | TETR-FAMILY TRANSCRIPTIONAL REGULATOR | 3LBD_REA424A- 1XLS_REA801A- 3FC6_REA501C- 1G5Y_REA502C- |
| Tretinoin | 7 | 1MZ9_1 | 4 | 7 | 7 | Mus musculus | CARTILAGE OLIGOMERIC MATRIX PROTEIN | 3OAP_REA500A- 1XLS_REA801A- 4DM8_REA501A- 3FC6_REA501C- |
| Tretinoin | 7 | 1PZ4_1 | 4 | 7 | 7 | Aedes aegypti | STEROL CARRIER PROTEIN 2 | 3OAP_REA500A- 3LBD_REA424A- 1XLS_REA801A- 3FC6_REA501C- |
| Tretinoin | 7 | 3CZ1_2 | 4 | 7 | 7 | Apis mellifera | PHEROMONE-BINDING PROTEIN ASP1 | 3OAP_REA500A- 1XLS_REA801A- 4DM8_REA501A- 3FC6_REA501C- |
| Tretinoin | 7 | 1UIX_1 | 4 | 6 | 7 | Bos taurus | RHO-ASSOCIATED KINASE | 3OAP_REA500A- 1XLS_REA801A- 4DM8_REA501A- 3FC6_REA501C- |
| Tretinoin | 7 | 3K3C_4 | 4 | 7 | 7 | Mycobacterium tuberculosis | PROTEIN RV1364C/MT1410 | 3OAP_REA500A- 1XLS_REA801A- 4DM8_REA501A- 3FC6_REA501C- |
| Tretinoin | 7 | 1VI4_1 | 4 | 6 | 7 | Vibrio cholerae | REGULATOR OF RIBONUCLEASE ACIVITY A PROTEIN 1 | 3OAP_REA500A- 3LBD_REA424A- 1XLS_REA801A- 3FC6_REA501C- |
| Tretinoin | 7 | 3CIM_3 | 4 | 7 | 7 | Synechocystis sp. | CARBON DIOXIDE-CONCENTRATING MECHANISM PROTEIN CCMK HOMOLOG | 3OAP_REA500A- 1XLS_REA801A- 3FC6_REA501C- 1G5Y_REA502C- |
| Tretinoin | 7 | 3OST_1 | 4 | 7 | 7 | Saccharomyces cerevisiae | SERINE/THREONINE-PROTEIN KINASE KCC4 | 3OAP_REA500A- 1XLS_REA801A- 4DM8_REA501A- 3FC6_REA501C- |
| Tretinoin | 7 | 4GQM_2 | 4 | 7 | 7 | Chlamydia trachomatis | CT009 | 3OAP_REA500A- 4DM8_REA501A- 2LBD_REA500A- 1G5Y_REA502C- |
| Tretinoin | 7 | 4PN8_3 | 4 | 7 | 7 | synthetic construct | CC-PENT | 3LBD_REA424A- 1XLS_REA801A- 4DM8_REA501A- 1G5Y_REA502C- |
| Tretinoin | 7 | 1I5G_1 | 3 | 6 | 7 | Crithidia fasciculata | TRYPAREDOXIN II | 3OAP_REA500A- 1XLS_REA801A- 3FC6_REA501C- |
| Tretinoin | 7 | 4INW_1 | 3 | 7 | 7 | Amyelois transitella | PHEROMONE-BINDING PROTEIN 1 | 1XLS_REA801A- 4DM8_REA501A- 3FC6_REA501C- |
| Tretinoin | 7 | 2RH3_2 | 3 | 5 | 7 | Agrobacterium tumefaciens | PROTEIN VIRC2 | 3OAP_REA500A- 1XLS_REA801A- 4DM8_REA501A- |
| Tretinoin | 7 | 1NQ7_1 | 3 | 6 | 7 | Rattus norvegicus | NUCLEAR RECEPTOR ROR-BETA | 3OAP_REA500A- 1XLS_REA801A- 3FC6_REA501C- |
| Tretinoin | 7 | 3LK7_3 | 3 | 7 | 7 | Streptococcus agalactiae | UDP-N-ACETYLMURAMOYLALANINE--D-GLUTAMATE LIGASE | 3LBD_REA424A- 4DM8_REA501A- 2LBD_REA500A- |
| Tretinoin | 7 | 3JSR_1 | 3 | 7 | 7 | Nostoc sp. PCC 7120 | ALL0216 PROTEIN | 3LBD_REA424A- 1XLS_REA801A- 3FC6_REA501C- |
| Tretinoin | 7 | 2YZQ_2 | 3 | 6 | 7 | Pyrococcus horikoshii | PUTATIVE UNCHARACTERIZED PROTEIN PH1780 | 1XLS_REA801A- 4DM8_REA501A- 1G5Y_REA502C- |
| Tretinoin | 7 | 3AH9_2 | 3 | 7 | 7 | Saimiriine herpesvirus 2 | COLLAGEN-LIKE PEPTIDE | 3OAP_REA500A- 1XLS_REA801A- 1G5Y_REA502C- |
| Tretinoin | 7 | 1YBK_2 | 3 | 7 | 7 | Staphylothermus marinus | TETRABRACHION | 3OAP_REA500A- 1XLS_REA801A- 3FC6_REA501C- |
| Tretinoin | 7 | 3W7Y_1 | 3 | 7 | 7 | Homo sapiens | INSULIN | 3LBD_REA424A- 2LBD_REA500A- 1G5Y_REA502C- |
| Tretinoin | 7 | 3A0M_3 | 3 | 6 | 7 | - | COLLAGEN-LIKE PEPTIDE | 3OAP_REA500A- 1XLS_REA801A- 4DM8_REA501A- |
| Tretinoin | 7 | 2G3W_4 | 3 | 7 | 7 | Xanthomonas citri | HYPOTHETICAL PROTEIN XAC2396 | 3OAP_REA500A- 1XLS_REA801A- 3FC6_REA501C- |
| Tretinoin | 7 | 3AH9_3 | 2 | 6 | 7 | Saimiriine herpesvirus 2 | COLLAGEN-LIKE PEPTIDE | 1XLS_REA801A- 1G5Y_REA502C- |
| Tretinoin | 7 | 1KU3_1 | 2 | 5 | 7 | Thermus aquaticus | SIGMA FACTOR SIGA | 1XLS_REA801A- 3FC6_REA501C- |
| Tretinoin | 7 | 2B3G_4 | 2 | 6 | 7 | Homo sapiens | REPLICATION PROTEIN A 70 KDA DNA-BINDING SUBUNIT | 2LBD_REA500A- 1G5Y_REA502C- |
| Tretinoin | 7 | 1WN2_1 | 2 | 4 | 7 | Pyrococcus horikoshii | PEPTIDYL-TRNA HYDROLASE | 3LBD_REA424A- 1G5Y_REA502C- |
| Tretinoin | 7 | 3NUF_1 | 2 | 5 | 7 | Lactobacillus casei | PRD-CONTAINING TRANSCRIPTION REGULATOR | 4DM8_REA501A- 3FC6_REA501C- |
| Tretinoin | 7 | 2MHR_2 | 2 | 7 | 7 | Themiste hennahi | MYOHEMERYTHRIN | 3OAP_REA500A- 3FC6_REA501C- |
| Tretinoin | 7 | 3O6P_3 | 2 | 7 | 7 | Enterococcus faecalis | PEPTIDE ABC TRANSPORTER, PEPTIDE-BINDING PROTEIN | 3LBD_REA424A- 1G5Y_REA502C- |
| Tretinoin | 7 | 4B21_3 | 2 | 7 | 7 | Schizosaccharomyces pombe-synthetic construct | PROBABLE DNA-3-METHYLADENINE GLYCOSYLASE 2 | 3OAP_REA500A- 4DM8_REA501A- |
| Tretinoin | 7 | 3LJW_2 | 2 | 5 | 7 | Homo sapiens | PROTEIN POLYBROMO-1 | 3LBD_REA424A- 2LBD_REA500A- |
| Tretinoin | 7 | 3Q8G_1 | 2 | 6 | 7 | Saccharomyces cerevisiae | CRAL-TRIO DOMAIN-CONTAINING PROTEIN YKL091C | 1XLS_REA801A- 3FC6_REA501C- |
| Tretinoin | 7 | 2Z0Q_2 | 2 | 4 | 7 | Mus musculus | RHO GUANINE NUCLEOTIDE EXCHANGE FACTOR 3 | 1XLS_REA801A- 3FC6_REA501C- |
| Tretinoin | 7 | 4BOQ_1 | 2 | 7 | 7 | Homo sapiens | UBIQUITIN THIOESTERASE OTU1 | 1XLS_REA801A- 4DM8_REA501A- |
| Tretinoin | 7 | 1H4R_5 | 2 | 3 | 5 | Homo sapiens | MERLIN | 1XLS_REA801A- 3FC6_REA501C- |
| Tretinoin | 7 | 3GBY_5 | 2 | 5 | 7 | Chlorobaculum tepidum | UNCHARACTERIZED PROTEIN CT1051 | 3LBD_REA424A- 2LBD_REA500A- |
| Tretinoin | 7 | 3A0M_2 | 2 | 6 | 7 | - | COLLAGEN-LIKE PEPTIDE | 3OAP_REA500A- 1XLS_REA801A- |
| Tretinoin | 7 | 3ZJA_5 | 2 | 7 | 7 | Streptomyces lividans | SL3965 | 4DM8_REA501A- 2LBD_REA500A- |
| Tretinoin | 7 | 2WP7_1 | 2 | 5 | 7 | Mus musculus | PPPDE PEPTIDASE DOMAIN-CONTAINING PROTEIN 2 | 1XLS_REA801A- 3FC6_REA501C- |
| Tretinoin | 7 | 3L15_2 | 2 | 3 | 7 | Homo sapiens | TRANSCRIPTIONAL ENHANCER FACTOR TEF-4 | 3OAP_REA500A- 1XLS_REA801A- |
| Tretinoin | 7 | 1TUK_2 | 2 | 7 | 7 | Triticum aestivum | NONSPECIFIC LIPID-TRANSFER PROTEIN 2G | 4DM8_REA501A- 1G5Y_REA502C- |
| Tretinoin | 7 | 4LWU_1 | 2 | 6 | 7 | Xenopus laevis | E3 UBIQUITIN-PROTEIN LIGASE MDM2 | 1XLS_REA801A- 1G5Y_REA502C- |
| Tretinoin | 7 | 4A20_2 | 2 | 6 | 7 | Saccharomyces cerevisiae | UBIQUITIN-LIKE PROTEIN MDY2 | 3LBD_REA424A- 1XLS_REA801A- |
| Tretinoin | 7 | 3ECF_5 | 2 | 7 | 7 | Anabaena variabilis | NTF2-LIKE PROTEIN | 1XLS_REA801A- 2LBD_REA500A- |
| Tretinoin | 7 | 3QW9_7 | 2 | 5 | 7 | Rattus norvegicus | TRANSFORMING GROWTH FACTOR BETA RECEPTOR TYPE 3 | 3OAP_REA500A- 1XLS_REA801A- |
| Tretinoin | 7 | 2NS9_2 | 2 | 6 | 7 | Aeropyrum pernix | HYPOTHETICAL PROTEIN APE2225 | 1XLS_REA801A- 3FC6_REA501C- |
| Zanamivir | 6 | 1F8E_1 | 6 | 6 | 6 | Influenza A virus | NEURAMINIDASE | 4CPZ_ZMR1470C- 1A4G_ZMR466B- 4I00_ZMR509A- 4CPN_ZMR700A- 2CML_ZMR4477D- 3TIC_ZMR1002C- |
| Zanamivir | 6 | 4GMQ_1 | 4 | 6 | 6 | Chaetomium thermophilum | PUTATIVE RIBOSOME ASSOCIATED PROTEIN | 4CPZ_ZMR1470C- 4I00_ZMR509A- 4CPN_ZMR700A- 2CML_ZMR4477D- |
| Zanamivir | 6 | 3PAS_1 | 4 | 6 | 6 | Marinobacter hydrocarbonoclasticus | TETR FAMILY TRANSCRIPTION REGULATOR | 1A4G_ZMR466B- 4CPN_ZMR700A- 2CML_ZMR4477D- 3TIC_ZMR1002C- |
| Zanamivir | 6 | 1YJ7_2 | 3 | 6 | 6 | Escherichia coli | ESCJ | 1A4G_ZMR466B- 4I00_ZMR509A- 4CPN_ZMR700A- |
| Zanamivir | 6 | 1PZ7_5 | 3 | 5 | 6 | Gallus gallus | AGRIN | 4CPZ_ZMR1470C- 4I00_ZMR509A- 4CPN_ZMR700A- |
| Zanamivir | 6 | 1PZ7_1 | 3 | 5 | 6 | Gallus gallus | AGRIN | 1A4G_ZMR466B- 4I00_ZMR509A- 2CML_ZMR4477D- |
| Zanamivir | 6 | 2YXB_5 | 3 | 6 | 6 | Aeropyrum pernix | COENZYME B12-DEPENDENT MUTASE | 4I00_ZMR509A- 4CPN_ZMR700A- 2CML_ZMR4477D- |
| Zanamivir | 6 | 1M1H_2 | 2 | 6 | 6 | Aquifex aeolicus | TRANSCRIPTION ANTITERMINATION PROTEIN NUSG | 1A4G_ZMR466B- 4I00_ZMR509A- |
| Zanamivir | 6 | 3NZ3_2 | 2 | 6 | 6 | Streptococcus pneumoniae | PUTATIVE UNCHARACTERIZED PROTEIN | 4I00_ZMR509A- 2CML_ZMR4477D- |
| Zanamivir | 6 | 3CS1_2 | 2 | 4 | 6 | Trypanosoma cruzi | FLAGELLAR CALCIUM-BINDING PROTEIN | 4I00_ZMR509A- 4CPN_ZMR700A- |
| Zanamivir | 6 | 1YB3_3 | 2 | 5 | 6 | Pyrococcus furiosus | HYPOTHETICAL PROTEIN | 4CPZ_ZMR1470C- 4CPN_ZMR700A- |
| Tetrahydrobiopterin | 6 | 4PVK_1 | 3 | 5 | 6 | Phleum pratense | POLLEN ALLERGEN PHL P 4.0202 | 1KW0_H4B429A- 4D1O_H4B1481B- 3NOS_H4B511A- |
| Tetrahydrobiopterin | 6 | 3KG4_4 | 3 | 3 | 5 | [Mannheimia] succiniciproducens | UNCHARACTERIZED PROTEIN | 4D1P_H4B600B- 4D1O_H4B1481B- 3NOS_H4B511A- |
| Tetrahydrobiopterin | 6 | 1SFP_1 | 2 | 4 | 6 | Bos taurus | ASFP | 4D1P_H4B600B- 4D1O_H4B1481B- |
| Tetrahydrobiopterin | 6 | 4F8L_2 | 2 | 3 | 6 | Yersinia pestis | PH 6 ANTIGEN | 4D1P_H4B600B- 4D1O_H4B1481B- |
| Tetrahydrobiopterin | 6 | 3F0P_1 | 2 | 4 | 6 | Escherichia coli | ALKYLMERCURY LYASE | 4D1P_H4B600B- 3NOS_H4B511A- |
| Vorinostat | 6 | 3QM9_1 | 3 | 6 | 6 | Thunnus atlanticus | MYOGLOBIN | 4QA2_SHH404A- 4QA0_SHH404A- 1C3S_SHH952A- |
| Vorinostat | 6 | 1TUH_1 | 2 | 5 | 6 | uncultured bacterium | HYPOTHETICAL PROTEIN EGC068 | 1ZZ1_SHH2452A- 4QA0_SHH404A- |
| Vorinostat | 6 | 4LWU_1 | 2 | 3 | 4 | Xenopus laevis | E3 UBIQUITIN-PROTEIN LIGASE MDM2 | 4QA2_SHH404A- 1C3S_SHH952A- |
| Vorinostat | 6 | 2XCI_3 | 2 | 3 | 6 | Aquifex aeolicus | 3-DEOXY-D-MANNO-2-OCTULOSONIC ACID TRANSFERASE | 3C0Z_SHH301B- 1C3S_SHH952A- |
| Estradiol | 6 | 1WV3_1 | 6 | 6 | 6 | Staphylococcus aureus | SIMILAR TO DNA SEGREGATION ATPASE AND RELATED | 3UUD_EST600B- 2OCF_EST596A- 3OLL_EST600B- 3OLS_EST600B- 4J24_EST600C- 2YJA_EST1550B- |
| Estradiol | 6 | 4TW3_5 | 6 | 6 | 6 | Prochlorococcus marinus | ALDEHYDE DECARBONYLASE | 3UUD_EST600B- 2OCF_EST596A- 3OLL_EST600B- 3OLS_EST600B- 4J24_EST600C- 2YJA_EST1550B- |
| Estradiol | 6 | 3FSS_5 | 6 | 6 | 6 | Saccharomyces cerevisiae | HISTONE CHAPERONE RTT106 | 3UUD_EST600B- 2OCF_EST596A- 3OLL_EST600B- 3OLS_EST600B- 4J24_EST600C- 2YJA_EST1550B- |
| Estradiol | 6 | 4PND_2 | 6 | 6 | 6 | synthetic construct | CC-PENT_VARIANT | 3UUD_EST600B- 2OCF_EST596A- 3OLL_EST600B- 3OLS_EST600B- 4J24_EST600C- 2YJA_EST1550B- |
| Estradiol | 6 | 1H2S_7 | 6 | 6 | 6 | Natronomonas pharaonis | SENSORY RHODOPSIN II | 3UUD_EST600B- 2OCF_EST596A- 3OLL_EST600B- 3OLS_EST600B- 4J24_EST600C- 2YJA_EST1550B- |
| Estradiol | 6 | 1YBK_2 | 6 | 6 | 6 | Staphylothermus marinus | TETRABRACHION | 3UUD_EST600B- 2OCF_EST596A- 3OLL_EST600B- 3OLS_EST600B- 4J24_EST600C- 2YJA_EST1550B- |
| Estradiol | 6 | 1H2S_6 | 6 | 6 | 6 | Natronomonas pharaonis | SENSORY RHODOPSIN II | 3UUD_EST600B- 2OCF_EST596A- 3OLL_EST600B- 3OLS_EST600B- 4J24_EST600C- 2YJA_EST1550B- |
| Estradiol | 6 | 4U9B_3 | 6 | 6 | 6 | Shewanella oneidensis | NO-BINDING HEME-DEPENDENT SENSOR PROTEIN | 3UUD_EST600B- 2OCF_EST596A- 3OLL_EST600B- 3OLS_EST600B- 4J24_EST600C- 2YJA_EST1550B- |
| Estradiol | 6 | 3S0A_1 | 6 | 6 | 6 | Apis mellifera | OBP14 | 3UUD_EST600B- 2OCF_EST596A- 3OLL_EST600B- 3OLS_EST600B- 4J24_EST600C- 2YJA_EST1550B- |
| Estradiol | 6 | 4I66_2 | 6 | 6 | 6 | Haliangium ochraceum | UNCHARACTERIZED PROTEIN HOCH_4089 | 3UUD_EST600B- 2OCF_EST596A- 3OLL_EST600B- 3OLS_EST600B- 4J24_EST600C- 2YJA_EST1550B- |
| Estradiol | 6 | 1XSV_3 | 6 | 6 | 6 | Staphylococcus aureus | HYPOTHETICAL UPF0122 PROTEIN SAV1236 | 3UUD_EST600B- 2OCF_EST596A- 3OLL_EST600B- 3OLS_EST600B- 4J24_EST600C- 2YJA_EST1550B- |
| Estradiol | 6 | 2O71_1 | 6 | 6 | 6 | Homo sapiens | DEATH DOMAIN-CONTAINING PROTEIN CRADD | 3UUD_EST600B- 2OCF_EST596A- 3OLL_EST600B- 3OLS_EST600B- 4J24_EST600C- 2YJA_EST1550B- |
| Estradiol | 6 | 3RFI_2 | 6 | 6 | 6 | Solanum tuberosum | ASP | 3UUD_EST600B- 2OCF_EST596A- 3OLL_EST600B- 3OLS_EST600B- 4J24_EST600C- 2YJA_EST1550B- |
| Estradiol | 6 | 2DWK_5 | 6 | 6 | 6 | Mus musculus | PROTEIN RUFY3 | 3UUD_EST600B- 2OCF_EST596A- 3OLL_EST600B- 3OLS_EST600B- 4J24_EST600C- 2YJA_EST1550B- |
| Estradiol | 6 | 3GWK_3 | 6 | 6 | 6 | Streptococcus agalactiae | PUTATIVE UNCHARACTERIZED PROTEIN SAG1039 | 3UUD_EST600B- 2OCF_EST596A- 3OLL_EST600B- 3OLS_EST600B- 4J24_EST600C- 2YJA_EST1550B- |
| Estradiol | 6 | 2XEU_1 | 6 | 6 | 6 | Homo sapiens | RING FINGER PROTEIN 4 | 3UUD_EST600B- 2OCF_EST596A- 3OLL_EST600B- 3OLS_EST600B- 4J24_EST600C- 2YJA_EST1550B- |
| Estradiol | 6 | 2XOV_2 | 6 | 6 | 6 | Escherichia coli | RHOMBOID PROTEASE GLPG | 3UUD_EST600B- 2OCF_EST596A- 3OLL_EST600B- 3OLS_EST600B- 4J24_EST600C- 2YJA_EST1550B- |
| Estradiol | 6 | 4M5B_4 | 6 | 6 | 6 | Caldanaerobacter subterraneus | COBALAMIN BIOSYNTHESIS PROTEIN CBIM | 3UUD_EST600B- 2OCF_EST596A- 3OLL_EST600B- 3OLS_EST600B- 4J24_EST600C- 2YJA_EST1550B- |
| Estradiol | 6 | 3HWU_2 | 5 | 6 | 6 | Cupriavidus pinatubonensis | PUTATIVE DNA-BINDING PROTEIN | 3UUD_EST600B- 2OCF_EST596A- 3OLL_EST600B- 3OLS_EST600B- 4J24_EST600C- |
| Estradiol | 6 | 3CLA_2 | 5 | 6 | 6 | Escherichia coli | TYPE III CHLORAMPHENICOL ACETYLTRANSFERASE | 3UUD_EST600B- 2OCF_EST596A- 3OLS_EST600B- 4J24_EST600C- 2YJA_EST1550B- |
| Estradiol | 6 | 1EW0_5 | 5 | 6 | 6 | Sinorhizobium meliloti | FIXL | 3UUD_EST600B- 2OCF_EST596A- 3OLL_EST600B- 3OLS_EST600B- 4J24_EST600C- |
| Estradiol | 6 | 2OOA_3 | 5 | 6 | 6 | Homo sapiens | E3 UBIQUITIN-PROTEIN LIGASE CBL-B | 3UUD_EST600B- 2OCF_EST596A- 3OLL_EST600B- 3OLS_EST600B- 2YJA_EST1550B- |
| Estradiol | 6 | 4UYB_4 | 5 | 6 | 6 | Homo sapiens | SEC14-LIKE PROTEIN 3 | 3UUD_EST600B- 3OLL_EST600B- 3OLS_EST600B- 4J24_EST600C- 2YJA_EST1550B- |
| Estradiol | 6 | 3N1E_4 | 5 | 6 | 6 | Mus musculus | VACUOLAR PROTEIN SORTING-ASSOCIATED PROTEIN 54 | 3UUD_EST600B- 2OCF_EST596A- 3OLL_EST600B- 3OLS_EST600B- 2YJA_EST1550B- |
| Estradiol | 6 | 1XIO_1 | 5 | 6 | 6 | Nostoc sp. PCC 7120 | ANABAENA SENSORY RHODOPSIN | 3UUD_EST600B- 2OCF_EST596A- 3OLS_EST600B- 4J24_EST600C- 2YJA_EST1550B- |
| Estradiol | 6 | 1V2Z_4 | 4 | 6 | 6 | Thermosynechococcus elongatus | CIRCADIAN CLOCK PROTEIN KAIA HOMOLOG | 3UUD_EST600B- 3OLL_EST600B- 3OLS_EST600B- 4J24_EST600C- |
| Estradiol | 6 | 3O48_3 | 4 | 6 | 6 | Saccharomyces cerevisiae | MITOCHONDRIA FISSION 1 PROTEIN | 3OLL_EST600B- 3OLS_EST600B- 4J24_EST600C- 2YJA_EST1550B- |
| Estradiol | 6 | 3CIM_3 | 4 | 6 | 6 | Synechocystis sp. | CARBON DIOXIDE-CONCENTRATING MECHANISM PROTEIN CCMK HOMOLOG | 3UUD_EST600B- 2OCF_EST596A- 3OLL_EST600B- 3OLS_EST600B- |
| Estradiol | 6 | 3O6P_3 | 4 | 6 | 6 | Enterococcus faecalis | PEPTIDE ABC TRANSPORTER, PEPTIDE-BINDING PROTEIN | 3UUD_EST600B- 3OLL_EST600B- 3OLS_EST600B- 2YJA_EST1550B- |
| Estradiol | 6 | 4HH5_5 | 4 | 6 | 6 | Escherichia coli | PUTATIVE TYPE VI SECRETION PROTEIN | 3UUD_EST600B- 2OCF_EST596A- 3OLS_EST600B- 4J24_EST600C- |
| Estradiol | 6 | 3ZY7_1 | 4 | 6 | 6 | Mus musculus | AP-1 COMPLEX SUBUNIT GAMMA-1 | 2OCF_EST596A- 3OLL_EST600B- 4J24_EST600C- 2YJA_EST1550B- |
| Estradiol | 6 | 1YBK_1 | 4 | 6 | 6 | Staphylothermus marinus | TETRABRACHION | 3UUD_EST600B- 2OCF_EST596A- 3OLL_EST600B- 3OLS_EST600B- |
| Estradiol | 6 | 1SN9_2 | 4 | 6 | 6 | - | TETRAMERIC BETA-BETA-ALPHA MINI-PROTEIN | 2OCF_EST596A- 3OLL_EST600B- 3OLS_EST600B- 2YJA_EST1550B- |
| Estradiol | 6 | 4GOF_5 | 3 | 6 | 6 | Homo sapiens | SMALL GLUTAMINE-RICH TETRATRICOPEPTIDE REPEAT-CONTAINING | 3UUD_EST600B- 2OCF_EST596A- 3OLS_EST600B- |
| Estradiol | 6 | 4DJG_1 | 3 | 6 | 6 | Arabidopsis thaliana | PLECTIN-RELATED PROTEIN | 2OCF_EST596A- 3OLL_EST600B- 3OLS_EST600B- |
| Estradiol | 6 | 4PF3_2 | 3 | 6 | 6 | Homo sapiens | MINERALOCORTICOID RECEPTOR | 2OCF_EST596A- 3OLL_EST600B- 3OLS_EST600B- |
| Estradiol | 6 | 3SK9_5 | 3 | 6 | 6 | Thermus thermophilus | PUTATIVE UNCHARACTERIZED PROTEIN TTHB187 | 3UUD_EST600B- 2OCF_EST596A- 4J24_EST600C- |
| Estradiol | 6 | 2HRA_3 | 3 | 6 | 6 | Saccharomyces cerevisiae | GLUTAMYL-TRNA SYNTHETASE, CYTOPLASMIC | 3UUD_EST600B- 2OCF_EST596A- 3OLS_EST600B- |
| Estradiol | 6 | 1Y6X_1 | 3 | 6 | 6 | Mycobacterium tuberculosis | PHOSPHORIBOSYL-ATP PYROPHOSPHATASE | 2OCF_EST596A- 3OLL_EST600B- 3OLS_EST600B- |
| Estradiol | 6 | 2FEF_2 | 3 | 6 | 6 | Pseudomonas aeruginosa | HYPOTHETICAL PROTEIN PA2201 | 3UUD_EST600B- 2OCF_EST596A- 3OLS_EST600B- |
| Estradiol | 6 | 3OST_1 | 3 | 6 | 6 | Saccharomyces cerevisiae | SERINE/THREONINE-PROTEIN KINASE KCC4 | 2OCF_EST596A- 3OLL_EST600B- 3OLS_EST600B- |
| Estradiol | 6 | 3FGV_1 | 3 | 6 | 6 | Ruegeria pomeroyi | UNCHARACTERIZED PROTEIN WITH FERREDOXIN-LIKE FOLD | 2OCF_EST596A- 3OLS_EST600B- 4J24_EST600C- |
| Estradiol | 6 | 4X86_3 | 3 | 6 | 6 | Homo sapiens | UBIQUITIN-LIKE PROTEIN 4A | 3UUD_EST600B- 2OCF_EST596A- 3OLS_EST600B- |
| Estradiol | 6 | 3ECF_5 | 3 | 6 | 6 | Anabaena variabilis | NTF2-LIKE PROTEIN | 3OLL_EST600B- 3OLS_EST600B- 4J24_EST600C- |
| Estradiol | 6 | 2EWT_2 | 3 | 6 | 6 | Streptomyces coelicolor | PUTATIVE DNA-BINDING PROTEIN | 2OCF_EST596A- 3OLL_EST600B- 3OLS_EST600B- |
| Estradiol | 6 | 3LHC_2 | 2 | 6 | 6 | Nostoc ellipsosporum | CYANOVIRIN-N | 2OCF_EST596A- 3OLS_EST600B- |
| Estradiol | 6 | 2P09_3 | 2 | 6 | 6 | unidentified | A NON-BIOLOGICAL ATP BINDING PROTEIN WITH TWO MUTATIONS | 3OLL_EST600B- 3OLS_EST600B- |
| Estradiol | 6 | 4LPQ_1 | 2 | 6 | 6 | Xylanimonas cellulosilytica | ERFK/YBIS/YCFS/YNHG FAMILY PROTEIN | 3UUD_EST600B- 3OLS_EST600B- |
| Estradiol | 6 | 2MLT_1 | 2 | 5 | 6 | Apis mellifera | MELITTIN | 2OCF_EST596A- 3OLS_EST600B- |
| Estradiol | 6 | 2O38_2 | 2 | 6 | 6 | Rhodopseudomonas palustris | HYPOTHETICAL PROTEIN | 2OCF_EST596A- 3OLS_EST600B- |
| Estradiol | 6 | 1P9H_5 | 2 | 5 | 6 | Yersinia enterocolitica | INVASIN | 2OCF_EST596A- 3OLS_EST600B- |
| Estradiol | 6 | 3KFF_5 | 2 | 6 | 6 | Mus musculus | MAJOR URINARY PROTEIN 4 | 3OLS_EST600B- 4J24_EST600C- |
| Estradiol | 6 | 3LK7_3 | 2 | 6 | 6 | Streptococcus agalactiae | UDP-N-ACETYLMURAMOYLALANINE--D-GLUTAMATE LIGASE | 2OCF_EST596A- 2YJA_EST1550B- |
| Estradiol | 6 | 1Q8B_2 | 2 | 5 | 6 | Bacillus subtilis | PROTEIN YJCS | 3UUD_EST600B- 3OLS_EST600B- |
| Estradiol | 6 | 1PZX_4 | 2 | 5 | 6 | Geobacillus stearothermophilus | HYPOTHETICAL PROTEIN APC36103 | 3UUD_EST600B- 3OLS_EST600B- |
| Estradiol | 6 | 4LLE_4 | 2 | 6 | 6 | Pseudomonas aeruginosa | PROBABLE TWO-COMPONENT SENSOR | 2OCF_EST596A- 3OLS_EST600B- |
| Estradiol | 6 | 1EZ3_3 | 2 | 6 | 6 | Rattus norvegicus | SYNTAXIN-1A | 3UUD_EST600B- 2OCF_EST596A- |
| Estradiol | 6 | 2RFF_4 | 2 | 6 | 6 | Sulfolobus solfataricus | PUTATIVE NUCLEOTIDYLTRANSFERASE | 3OLS_EST600B- 2YJA_EST1550B- |
| Sirolimus | 6 | 4DRI_1 | 4 | 4 | 5 | Homo sapiens | PEPTIDYL-PROLYL CIS-TRANS ISOMERASE FKBP5 | 2DG4_RAP501A- 4DRI_RAP201A- 1FKB_RAP108A- 1FAP_RAP108A- |
| Progesterone | 6 | 3HWU_2 | 6 | 6 | 6 | Cupriavidus pinatubonensis | PUTATIVE DNA-BINDING PROTEIN | 4FN9_STR301A- 1A28_STR1A- 2AA6_STR401A- 4LTW_STR301A- 1YA3_STR3001C- 2AA5_STR301A- |
| Progesterone | 6 | 4TW3_5 | 6 | 6 | 6 | Prochlorococcus marinus | ALDEHYDE DECARBONYLASE | 4FN9_STR301A- 1A28_STR1A- 2AA6_STR401A- 4LTW_STR301A- 1YA3_STR3001C- 2AA5_STR301A- |
| Progesterone | 6 | 1H2S_7 | 6 | 6 | 6 | Natronomonas pharaonis | SENSORY RHODOPSIN II | 4FN9_STR301A- 1A28_STR1A- 2AA6_STR401A- 4LTW_STR301A- 1YA3_STR3001C- 2AA5_STR301A- |
| Progesterone | 6 | 3CLA_2 | 6 | 6 | 6 | Escherichia coli | TYPE III CHLORAMPHENICOL ACETYLTRANSFERASE | 4FN9_STR301A- 1A28_STR1A- 2AA6_STR401A- 4LTW_STR301A- 1YA3_STR3001C- 2AA5_STR301A- |
| Progesterone | 6 | 1YBK_2 | 6 | 6 | 6 | Staphylothermus marinus | TETRABRACHION | 4FN9_STR301A- 1A28_STR1A- 2AA6_STR401A- 4LTW_STR301A- 1YA3_STR3001C- 2AA5_STR301A- |
| Progesterone | 6 | 1H2S_6 | 6 | 6 | 6 | Natronomonas pharaonis | SENSORY RHODOPSIN II | 4FN9_STR301A- 1A28_STR1A- 2AA6_STR401A- 4LTW_STR301A- 1YA3_STR3001C- 2AA5_STR301A- |
| Progesterone | 6 | 4U9B_3 | 6 | 6 | 6 | Shewanella oneidensis | NO-BINDING HEME-DEPENDENT SENSOR PROTEIN | 4FN9_STR301A- 1A28_STR1A- 2AA6_STR401A- 4LTW_STR301A- 1YA3_STR3001C- 2AA5_STR301A- |
| Progesterone | 6 | 3S0A_1 | 6 | 6 | 6 | Apis mellifera | OBP14 | 4FN9_STR301A- 1A28_STR1A- 2AA6_STR401A- 4LTW_STR301A- 1YA3_STR3001C- 2AA5_STR301A- |
| Progesterone | 6 | 4PF3_2 | 6 | 6 | 6 | Homo sapiens | MINERALOCORTICOID RECEPTOR | 4FN9_STR301A- 1A28_STR1A- 2AA6_STR401A- 4LTW_STR301A- 1YA3_STR3001C- 2AA5_STR301A- |
| Progesterone | 6 | 4I66_2 | 6 | 6 | 6 | Haliangium ochraceum | UNCHARACTERIZED PROTEIN HOCH_4089 | 4FN9_STR301A- 1A28_STR1A- 2AA6_STR401A- 4LTW_STR301A- 1YA3_STR3001C- 2AA5_STR301A- |
| Progesterone | 6 | 1Y6X_1 | 6 | 6 | 6 | Mycobacterium tuberculosis | PHOSPHORIBOSYL-ATP PYROPHOSPHATASE | 4FN9_STR301A- 1A28_STR1A- 2AA6_STR401A- 4LTW_STR301A- 1YA3_STR3001C- 2AA5_STR301A- |
| Progesterone | 6 | 1XSV_3 | 6 | 6 | 6 | Staphylococcus aureus | HYPOTHETICAL UPF0122 PROTEIN SAV1236 | 4FN9_STR301A- 1A28_STR1A- 2AA6_STR401A- 4LTW_STR301A- 1YA3_STR3001C- 2AA5_STR301A- |
| Progesterone | 6 | 4UYB_4 | 6 | 6 | 6 | Homo sapiens | SEC14-LIKE PROTEIN 3 | 4FN9_STR301A- 1A28_STR1A- 2AA6_STR401A- 4LTW_STR301A- 1YA3_STR3001C- 2AA5_STR301A- |
| Progesterone | 6 | 3RFI_2 | 6 | 6 | 6 | Solanum tuberosum | ASP | 4FN9_STR301A- 1A28_STR1A- 2AA6_STR401A- 4LTW_STR301A- 1YA3_STR3001C- 2AA5_STR301A- |
| Progesterone | 6 | 4IGV_1 | 6 | 6 | 6 | Actinidia deliciosa | KIROLA | 4FN9_STR301A- 1A28_STR1A- 2AA6_STR401A- 4LTW_STR301A- 1YA3_STR3001C- 2AA5_STR301A- |
| Progesterone | 6 | 2DWK_5 | 6 | 6 | 6 | Mus musculus | PROTEIN RUFY3 | 4FN9_STR301A- 1A28_STR1A- 2AA6_STR401A- 4LTW_STR301A- 1YA3_STR3001C- 2AA5_STR301A- |
| Progesterone | 6 | 3GWK_3 | 6 | 6 | 6 | Streptococcus agalactiae | PUTATIVE UNCHARACTERIZED PROTEIN SAG1039 | 4FN9_STR301A- 1A28_STR1A- 2AA6_STR401A- 4LTW_STR301A- 1YA3_STR3001C- 2AA5_STR301A- |
| Progesterone | 6 | 3N1E_4 | 6 | 6 | 6 | Mus musculus | VACUOLAR PROTEIN SORTING-ASSOCIATED PROTEIN 54 | 4FN9_STR301A- 1A28_STR1A- 2AA6_STR401A- 4LTW_STR301A- 1YA3_STR3001C- 2AA5_STR301A- |
| Progesterone | 6 | 3ECF_5 | 6 | 6 | 6 | Anabaena variabilis | NTF2-LIKE PROTEIN | 4FN9_STR301A- 1A28_STR1A- 2AA6_STR401A- 4LTW_STR301A- 1YA3_STR3001C- 2AA5_STR301A- |
| Progesterone | 6 | 2XOV_2 | 6 | 6 | 6 | Escherichia coli | RHOMBOID PROTEASE GLPG | 4FN9_STR301A- 1A28_STR1A- 2AA6_STR401A- 4LTW_STR301A- 1YA3_STR3001C- 2AA5_STR301A- |
| Progesterone | 6 | 1SN9_2 | 6 | 6 | 6 | - | TETRAMERIC BETA-BETA-ALPHA MINI-PROTEIN | 4FN9_STR301A- 1A28_STR1A- 2AA6_STR401A- 4LTW_STR301A- 1YA3_STR3001C- 2AA5_STR301A- |
| Progesterone | 6 | 1XIO_1 | 6 | 6 | 6 | Nostoc sp. PCC 7120 | ANABAENA SENSORY RHODOPSIN | 4FN9_STR301A- 1A28_STR1A- 2AA6_STR401A- 4LTW_STR301A- 1YA3_STR3001C- 2AA5_STR301A- |
| Progesterone | 6 | 1WV3_1 | 5 | 6 | 6 | Staphylococcus aureus | SIMILAR TO DNA SEGREGATION ATPASE AND RELATED | 4FN9_STR301A- 1A28_STR1A- 2AA6_STR401A- 1YA3_STR3001C- 2AA5_STR301A- |
| Progesterone | 6 | 3FSS_5 | 5 | 6 | 6 | Saccharomyces cerevisiae | HISTONE CHAPERONE RTT106 | 4FN9_STR301A- 1A28_STR1A- 4LTW_STR301A- 1YA3_STR3001C- 2AA5_STR301A- |
| Progesterone | 6 | 4PND_2 | 5 | 6 | 6 | synthetic construct | CC-PENT_VARIANT | 4FN9_STR301A- 1A28_STR1A- 4LTW_STR301A- 1YA3_STR3001C- 2AA5_STR301A- |
| Progesterone | 6 | 1EW0_5 | 5 | 6 | 6 | Sinorhizobium meliloti | FIXL | 4FN9_STR301A- 1A28_STR1A- 2AA6_STR401A- 1YA3_STR3001C- 2AA5_STR301A- |
| Progesterone | 6 | 2O71_1 | 5 | 6 | 6 | Homo sapiens | DEATH DOMAIN-CONTAINING PROTEIN CRADD | 4FN9_STR301A- 1A28_STR1A- 4LTW_STR301A- 1YA3_STR3001C- 2AA5_STR301A- |
| Progesterone | 6 | 3PCV_1 | 5 | 6 | 6 | Homo sapiens | LEUKOTRIENE C4 SYNTHASE | 4FN9_STR301A- 1A28_STR1A- 4LTW_STR301A- 1YA3_STR3001C- 2AA5_STR301A- |
| Progesterone | 6 | 1M0K_1 | 5 | 6 | 6 | Halobacterium salinarum | BACTERIORHODOPSIN | 4FN9_STR301A- 1A28_STR1A- 4LTW_STR301A- 1YA3_STR3001C- 2AA5_STR301A- |
| Progesterone | 6 | 3FGV_1 | 5 | 6 | 6 | Ruegeria pomeroyi | UNCHARACTERIZED PROTEIN WITH FERREDOXIN-LIKE FOLD | 4FN9_STR301A- 1A28_STR1A- 4LTW_STR301A- 1YA3_STR3001C- 2AA5_STR301A- |
| Progesterone | 6 | 4TW3_1 | 5 | 6 | 6 | Prochlorococcus marinus | ALDEHYDE DECARBONYLASE | 4FN9_STR301A- 1A28_STR1A- 4LTW_STR301A- 1YA3_STR3001C- 2AA5_STR301A- |
| Progesterone | 6 | 4GOF_5 | 4 | 6 | 6 | Homo sapiens | SMALL GLUTAMINE-RICH TETRATRICOPEPTIDE REPEAT-CONTAINING | 4FN9_STR301A- 1A28_STR1A- 1YA3_STR3001C- 2AA5_STR301A- |
| Progesterone | 6 | 4PNO_1 | 4 | 6 | 6 | Escherichia coli | RNA-BINDING PROTEIN HFQ | 4FN9_STR301A- 1A28_STR1A- 2AA6_STR401A- 1YA3_STR3001C- |
| Progesterone | 6 | 2XEU_1 | 4 | 6 | 6 | Homo sapiens | RING FINGER PROTEIN 4 | 4FN9_STR301A- 1A28_STR1A- 1YA3_STR3001C- 2AA5_STR301A- |
| Progesterone | 6 | 3O6P_3 | 4 | 6 | 6 | Enterococcus faecalis | PEPTIDE ABC TRANSPORTER, PEPTIDE-BINDING PROTEIN | 4FN9_STR301A- 1A28_STR1A- 1YA3_STR3001C- 2AA5_STR301A- |
| Progesterone | 6 | 2EWT_2 | 4 | 6 | 6 | Streptomyces coelicolor | PUTATIVE DNA-BINDING PROTEIN | 4FN9_STR301A- 1A28_STR1A- 1YA3_STR3001C- 2AA5_STR301A- |
| Progesterone | 6 | 4KV2_1 | 4 | 6 | 6 | Mycobacterium tuberculosis | ESX-1 SECRETION SYSTEM PROTEIN ECCD1 | 4FN9_STR301A- 1A28_STR1A- 4LTW_STR301A- 2AA5_STR301A- |
| Progesterone | 6 | 2J97_1 | 4 | 6 | 6 | Human coronavirus 229E | REPLICASE POLYPROTEIN 1AB | 1A28_STR1A- 2AA6_STR401A- 4LTW_STR301A- 2AA5_STR301A- |
| Progesterone | 6 | 3B2Y_2 | 3 | 5 | 6 | Shewanella denitrificans | METALLOPEPTIDASE CONTAINING CO-CATALYTIC METALLOACTIVE | 4FN9_STR301A- 1A28_STR1A- 2AA6_STR401A- |
| Progesterone | 6 | 3F6O_4 | 3 | 6 | 6 | Rhodococcus jostii | PROBABLE TRANSCRIPTIONAL REGULATOR, ARSR FAMILY | 4FN9_STR301A- 2AA6_STR401A- 2AA5_STR301A- |
| Progesterone | 6 | 3LDC_1 | 3 | 6 | 6 | Methanothermobacter thermautotrophicus | CALCIUM-GATED POTASSIUM CHANNEL MTHK | 1A28_STR1A- 4LTW_STR301A- 1YA3_STR3001C- |
| Progesterone | 6 | 3O48_3 | 3 | 6 | 6 | Saccharomyces cerevisiae | MITOCHONDRIA FISSION 1 PROTEIN | 4FN9_STR301A- 1A28_STR1A- 2AA5_STR301A- |
| Progesterone | 6 | 2P09_3 | 3 | 6 | 6 | unidentified | A NON-BIOLOGICAL ATP BINDING PROTEIN WITH TWO MUTATIONS | 1A28_STR1A- 2AA6_STR401A- 1YA3_STR3001C- |
| Progesterone | 6 | 3H51_2 | 3 | 6 | 6 | Xanthomonas campestris | PUTATIVE CALCIUM/CALMODULIN DEPENDENT PROTEIN KINASE II | 4FN9_STR301A- 1A28_STR1A- 2AA5_STR301A- |
| Progesterone | 6 | 2OOA_3 | 3 | 6 | 6 | Homo sapiens | E3 UBIQUITIN-PROTEIN LIGASE CBL-B | 1A28_STR1A- 1YA3_STR3001C- 2AA5_STR301A- |
| Progesterone | 6 | 2IAY_1 | 3 | 6 | 6 | Lactobacillus plantarum | HYPOTHETICAL PROTEIN | 4FN9_STR301A- 1YA3_STR3001C- 2AA5_STR301A- |
| Progesterone | 6 | 3KBG_2 | 3 | 5 | 6 | Thermoplasma acidophilum | 30S RIBOSOMAL PROTEIN S4E | 4FN9_STR301A- 1A28_STR1A- 4LTW_STR301A- |
| Progesterone | 6 | 1TUK_2 | 3 | 6 | 6 | Triticum aestivum | NONSPECIFIC LIPID-TRANSFER PROTEIN 2G | 4FN9_STR301A- 1A28_STR1A- 2AA5_STR301A- |
| Progesterone | 6 | 3LK7_3 | 3 | 6 | 6 | Streptococcus agalactiae | UDP-N-ACETYLMURAMOYLALANINE--D-GLUTAMATE LIGASE | 1A28_STR1A- 1YA3_STR3001C- 2AA5_STR301A- |
| Progesterone | 6 | 4FTF_1 | 3 | 3 | 6 | Vibrio cholerae | ALTERNATE SECRETIN PATHWAY SUBUNIT S (VC395_1821, VC1703) | 4FN9_STR301A- 1YA3_STR3001C- 2AA5_STR301A- |
| Progesterone | 6 | 1PZX_4 | 3 | 6 | 6 | Geobacillus stearothermophilus | HYPOTHETICAL PROTEIN APC36103 | 1A28_STR1A- 4LTW_STR301A- 2AA5_STR301A- |
| Progesterone | 6 | 1Z0P_1 | 3 | 6 | 6 | Streptococcus pyogenes | HYPOTHETICAL PROTEIN SPY1572 | 4FN9_STR301A- 4LTW_STR301A- 1YA3_STR3001C- |
| Progesterone | 6 | 4LLE_4 | 3 | 5 | 6 | Pseudomonas aeruginosa | PROBABLE TWO-COMPONENT SENSOR | 4FN9_STR301A- 1YA3_STR3001C- 2AA5_STR301A- |
| Progesterone | 6 | 1V2Z_4 | 2 | 6 | 6 | Thermosynechococcus elongatus | CIRCADIAN CLOCK PROTEIN KAIA HOMOLOG | 2AA6_STR401A- 1YA3_STR3001C- |
| Progesterone | 6 | 2R2Z_1 | 2 | 6 | 6 | Enterococcus faecalis | HEMOLYSIN | 1A28_STR1A- 2AA5_STR301A- |
| Progesterone | 6 | 3WMI_3 | 2 | 6 | 6 | Equus caballus | EIAV GP45 WILD TYPE | 4FN9_STR301A- 2AA5_STR301A- |
| Progesterone | 6 | 3K3C_4 | 2 | 6 | 6 | Mycobacterium tuberculosis | PROTEIN RV1364C/MT1410 | 1A28_STR1A- 1YA3_STR3001C- |
| Progesterone | 6 | 2FEF_2 | 2 | 6 | 6 | Pseudomonas aeruginosa | HYPOTHETICAL PROTEIN PA2201 | 1A28_STR1A- 2AA5_STR301A- |
| Progesterone | 6 | 1VMG_2 | 2 | 5 | 6 | Sulfolobus solfataricus | HYPOTHETICAL PROTEIN SSO3215 | 4LTW_STR301A- 1YA3_STR3001C- |
| Progesterone | 6 | 3LJW_2 | 2 | 6 | 6 | Homo sapiens | PROTEIN POLYBROMO-1 | 1A28_STR1A- 4LTW_STR301A- |
| Progesterone | 6 | 1Y9L_1 | 2 | 5 | 6 | Shigella flexneri | LIPOPROTEIN MXIM | 4FN9_STR301A- 4LTW_STR301A- |
| Progesterone | 6 | 1KS9_2 | 2 | 5 | 6 | Escherichia coli | 2-DEHYDROPANTOATE 2-REDUCTASE | 2AA6_STR401A- 2AA5_STR301A- |
| Progesterone | 6 | 4HH5_5 | 2 | 6 | 6 | Escherichia coli | PUTATIVE TYPE VI SECRETION PROTEIN | 4FN9_STR301A- 1YA3_STR3001C- |
| Progesterone | 6 | 2RIJ_2 | 2 | 6 | 6 | Campylobacter jejuni | PUTATIVE 2,3,4,5-TETRAHYDROPYRIDINE-2-CARBOXYLATE N- | 1A28_STR1A- 2AA5_STR301A- |
| Progesterone | 6 | 3ZY7_1 | 2 | 6 | 6 | Mus musculus | AP-1 COMPLEX SUBUNIT GAMMA-1 | 1YA3_STR3001C- 2AA5_STR301A- |
| Progesterone | 6 | 1YBK_1 | 2 | 6 | 6 | Staphylothermus marinus | TETRABRACHION | 1A28_STR1A- 2AA5_STR301A- |
| Progesterone | 6 | 4M5B_4 | 2 | 5 | 6 | Caldanaerobacter subterraneus | COBALAMIN BIOSYNTHESIS PROTEIN CBIM | 4FN9_STR301A- 1A28_STR1A- |
| Progesterone | 6 | 2RFF_4 | 2 | 6 | 6 | Sulfolobus solfataricus | PUTATIVE NUCLEOTIDYLTRANSFERASE | 4FN9_STR301A- 1A28_STR1A- |
| Progesterone | 6 | 2P6V_4 | 2 | 6 | 6 | Homo sapiens | TRANSCRIPTION INITIATION FACTOR TFIID SUBUNIT 4 | 4FN9_STR301A- 1YA3_STR3001C- |
| Liothyronine | 6 | 1H2S_7 | 6 | 6 | 6 | Natronomonas pharaonis | SENSORY RHODOPSIN II | 3GWS_T3500X- 2H79_T31A- 1XZX_T3500X- 4LNW_T3501A- 1BSX_T31A- 4LNX_T3502A- |
| Liothyronine | 6 | 2J7Q_5 | 6 | 6 | 6 | Murid herpesvirus 1 | UBIQUITIN | 3GWS_T3500X- 2H79_T31A- 1XZX_T3500X- 4LNW_T3501A- 1BSX_T31A- 4LNX_T3502A- |
| Liothyronine | 6 | 2DWK_5 | 4 | 6 | 6 | Mus musculus | PROTEIN RUFY3 | 2H79_T31A- 1XZX_T3500X- 1BSX_T31A- 4LNX_T3502A- |
| Liothyronine | 6 | 3LDC_1 | 4 | 5 | 6 | Methanothermobacter thermautotrophicus | CALCIUM-GATED POTASSIUM CHANNEL MTHK | 2H79_T31A- 1XZX_T3500X- 1BSX_T31A- 4LNX_T3502A- |
| Liothyronine | 6 | 3GWK_3 | 4 | 6 | 6 | Streptococcus agalactiae | PUTATIVE UNCHARACTERIZED PROTEIN SAG1039 | 2H79_T31A- 1XZX_T3500X- 1BSX_T31A- 4LNX_T3502A- |
| Liothyronine | 6 | 3FSS_5 | 4 | 6 | 6 | Saccharomyces cerevisiae | HISTONE CHAPERONE RTT106 | 2H79_T31A- 1XZX_T3500X- 1BSX_T31A- 4LNX_T3502A- |
| Liothyronine | 6 | 4PZJ_2 | 3 | 5 | 6 | Eggerthella lenta | TRANSCRIPTIONAL REGULATOR, LYSR FAMILY | 2H79_T31A- 1XZX_T3500X- 1BSX_T31A- |
| Liothyronine | 6 | 1VMG_2 | 3 | 6 | 6 | Sulfolobus solfataricus | HYPOTHETICAL PROTEIN SSO3215 | 2H79_T31A- 1XZX_T3500X- 4LNX_T3502A- |
| Liothyronine | 6 | 4U9B_3 | 3 | 6 | 6 | Shewanella oneidensis | NO-BINDING HEME-DEPENDENT SENSOR PROTEIN | 2H79_T31A- 1XZX_T3500X- 4LNX_T3502A- |
| Liothyronine | 6 | 3S0A_1 | 3 | 5 | 6 | Apis mellifera | OBP14 | 2H79_T31A- 1XZX_T3500X- 4LNX_T3502A- |
| Liothyronine | 6 | 1G8Q_1 | 3 | 6 | 6 | Homo sapiens | CD81 ANTIGEN, EXTRACELLULAR DOMAIN | 3GWS_T3500X- 2H79_T31A- 1BSX_T31A- |
| Liothyronine | 6 | 1Y6X_1 | 3 | 6 | 6 | Mycobacterium tuberculosis | PHOSPHORIBOSYL-ATP PYROPHOSPHATASE | 2H79_T31A- 1XZX_T3500X- 4LNX_T3502A- |
| Liothyronine | 6 | 1SN9_2 | 3 | 6 | 6 | - | TETRAMERIC BETA-BETA-ALPHA MINI-PROTEIN | 2H79_T31A- 1XZX_T3500X- 4LNX_T3502A- |
| Liothyronine | 6 | 4UYB_4 | 3 | 5 | 6 | Homo sapiens | SEC14-LIKE PROTEIN 3 | 1XZX_T3500X- 1BSX_T31A- 4LNX_T3502A- |
| Liothyronine | 6 | 4I2L_1 | 2 | 4 | 6 | Human immunodeficiency virus 1 | GP41 | 1BSX_T31A- 4LNX_T3502A- |
| Liothyronine | 6 | 4PND_2 | 2 | 6 | 6 | synthetic construct | CC-PENT_VARIANT | 1XZX_T3500X- 4LNW_T3501A- |
| Liothyronine | 6 | 4A20_2 | 2 | 6 | 6 | Saccharomyces cerevisiae | UBIQUITIN-LIKE PROTEIN MDY2 | 3GWS_T3500X- 1BSX_T31A- |
| Liothyronine | 6 | 2EH3_1 | 2 | 5 | 6 | Aquifex aeolicus | TRANSCRIPTIONAL REGULATOR | 3GWS_T3500X- 2H79_T31A- |
| Liothyronine | 6 | 2Z1C_3 | 2 | 6 | 6 | Thermococcus kodakarensis | HYDROGENASE EXPRESSION/FORMATION PROTEIN HYPC | 2H79_T31A- 1BSX_T31A- |
| Aminocaproic Acid | 5 | 3Q8J_1 | 4 | 5 | 5 | Asteropus | ASTEROPSIN A | 1CEA_ACA90A- 3KIV_ACA100A- 1HPK_ACA80A- 1PK2_ACA91A- |
| Aminocaproic Acid | 5 | 1FV1_7 | 4 | 4 | 4 | Homo sapiens | MAJOR HISTOCOMPATIBILITY COMPLEX ALPHA CHAIN | 1CEA_ACA90A- 3KIV_ACA100A- 1HPK_ACA80A- 2PK4_ACA100A- |
| Aminocaproic Acid | 5 | 3D4E_2 | 4 | 5 | 5 | Streptococcus mutans | PUTATIVE BETA-LACTAMASE INHIBITOR PROTEIN | 1CEA_ACA90A- 3KIV_ACA100A- 1HPK_ACA80A- 2PK4_ACA100A- |
| Aminocaproic Acid | 5 | 3MCX_4 | 4 | 4 | 5 | Bacteroides thetaiotaomicron | SUSD SUPERFAMILY PROTEIN | 1CEA_ACA90A- 3KIV_ACA100A- 1HPK_ACA80A- 2PK4_ACA100A- |
| Aminocaproic Acid | 5 | 2BTI_4 | 3 | 5 | 5 | Yersinia enterocolitica | CARBON STORAGE REGULATOR HOMOLOG | 3KIV_ACA100A- 1HPK_ACA80A- 2PK4_ACA100A- |
| Aminocaproic Acid | 5 | 2CMP_1 | 3 | 4 | 5 | Bacillus phage SF6 | TERMINASE SMALL SUBUNIT | 1CEA_ACA90A- 3KIV_ACA100A- 2PK4_ACA100A- |
| Aminocaproic Acid | 5 | 1SAU_2 | 3 | 4 | 5 | Archaeoglobus fulgidus | SULFITE REDUCTASE, DESULFOVIRIDIN-TYPE SUBUNIT GAMMA | 3KIV_ACA100A- 1HPK_ACA80A- 1PK2_ACA91A- |
| Aminocaproic Acid | 5 | 3QRL_4 | 3 | 3 | 3 | Saccharomyces cerevisiae | TRANSCRIPTION INITIATION FACTOR TFIID SUBUNIT 14 | 1CEA_ACA90A- 3KIV_ACA100A- 2PK4_ACA100A- |
| Aminocaproic Acid | 5 | 1I71_2 | 3 | 5 | 5 | Homo sapiens | APOLIPOPROTEIN(A) | 3KIV_ACA100A- 1HPK_ACA80A- 2PK4_ACA100A- |
| Aminocaproic Acid | 5 | 4WHI_3 | 3 | 5 | 5 | Mycobacterium tuberculosis | BETA-LACTAMASE | 1CEA_ACA90A- 3KIV_ACA100A- 1HPK_ACA80A- |
| Aminocaproic Acid | 5 | 3F2E_3 | 3 | 4 | 5 | Rudivirus | SIRV COAT PROTEIN | 1CEA_ACA90A- 1HPK_ACA80A- 1PK2_ACA91A- |
| Aminocaproic Acid | 5 | 2VB1_4 | 3 | 5 | 5 | Gallus gallus | LYSOZYME C | 3KIV_ACA100A- 2PK4_ACA100A- 1PK2_ACA91A- |
| Aminocaproic Acid | 5 | 3MVU_2 | 2 | 2 | 4 | Ruegeria sp. TM1040 | TENA FAMILY TRANSCRIPTIONAL REGULATOR | 1HPK_ACA80A- 1PK2_ACA91A- |
| Aminocaproic Acid | 5 | 1WHZ_1 | 2 | 5 | 5 | Thermus thermophilus | HYPOTHETICAL PROTEIN | 3KIV_ACA100A- 2PK4_ACA100A- |
| Aminocaproic Acid | 5 | 1DYP_4 | 2 | 5 | 5 | Pseudoalteromonas carrageenovora | KAPPA-CARRAGEENASE | 1CEA_ACA90A- 1PK2_ACA91A- |
| Aminocaproic Acid | 5 | 1LO7_3 | 2 | 3 | 5 | Pseudomonas sp. CBS3 | 4-HYDROXYBENZOYL-COA THIOESTERASE | 1HPK_ACA80A- 1PK2_ACA91A- |
| Aminocaproic Acid | 5 | 3HKM_5 | 2 | 5 | 5 | Oryza sativa | OS03G0854200 PROTEIN | 1CEA_ACA90A- 1HPK_ACA80A- |
| Aminocaproic Acid | 5 | 3NOJ_3 | 2 | 2 | 5 | Pseudomonas putida | 4-CARBOXY-4-HYDROXY-2-OXOADIPATE ALDOLASE/OXALOACETATE | 1HPK_ACA80A- 1PK2_ACA91A- |
| Aminocaproic Acid | 5 | 3ZHI_2 | 2 | 2 | 3 | Lactococcus phage TP901-1 | CI | 1HPK_ACA80A- 1PK2_ACA91A- |
| Aminocaproic Acid | 5 | 2C2U_2 | 2 | 4 | 4 | Deinococcus radiodurans | DNA-BINDING STRESS RESPONSE PROTEIN | 1HPK_ACA80A- 1PK2_ACA91A- |
| Aminocaproic Acid | 5 | 4LQ6_4 | 2 | 2 | 5 | Mycobacterium tuberculosis | N-ACETYMURAMYL-L-ALANINE AMIDASE-RELATED PROTEIN | 3KIV_ACA100A- 1HPK_ACA80A- |
| Aminocaproic Acid | 5 | 2ERL_1 | 2 | 2 | 2 | Euplotes raikovi | MATING PHEROMONE ER-1 | 1HPK_ACA80A- 1PK2_ACA91A- |
| Aminocaproic Acid | 5 | 3BLN_2 | 2 | 2 | 3 | Bacillus cereus | ACETYLTRANSFERASE GNAT FAMILY | 1HPK_ACA80A- 1PK2_ACA91A- |
| Aminocaproic Acid | 5 | 3HMS_3 | 2 | 2 | 2 | Homo sapiens | HEPATOCYTE GROWTH FACTOR | 3KIV_ACA100A- 2PK4_ACA100A- |
| Aminocaproic Acid | 5 | 3V1A_2 | 2 | 4 | 5 | synthetic construct | COMPUTATIONAL DESIGN, MID1-APO1 | 1HPK_ACA80A- 2PK4_ACA100A- |
| Aminocaproic Acid | 5 | 2WJR_5 | 2 | 2 | 3 | Escherichia coli | PROBABLE N-ACETYLNEURAMINIC ACID OUTER MEMBRANE CHANNEL | 1HPK_ACA80A- 1PK2_ACA91A- |
| Aminocaproic Acid | 5 | 4Q2W_3 | 2 | 5 | 5 | Streptococcus pneumoniae | PUTATIVE ENDO-BETA-N-ACETYLGLUCOSAMINIDASE | 1CEA_ACA90A- 1HPK_ACA80A- |
| Aminocaproic Acid | 5 | 2ZVY_5 | 2 | 5 | 5 | Salmonella enterica | CHEMOTAXIS PROTEIN MOTB | 3KIV_ACA100A- 2PK4_ACA100A- |
| Aminocaproic Acid | 5 | 1C1Y_5 | 2 | 3 | 5 | Homo sapiens | RAS-RELATED PROTEIN RAP-1A | 3KIV_ACA100A- 2PK4_ACA100A- |
| Aminocaproic Acid | 5 | 3LBW_2 | 2 | 2 | 3 | Influenza A virus | M2 PROTEIN | 3KIV_ACA100A- 2PK4_ACA100A- |
| Aminocaproic Acid | 5 | 1TFE_7 | 2 | 3 | 4 | Thermus thermophilus | ELONGATION FACTOR TS | 3KIV_ACA100A- 2PK4_ACA100A- |
| Aminocaproic Acid | 5 | 2GZQ_2 | 2 | 2 | 4 | Plasmodium vivax | PHOSPHATIDYLETHANOLAMINE-BINDING PROTEIN | 1HPK_ACA80A- 1PK2_ACA91A- |
| Aminocaproic Acid | 5 | 3ONH_1 | 2 | 3 | 5 | Saccharomyces cerevisiae | UBIQUITIN-ACTIVATING ENZYME E1-LIKE | 3KIV_ACA100A- 1HPK_ACA80A- |
| Aminocaproic Acid | 5 | 3W0E_2 | 2 | 2 | 5 | Aspergillus fumigatus | ELASTASE INHIBITOR AFUEI | 1CEA_ACA90A- 2PK4_ACA100A- |
| Aminocaproic Acid | 5 | 1NWZ_2 | 2 | 4 | 5 | Halorhodospira halophila | PHOTOACTIVE YELLOW PROTEIN | 1HPK_ACA80A- 1PK2_ACA91A- |
| Aminocaproic Acid | 5 | 2X9O_3 | 2 | 3 | 5 | Synechococcus sp. WH 8020 | 15,16-DIHYDROBILIVERDIN-FERREDOXIN OXIDOREDUCTASE | 1HPK_ACA80A- 1PK2_ACA91A- |
| Aminocaproic Acid | 5 | 1XAK_1 | 2 | 3 | 5 | Severe acute respiratory syndrome-related coronavirus | SARS ORF7A ACCESSORY PROTEIN | 1CEA_ACA90A- 3KIV_ACA100A- |
| Aminocaproic Acid | 5 | 2IVY_7 | 2 | 4 | 4 | Sulfolobus solfataricus | HYPOTHETICAL PROTEIN SSO1404 | 3KIV_ACA100A- 2PK4_ACA100A- |
| Dasatinib | 5 | 4A20_2 | 4 | 5 | 5 | Saccharomyces cerevisiae | UBIQUITIN-LIKE PROTEIN MDY2 | 2GQG_1N1501A- 3G5D_1N11A- 2ZVA_1N1513A- 3QLG_1N1601AA |
| Dasatinib | 5 | 2XGV_4 | 3 | 5 | 5 | Microcebus murinus | PSIV CAPSID N-TERMINAL DOMAIN | 3G5D_1N11A- 2ZVA_1N1513A- 3QLG_1N1601AA |
| Dasatinib | 5 | 4U12_2 | 3 | 4 | 5 | Helicobacter pylori | UNCHARACTERIZED PROTEIN HP0242 | 2GQG_1N1501A- 3G5D_1N11A- 2ZVA_1N1513A- |
| Dasatinib | 5 | 4PND_2 | 3 | 5 | 5 | synthetic construct | CC-PENT_VARIANT | 3G5D_1N11A- 2ZVA_1N1513A- 3QLG_1N1601AA |
| Dasatinib | 5 | 1H2S_7 | 3 | 4 | 5 | Natronomonas pharaonis | SENSORY RHODOPSIN II | 3G5D_1N11A- 2ZVA_1N1513A- 3QLG_1N1601AA |
| Dasatinib | 5 | 1JO0_2 | 2 | 5 | 5 | Haemophilus influenzae | HYPOTHETICAL PROTEIN HI1333 | 3G5D_1N11A- 2ZVA_1N1513A- |
| Dasatinib | 5 | 3G0O_2 | 2 | 3 | 5 | Salmonella enterica | 3-HYDROXYISOBUTYRATE DEHYDROGENASE | 3G5D_1N11A- 2ZVA_1N1513A- |
| Dasatinib | 5 | 3EBY_2 | 2 | 5 | 5 | Novosphingobium aromaticivorans | BETA SUBUNIT OF A PUTATIVE AROMATIC-RING-HYDROXYLATING | 3G5D_1N11A- 2ZVA_1N1513A- |
| Dasatinib | 5 | 4Q6V_2 | 2 | 4 | 5 | Salmonella enterica | PENICILLIN-BINDING PROTEIN ACTIVATOR LPOB | 3G5D_1N11A- 2ZVA_1N1513A- |
| Dasatinib | 5 | 2J7Q_5 | 2 | 5 | 5 | Murid herpesvirus 1 | UBIQUITIN | 2Y6O_1N11892A- 3QLG_1N1601AA |
| Crizotinib | 5 | 1H2S_7 | 3 | 5 | 5 | Natronomonas pharaonis | SENSORY RHODOPSIN II | 2XP2_VGH9000A- 4ANQ_VGH9000A- 2YFX_VGH9000A- |
| Crizotinib | 5 | 1KU3_1 | 2 | 5 | 5 | Thermus aquaticus | SIGMA FACTOR SIGA | 4ANQ_VGH9000A- 2YFX_VGH9000A- |
| Crizotinib | 5 | 1G8Q_1 | 2 | 5 | 5 | Homo sapiens | CD81 ANTIGEN, EXTRACELLULAR DOMAIN | 3ZBF_VGH3000A- 2XP2_VGH9000A- |
| Crizotinib | 5 | 4PND_2 | 2 | 4 | 5 | synthetic construct | CC-PENT_VARIANT | 3ZBF_VGH3000A- 2XP2_VGH9000A- |
| Crizotinib | 5 | 3TTC_5 | 2 | 2 | 5 | Escherichia coli | TRANSCRIPTIONAL REGULATORY PROTEIN | 4ANQ_VGH9000A- 2YFX_VGH9000A- |
| Crizotinib | 5 | 2RFF_4 | 2 | 4 | 5 | Sulfolobus solfataricus | PUTATIVE NUCLEOTIDYLTRANSFERASE | 3ZBF_VGH3000A- 2XP2_VGH9000A- |
| Crizotinib | 5 | 4HH5_5 | 2 | 5 | 5 | Escherichia coli | PUTATIVE TYPE VI SECRETION PROTEIN | 2XP2_VGH9000A- 4ANQ_VGH9000A- |
| Zoledronate | 4 | 4GMQ_1 | 4 | 4 | 4 | Chaetomium thermophilum | PUTATIVE RIBOSOME ASSOCIATED PROTEIN | 3N45_ZOL354F- 2F9K_ZOL9001F- 4KFA_ZOL404A- 2E91_ZOL901A- |
| Zoledronate | 4 | 3AGN_4 | 4 | 4 | 4 | Ustilago sphaerogena | RIBONUCLEASE U2 | 3N45_ZOL354F- 2F9K_ZOL9001F- 4KFA_ZOL404A- 2E91_ZOL901A- |
| Zoledronate | 4 | 2O34_3 | 3 | 4 | 4 | Desulfovibrio vulgaris | HYPOTHETICAL PROTEIN | 3N45_ZOL354F- 4KFA_ZOL404A- 2E91_ZOL901A- |
| Zoledronate | 4 | 1PZ7_5 | 3 | 4 | 4 | Gallus gallus | AGRIN | 3N45_ZOL354F- 2F9K_ZOL9001F- 4KFA_ZOL404A- |
| Zoledronate | 4 | 2XUS_2 | 3 | 4 | 4 | Homo sapiens | BREAST CANCER METASTASIS-SUPPRESSOR 1 | 2F9K_ZOL9001F- 4KFA_ZOL404A- 2E91_ZOL901A- |
| Zoledronate | 4 | 1YB3_3 | 3 | 4 | 4 | Pyrococcus furiosus | HYPOTHETICAL PROTEIN | 3N45_ZOL354F- 2F9K_ZOL9001F- 4KFA_ZOL404A- |
| Zoledronate | 4 | 4PJR_5 | 2 | 4 | 4 | unidentified | PENTATRICOPEPTIDE REPEAT PROTEIN | 2F9K_ZOL9001F- 4KFA_ZOL404A- |
| Zoledronate | 4 | 4I6R_1 | 2 | 4 | 4 | Enterobacter sp. RFL1396 | REGULATORY PROTEIN | 2F9K_ZOL9001F- 2E91_ZOL901A- |
| Zoledronate | 4 | 4KUE_5 | 2 | 4 | 4 | Clostridium butyricum | 3-HYDROXYBUTYRYL-COA DEHYDROGENASE | 2F9K_ZOL9001F- 2E91_ZOL901A- |
| Zoledronate | 4 | 3MF7_3 | 2 | 4 | 4 | coryneform bacterium | CIS-3-CHLOROACRYLIC ACID DEHALOGENASE | 2F9K_ZOL9001F- 4KFA_ZOL404A- |
| Zoledronate | 4 | 4Q8K_2 | 2 | 4 | 4 | Pseudoalteromonas sp. SM0524 | ALGINASE | 4KFA_ZOL404A- 2E91_ZOL901A- |
| Zoledronate | 4 | 2OPC_2 | 2 | 4 | 4 | Melampsora lini | AVRL567-A | 4KFA_ZOL404A- 2E91_ZOL901A- |
| Zoledronate | 4 | 3KD3_4 | 2 | 3 | 4 | Francisella tularensis | PHOSPHOSERINE PHOSPHOHYDROLASE-LIKE PROTEIN | 2F9K_ZOL9001F- 4KFA_ZOL404A- |
| Zoledronate | 4 | 1K4I_1 | 2 | 4 | 4 | Magnaporthe grisea | 3,4-DIHYDROXY-2-BUTANONE 4-PHOSPHATE SYNTHASE | 3N45_ZOL354F- 2F9K_ZOL9001F- |
| Methazolamide | 4 | 1TT8_6 | 2 | 4 | 4 | Escherichia coli | CHORISMATE-PYRUVATE LYASE | 3DAZ_MZM263A- 1BZM_MZM262A- |
| Methazolamide | 4 | 3EF8_3 | 2 | 2 | 4 | Novosphingobium aromaticivorans | PUTATIVE SCYALONE DEHYDRATASE | 3DAZ_MZM263A- 3DCS_MZM263A- |
| Methazolamide | 4 | 2WGK_1 | 2 | 3 | 4 | - | 3,6-DIKETOCAMPHANE 1,6 MONOOXYGENASE | 3DAZ_MZM263A- 3DCS_MZM263A- |
| Raloxifene | 4 | 4PND_1 | 3 | 3 | 4 | synthetic construct | CC-PENT_VARIANT | 1ERR_RAL600A- 1QKN_RAL600A- 2JFA_RAL600A- |
| Raloxifene | 4 | 3QP4_1 | 2 | 4 | 4 | Chromobacterium violaceum | CVIR TRANSCRIPTIONAL REGULATOR | 1ERR_RAL600A- 1QKN_RAL600A- |
| Raloxifene | 4 | 4OJU_3 | 2 | 4 | 4 | Pseudoflavonifractor capillosus | HYPOTHETICAL LEUCINE RICH REPEAT PROTEIN | 2QXS_RAL600A- 2JFA_RAL600A- |
| Raloxifene | 4 | 1KU3_1 | 2 | 4 | 4 | Thermus aquaticus | SIGMA FACTOR SIGA | 2QXS_RAL600A- 1QKN_RAL600A- |
| Raloxifene | 4 | 1MZ9_1 | 2 | 4 | 4 | Mus musculus | CARTILAGE OLIGOMERIC MATRIX PROTEIN | 2QXS_RAL600A- 1ERR_RAL600A- |
| Raloxifene | 4 | 3FSS_5 | 2 | 4 | 4 | Saccharomyces cerevisiae | HISTONE CHAPERONE RTT106 | 1ERR_RAL600A- 1QKN_RAL600A- |
| Raloxifene | 4 | 4TW3_1 | 2 | 4 | 4 | Prochlorococcus marinus | ALDEHYDE DECARBONYLASE | 1QKN_RAL600A- 2JFA_RAL600A- |
| Raloxifene | 4 | 1VMG_2 | 2 | 4 | 4 | Sulfolobus solfataricus | HYPOTHETICAL PROTEIN SSO3215 | 1ERR_RAL600A- 1QKN_RAL600A- |
| Raloxifene | 4 | 2EH3_1 | 2 | 4 | 4 | Aquifex aeolicus | TRANSCRIPTIONAL REGULATOR | 1QKN_RAL600A- 2JFA_RAL600A- |
| Ethoxzolamide | 4 | 3KS3_2 | 4 | 4 | 4 | Homo sapiens | CARBONIC ANHYDRASE 2 | 3MDZ_EZL264A- 3DD0_EZL301A- 3DCW_EZL301A- 3CAJ_EZL265A- |
| Ethoxzolamide | 4 | 2V9L_5 | 2 | 4 | 4 | Escherichia coli | RHAMNULOSE-1-PHOSPHATE ALDOLASE | 3MDZ_EZL264A- 3CAJ_EZL265A- |
| Ethoxzolamide | 4 | 2WGK_1 | 2 | 3 | 4 | - | 3,6-DIKETOCAMPHANE 1,6 MONOOXYGENASE | 3DD0_EZL301A- 3CAJ_EZL265A- |
| Ethoxzolamide | 4 | 2WGK_2 | 2 | 3 | 3 | - | 3,6-DIKETOCAMPHANE 1,6 MONOOXYGENASE | 3DCW_EZL301A- 3CAJ_EZL265A- |
| Ethoxzolamide | 4 | 3WKX_1 | 2 | 3 | 4 | Bifidobacterium longum | NON-REDUCING END BETA-L-ARABINOFURANOSIDASE | 3DD0_EZL301A- 3CAJ_EZL265A- |
| Mifepristone | 4 | 1H2S_6 | 4 | 4 | 4 | Natronomonas pharaonis | SENSORY RHODOPSIN II | 3H52_4864C- 4LTW_486303A- 2W8Y_4861000A- 1NHZ_486800A- |
| Mifepristone | 4 | 4UYB_4 | 4 | 4 | 4 | Homo sapiens | SEC14-LIKE PROTEIN 3 | 3H52_4864C- 4LTW_486303A- 2W8Y_4861000A- 1NHZ_486800A- |
| Mifepristone | 4 | 3FGV_1 | 3 | 3 | 4 | Ruegeria pomeroyi | UNCHARACTERIZED PROTEIN WITH FERREDOXIN-LIKE FOLD | 3H52_4864C- 2W8Y_4861000A- 1NHZ_486800A- |
| Mifepristone | 4 | 2DWK_5 | 3 | 3 | 4 | Mus musculus | PROTEIN RUFY3 | 3H52_4864C- 2W8Y_4861000A- 1NHZ_486800A- |
| Mifepristone | 4 | 3FSS_5 | 3 | 3 | 4 | Saccharomyces cerevisiae | HISTONE CHAPERONE RTT106 | 3H52_4864C- 2W8Y_4861000A- 1NHZ_486800A- |
| Mifepristone | 4 | 3K3C_4 | 3 | 3 | 4 | Mycobacterium tuberculosis | PROTEIN RV1364C/MT1410 | 3H52_4864C- 2W8Y_4861000A- 1NHZ_486800A- |
| Mifepristone | 4 | 3S0A_1 | 3 | 3 | 3 | Apis mellifera | OBP14 | 3H52_4864C- 2W8Y_4861000A- 1NHZ_486800A- |
| Mifepristone | 4 | 4PF3_2 | 3 | 3 | 3 | Homo sapiens | MINERALOCORTICOID RECEPTOR | 3H52_4864C- 2W8Y_4861000A- 1NHZ_486800A- |
| Mifepristone | 4 | 1Y6X_1 | 3 | 4 | 4 | Mycobacterium tuberculosis | PHOSPHORIBOSYL-ATP PYROPHOSPHATASE | 3H52_4864C- 4LTW_486303A- 2W8Y_4861000A- |
| Mifepristone | 4 | 3A0M_2 | 2 | 2 | 4 | - | COLLAGEN-LIKE PEPTIDE | 3H52_4864C- 2W8Y_4861000A- |
| Mifepristone | 4 | 4TW3_5 | 2 | 3 | 3 | Prochlorococcus marinus | ALDEHYDE DECARBONYLASE | 2W8Y_4861000A- 1NHZ_486800A- |
| Mifepristone | 4 | 3K3V_2 | 2 | 3 | 4 | Saccharomyces cerevisiae | PROTEIN SMY2 | 3H52_4864C- 1NHZ_486800A- |
| Mifepristone | 4 | 1VMG_2 | 2 | 3 | 4 | Sulfolobus solfataricus | HYPOTHETICAL PROTEIN SSO3215 | 3H52_4864C- 2W8Y_4861000A- |
| Mifepristone | 4 | 1YBK_2 | 2 | 3 | 3 | Staphylothermus marinus | TETRABRACHION | 2W8Y_4861000A- 1NHZ_486800A- |
| Mifepristone | 4 | 1EW0_5 | 2 | 3 | 4 | Sinorhizobium meliloti | FIXL | 3H52_4864C- 1NHZ_486800A- |
| Mifepristone | 4 | 2EH3_1 | 2 | 2 | 3 | Aquifex aeolicus | TRANSCRIPTIONAL REGULATOR | 3H52_4864C- 2W8Y_4861000A- |
| Mifepristone | 4 | 2B97_1 | 2 | 2 | 3 | Trichoderma reesei | HYDROPHOBIN II | 3H52_4864C- 2W8Y_4861000A- |
| Mifepristone | 4 | 4U9B_3 | 2 | 3 | 3 | Shewanella oneidensis | NO-BINDING HEME-DEPENDENT SENSOR PROTEIN | 3H52_4864C- 1NHZ_486800A- |
| Mifepristone | 4 | 4INW_1 | 2 | 2 | 4 | Amyelois transitella | PHEROMONE-BINDING PROTEIN 1 | 3H52_4864C- 2W8Y_4861000A- |
| Mifepristone | 4 | 1MZ9_1 | 2 | 3 | 3 | Mus musculus | CARTILAGE OLIGOMERIC MATRIX PROTEIN | 3H52_4864C- 2W8Y_4861000A- |
| Mifepristone | 4 | 4U12_2 | 2 | 3 | 4 | Helicobacter pylori | UNCHARACTERIZED PROTEIN HP0242 | 4LTW_486303A- 2W8Y_4861000A- |
| Mifepristone | 4 | 3A0M_3 | 2 | 2 | 4 | - | COLLAGEN-LIKE PEPTIDE | 3H52_4864C- 2W8Y_4861000A- |
| Mifepristone | 4 | 2XOV_2 | 2 | 4 | 4 | Escherichia coli | RHOMBOID PROTEASE GLPG | 3H52_4864C- 2W8Y_4861000A- |
| Mifepristone | 4 | 4HYL_1 | 2 | 4 | 4 | Haliangium ochraceum | STAGE II SPORULATION P. | 3H52_4864C- 1NHZ_486800A- |

^a,b,c^ Number of times this target is predicted with Z_x_ thresholds of 3, 2.5 and 2.0.

Table S4 – The 554 commonly predicted Pisces targets

| **Pisces site** | **Nb. Z_3_** | **Organism** | **Protein name** |
| --- | --- | --- | --- |
| 1H2S_7 | 90 | Natronomonas pharaonis | SENSORY RHODOPSIN II |
| 1Y6X_1 | 88 | Mycobacterium tuberculosis | PHOSPHORIBOSYL-ATP PYROPHOSPHATASE |
| 2DWK_5 | 82 | Mus musculus | PROTEIN RUFY3 |
| 3GWK_3 | 80 | Streptococcus agalactiae | PUTATIVE UNCHARACTERIZED PROTEIN SAG1039 |
| 4U9B_3 | 79 | Shewanella oneidensis | NO-BINDING HEME-DEPENDENT SENSOR PROTEIN |
| 4PND_2 | 79 | synthetic construct | CC-PENT_VARIANT |
| 4UYB_4 | 78 | Homo sapiens | SEC14-LIKE PROTEIN 3 |
| 3FSS_5 | 77 | Saccharomyces cerevisiae | HISTONE CHAPERONE RTT106 |
| 1H2S_6 | 76 | Natronomonas pharaonis | SENSORY RHODOPSIN II |
| 3S0A_1 | 72 | Apis mellifera | OBP14 |
| 1SN9_2 | 71 | - | TETRAMERIC BETA-BETA-ALPHA MINI-PROTEIN |
| 3CLA_2 | 69 | Escherichia coli | TYPE III CHLORAMPHENICOL ACETYLTRANSFERASE |
| 4TW3_5 | 68 | Prochlorococcus marinus | ALDEHYDE DECARBONYLASE |
| 3FGV_1 | 67 | Ruegeria pomeroyi | UNCHARACTERIZED PROTEIN WITH FERREDOXIN-LIKE FOLD |
| 3RFI_2 | 64 | Solanum tuberosum | ASP |
| 2XOV_2 | 63 | Escherichia coli | RHOMBOID PROTEASE GLPG |
| 1XIO_1 | 61 | Nostoc sp. PCC 7120 | ANABAENA SENSORY RHODOPSIN |
| 4PF3_2 | 60 | Homo sapiens | MINERALOCORTICOID RECEPTOR |
| 1VMG_2 | 60 | Sulfolobus solfataricus | HYPOTHETICAL PROTEIN SSO3215 |
| 1EW0_5 | 60 | Sinorhizobium meliloti | FIXL |
| 1YBK_2 | 54 | Staphylothermus marinus | TETRABRACHION |
| 3LDC_1 | 54 | Methanothermobacter thermautotrophicus | CALCIUM-GATED POTASSIUM CHANNEL MTHK |
| 4I66_2 | 52 | Haliangium ochraceum | UNCHARACTERIZED PROTEIN HOCH_4089 |
| 2P09_3 | 52 | unidentified | A NON-BIOLOGICAL ATP BINDING PROTEIN WITH TWO MUTATIONS |
| 2RFF_4 | 52 | Sulfolobus solfataricus | PUTATIVE NUCLEOTIDYLTRANSFERASE |
| 1XSV_3 | 52 | Staphylococcus aureus | HYPOTHETICAL UPF0122 PROTEIN SAV1236 |
| 3N1E_4 | 50 | Mus musculus | VACUOLAR PROTEIN SORTING-ASSOCIATED PROTEIN 54 |
| 3HWU_2 | 47 | Cupriavidus pinatubonensis | PUTATIVE DNA-BINDING PROTEIN |
| 3ECF_5 | 47 | Anabaena variabilis | NTF2-LIKE PROTEIN |
| 4Q6V_2 | 46 | Salmonella enterica | PENICILLIN-BINDING PROTEIN ACTIVATOR LPOB |
| 2J7Q_5 | 45 | Murid herpesvirus 1 | UBIQUITIN |
| 4TW3_1 | 45 | Prochlorococcus marinus | ALDEHYDE DECARBONYLASE |
| 2XEU_1 | 44 | Homo sapiens | RING FINGER PROTEIN 4 |
| 3PCV_1 | 43 | Homo sapiens | LEUKOTRIENE C4 SYNTHASE |
| 1WV3_1 | 41 | Staphylococcus aureus | SIMILAR TO DNA SEGREGATION ATPASE AND RELATED |
| 3CIM_3 | 41 | Synechocystis sp. | CARBON DIOXIDE-CONCENTRATING MECHANISM PROTEIN CCMK HOMOLOG |
| 4M5B_4 | 40 | Caldanaerobacter subterraneus | COBALAMIN BIOSYNTHESIS PROTEIN CBIM |
| 3O6P_3 | 40 | Enterococcus faecalis | PEPTIDE ABC TRANSPORTER, PEPTIDE-BINDING PROTEIN |
| 2EH3_1 | 40 | Aquifex aeolicus | TRANSCRIPTIONAL REGULATOR |
| 2XGV_4 | 40 | Microcebus murinus | PSIV CAPSID N-TERMINAL DOMAIN |
| 2EWT_2 | 39 | Streptomyces coelicolor | PUTATIVE DNA-BINDING PROTEIN |
| 4GOF_5 | 39 | Homo sapiens | SMALL GLUTAMINE-RICH TETRATRICOPEPTIDE REPEAT-CONTAINING |
| 4U12_2 | 38 | Helicobacter pylori | UNCHARACTERIZED PROTEIN HP0242 |
| 2OOA_3 | 37 | Homo sapiens | E3 UBIQUITIN-PROTEIN LIGASE CBL-B |
| 2O71_1 | 36 | Homo sapiens | DEATH DOMAIN-CONTAINING PROTEIN CRADD |
| 4PND_1 | 35 | synthetic construct | CC-PENT_VARIANT |
| 3EBY_2 | 35 | Novosphingobium aromaticivorans | BETA SUBUNIT OF A PUTATIVE AROMATIC-RING-HYDROXYLATING |
| 4PNO_1 | 35 | Escherichia coli | RNA-BINDING PROTEIN HFQ |
| 3K3C_4 | 33 | Mycobacterium tuberculosis | PROTEIN RV1364C/MT1410 |
| 1PZX_4 | 33 | Geobacillus stearothermophilus | HYPOTHETICAL PROTEIN APC36103 |
| 2B97_1 | 33 | Trichoderma reesei | HYDROPHOBIN II |
| 4A5X_4 | 31 | Homo sapiens | MIT DOMAIN-CONTAINING PROTEIN 1 |
| 3OST_1 | 31 | Saccharomyces cerevisiae | SERINE/THREONINE-PROTEIN KINASE KCC4 |
| 1TUK_2 | 30 | Triticum aestivum | NONSPECIFIC LIPID-TRANSFER PROTEIN 2G |
| 4HH5_5 | 30 | Escherichia coli | PUTATIVE TYPE VI SECRETION PROTEIN |
| 3K3V_2 | 30 | Saccharomyces cerevisiae | PROTEIN SMY2 |
| 3DKR_2 | 29 | Lactobacillus rhamnosus | ESTERASE D |
| 3WMI_3 | 29 | Equus caballus | EIAV GP45 WILD TYPE |
| 1V2Z_4 | 28 | Thermosynechococcus elongatus | CIRCADIAN CLOCK PROTEIN KAIA HOMOLOG |
| 1YBK_1 | 28 | Staphylothermus marinus | TETRABRACHION |
| 2IAY_1 | 28 | Lactobacillus plantarum | HYPOTHETICAL PROTEIN |
| 3O48_3 | 28 | Saccharomyces cerevisiae | MITOCHONDRIA FISSION 1 PROTEIN |
| 4LPQ_1 | 28 | Xylanimonas cellulosilytica | ERFK/YBIS/YCFS/YNHG FAMILY PROTEIN |
| 2FEF_2 | 28 | Pseudomonas aeruginosa | HYPOTHETICAL PROTEIN PA2201 |
| 4IGV_1 | 27 | Actinidia deliciosa | KIROLA |
| 1P9H_5 | 26 | Yersinia enterocolitica | INVASIN |
| 3F0P_1 | 25 | Escherichia coli | ALKYLMERCURY LYASE |
| 4LLE_4 | 25 | Pseudomonas aeruginosa | PROBABLE TWO-COMPONENT SENSOR |
| 3G0O_2 | 25 | Salmonella enterica | 3-HYDROXYISOBUTYRATE DEHYDROGENASE |
| 4PZJ_2 | 24 | Eggerthella lenta | TRANSCRIPTIONAL REGULATOR, LYSR FAMILY |
| 3LK7_3 | 24 | Streptococcus agalactiae | UDP-N-ACETYLMURAMOYLALANINE--D-GLUTAMATE LIGASE |
| 4I2L_2 | 24 | Human immunodeficiency virus 1 | GP41 |
| 1Y9L_1 | 23 | Shigella flexneri | LIPOPROTEIN MXIM |
| 4LPI_1 | 22 | Physeter catodon | MYOGLOBIN |
| 4HYL_1 | 22 | Haliangium ochraceum | STAGE II SPORULATION PROTEIN |
| 3A1H_6 | 22 | - | COLLAGEN-LIKE PEPTIDE |
| 2O38_2 | 21 | Rhodopseudomonas palustris | HYPOTHETICAL PROTEIN |
| 3A0M_2 | 21 | - | COLLAGEN-LIKE PEPTIDE |
| 2ZFZ_4 | 20 | Mycobacterium tuberculosis | ARGININE REPRESSOR |
| 1M0K_1 | 20 | Halobacterium salinarum | BACTERIORHODOPSIN |
| 2GDM_1 | 20 | Lupinus luteus | LEGHEMOGLOBIN (OXY) |
| 3JR7_5 | 20 | [Ruminococcus] gnavus | UNCHARACTERIZED EGV FAMILY PROTEIN COG1307 |
| 2P6V_4 | 20 | Homo sapiens | TRANSCRIPTION INITIATION FACTOR TFIID SUBUNIT 4 |
| 1G8Q_1 | 19 | Homo sapiens | CD81 ANTIGEN, EXTRACELLULAR DOMAIN |
| 3AH9_2 | 19 | Saimiriine herpesvirus 2 | COLLAGEN-LIKE PEPTIDE |
| 3W7Y_1 | 18 | Homo sapiens | INSULIN |
| 2RIJ_2 | 18 | Campylobacter jejuni | PUTATIVE 2,3,4,5-TETRAHYDROPYRIDINE-2-CARBOXYLATE N- |
| 1ZVA_4 | 18 | Severe acute respiratory syndrome-related coronavirus | E2 GLYCOPROTEIN |
| 1MZ9_1 | 18 | Mus musculus | CARTILAGE OLIGOMERIC MATRIX PROTEIN |
| 4PVK_1 | 18 | Phleum pratense | POLLEN ALLERGEN PHL P 4.0202 |
| 3JSR_1 | 18 | Nostoc sp. PCC 7120 | ALL0216 PROTEIN |
| 1Z0P_1 | 18 | Streptococcus pyogenes | HYPOTHETICAL PROTEIN SPY1572 |
| 2O1K_1 | 18 | Rotavirus A | NON-STRUCTURAL GLYCOPROTEIN NSP4 |
| 4KV2_1 | 18 | Mycobacterium tuberculosis | ESX-1 SECRETION SYSTEM PROTEIN ECCD1 |
| 4A20_2 | 18 | Saccharomyces cerevisiae | UBIQUITIN-LIKE PROTEIN MDY2 |
| 4DJG_1 | 17 | Arabidopsis thaliana | PLECTIN-RELATED PROTEIN |
| 4UOT_1 | 17 | synthetic construct | DESIGNED HELICAL BUNDLE 5H2L |
| 3QM9_1 | 17 | Thunnus atlanticus | MYOGLOBIN |
| 3H51_2 | 17 | Xanthomonas campestris | PUTATIVE CALCIUM/CALMODULIN DEPENDENT PROTEIN KINASE II |
| 3KS3_2 | 17 | Homo sapiens | CARBONIC ANHYDRASE 2 |
| 3B2Y_2 | 17 | Shewanella denitrificans | METALLOPEPTIDASE CONTAINING CO-CATALYTIC METALLOACTIVE |
| 4INW_1 | 17 | Amyelois transitella | PHEROMONE-BINDING PROTEIN 1 |
| 3ZY7_1 | 17 | Mus musculus | AP-1 COMPLEX SUBUNIT GAMMA-1 |
| 2J97_1 | 17 | Human coronavirus 229E | REPLICASE POLYPROTEIN 1AB |
| 4NOH_3 | 17 | Bacillus anthracis | LIPOPROTEIN, PUTATIVE |
| 3EF8_3 | 16 | Novosphingobium aromaticivorans | PUTATIVE SCYALONE DEHYDRATASE |
| 3KG4_4 | 16 | [Mannheimia] succiniciproducens | UNCHARACTERIZED PROTEIN |
| 3JTZ_3 | 16 | Yersinia pestis | INTEGRASE |
| 2CX7_2 | 16 | Thermus thermophilus | STEROL CARRIER PROTEIN 2 |
| 4PN8_3 | 16 | synthetic construct | CC-PENT |
| 1TT8_6 | 16 | Escherichia coli | CHORISMATE-PYRUVATE LYASE |
| 3QP4_1 | 16 | Chromobacterium violaceum | CVIR TRANSCRIPTIONAL REGULATOR |
| 4OFK_4 | 16 | Caenorhabditis elegans | PROTEIN SYG-2 |
| 3KFF_5 | 16 | Mus musculus | MAJOR URINARY PROTEIN 4 |
| 1VI4_1 | 16 | Vibrio cholerae | REGULATOR OF RIBONUCLEASE ACIVITY A PROTEIN 1 |
| 1WN2_1 | 15 | Pyrococcus horikoshii | PEPTIDYL-TRNA HYDROLASE |
| 4DT5_4 | 15 | Rhagium inquisitor | ANTIFREEZE PROTEIN |
| 1PZ7_5 | 15 | Gallus gallus | AGRIN |
| 2YFU_3 | 15 | Ruminiclostridium thermocellum | CARBOHYDRATE BINDING FAMILY 6 |
| 2Z1C_3 | 15 | Thermococcus kodakarensis | HYDROGENASE EXPRESSION/FORMATION PROTEIN HYPC |
| 1UIX_1 | 15 | Bos taurus | RHO-ASSOCIATED KINASE |
| 1T6U_1 | 15 | Streptomyces coelicolor | SUPEROXIDE DISMUTASE [NI] |
| 3FH1_3 | 14 | Mesorhizobium loti | UNCHARACTERIZED NTF2-LIKE PROTEIN |
| 1TQG_3 | 14 | Thermotoga maritima | CHEMOTAXIS PROTEIN CHEA |
| 2HX5_1 | 14 | Prochlorococcus marinus | HYPOTHETICAL PROTEIN |
| 3WKX_1 | 14 | Bifidobacterium longum | NON-REDUCING END BETA-L-ARABINOFURANOSIDASE |
| 1RH4_1 | 14 | synthetic construct | RIGHT-HANDED COILED COIL TETRAMER |
| 4GIP_2 | 14 | Parainfluenza virus 5 | FUSION GLYCOPROTEIN F2 |
| 2QIB_2 | 14 | Streptomyces coelicolor | TETR-FAMILY TRANSCRIPTIONAL REGULATOR |
| 3A0M_3 | 14 | - | COLLAGEN-LIKE PEPTIDE |
| 3ADM_2 | 14 | - | COLLAGEN-LIKE PEPTIDE |
| 2WJN_1 | 14 | Blastochloris viridis | PHOTOSYNTHETIC REACTION CENTER CYTOCHROME C SUBUNIT |
| 4B21_3 | 14 | Schizosaccharomyces pombe-synthetic construct | PROBABLE DNA-3-METHYLADENINE GLYCOSYLASE 2 |
| 4HS2_2 | 14 | Homo sapiens | SPECKLE-TYPE POZ PROTEIN |
| 4J8C_1 | 14 | Rattus norvegicus | HSC70-INTERACTING PROTEIN |
| 2Q12_1 | 14 | Homo sapiens | DCC-INTERACTING PROTEIN 13 ALPHA |
| 3HV2_1 | 14 | Pseudomonas protegens | RESPONSE REGULATOR/HD DOMAIN PROTEIN |
| 3AH9_3 | 13 | Saimiriine herpesvirus 2 | COLLAGEN-LIKE PEPTIDE |
| 1PZ4_1 | 13 | Aedes aegypti | STEROL CARRIER PROTEIN 2 |
| 1H8P_3 | 13 | Bos taurus | SEMINAL PLASMA PROTEIN PDC-109 |
| 1Q8B_2 | 13 | Bacillus subtilis | PROTEIN YJCS |
| 4OJU_3 | 13 | Pseudoflavonifractor capillosus | HYPOTHETICAL LEUCINE RICH REPEAT PROTEIN |
| 1GMX_1 | 13 | Escherichia coli | THIOSULFATE SULFURTRANSFERASE GLPE |
| 4GMQ_1 | 13 | Chaetomium thermophilum | PUTATIVE RIBOSOME ASSOCIATED PROTEIN |
| 3ROB_4 | 13 | Planctopirus limnophila | UNCHARACTERIZED CONSERVED PROTEIN |
| 2MLT_1 | 13 | Apis mellifera | MELITTIN |
| 1FMJ_2 | 13 | Spodoptera frugiperda | RETINOL DEHYDRATASE |
| 2P97_2 | 13 | Anabaena variabilis | HYPOTHETICAL PROTEIN |
| 4LWU_1 | 13 | Xenopus laevis | E3 UBIQUITIN-PROTEIN LIGASE MDM2 |
| 1KU3_1 | 13 | Thermus aquaticus | SIGMA FACTOR SIGA |
| 4X86_3 | 13 | Homo sapiens | UBIQUITIN-LIKE PROTEIN 4A |
| 3NY3_1 | 13 | Homo sapiens | E3 UBIQUITIN-PROTEIN LIGASE UBR2 |
| 1R5L_1 | 13 | Homo sapiens | PROTEIN (ALPHA-TOCOPHEROL TRANSFER PROTEIN) |
| 1C5E_2 | 13 | Enterobacteria phage lambda | HEAD DECORATION PROTEIN |
| 3O48_2 | 13 | Saccharomyces cerevisiae | MITOCHONDRIA FISSION 1 PROTEIN |
| 2G3W_4 | 13 | Xanthomonas citri | HYPOTHETICAL PROTEIN XAC2396 |
| 3LHC_2 | 12 | Nostoc ellipsosporum | CYANOVIRIN-N |
| 2RH3_2 | 12 | Agrobacterium tumefaciens | PROTEIN VIRC2 |
| 1W0N_1 | 12 | Paenibacillus polymyxa | ENDO-1,4-BETA-XYLANASE D |
| 2P09_1 | 12 | unidentified | A NON-BIOLOGICAL ATP BINDING PROTEIN WITH TWO MUTATIONS |
| 2X7Q_2 | 12 | Candida albicans | POSSIBLE THIAMINE BIOSYNTHESIS ENZYME |
| 2HRA_3 | 12 | Saccharomyces cerevisiae | GLUTAMYL-TRNA SYNTHETASE, CYTOPLASMIC |
| 4H8M_1 | 12 | synthetic construct | CC-HEX-H24-A5/7C |
| 1EZ3_3 | 12 | Rattus norvegicus | SYNTAXIN-1A |
| 2ZS0_3 | 12 | Oligobrachia mashikoi | EXTRACELLULAR GIANT HEMOGLOBIN MAJOR GLOBIN SUBUNIT A1 |
| 3NPH_5 | 12 | Synechocystis sp. PCC 6803 | PHYCOBILISOME 32.1 KDA LINKER POLYPEPTIDE, PHYCOCYANIN- |
| 4FTF_1 | 12 | Vibrio cholerae | ALTERNATE SECRETIN PATHWAY SUBUNIT S (VC395_1821, VC1703) |
| 3FTJ_3 | 12 | Aggregatibacter actinomycetemcomitans | MACROLIDE EXPORT ATP-BINDING/PERMEASE PROTEIN |
| 2NWF_5 | 12 | Rhodobacter sphaeroides | UBIQUINOL-CYTOCHROME C REDUCTASE IRON-SULFUR SUBUNIT |
| 2WP7_1 | 12 | Mus musculus | PPPDE PEPTIDASE DOMAIN-CONTAINING PROTEIN 2 |
| 3W0E_2 | 12 | Aspergillus fumigatus | ELASTASE INHIBITOR AFUEI |
| 4JPR_2 | 11 | Rous sarcoma virus | ASLV FUSION TM |
| 1F94_1 | 11 | Bungarus candidus | BUCANDIN |
| 2C2Q_2 | 11 | Deinococcus radiodurans | G/U MISMATCH-SPECIFIC DNA GLYCOSYLASE |
| 1Z6M_2 | 11 | Enterococcus faecalis | CONSERVED HYPOTHETICAL PROTEIN |
| 2P7I_2 | 11 | Pectobacterium atrosepticum | HYPOTHETICAL PROTEIN |
| 3MVU_2 | 11 | Ruegeria sp. TM1040 | TENA FAMILY TRANSCRIPTIONAL REGULATOR |
| 3Q8G_1 | 11 | Saccharomyces cerevisiae | CRAL-TRIO DOMAIN-CONTAINING PROTEIN YKL091C |
| 4Q2W_3 | 11 | Streptococcus pneumoniae | PUTATIVE ENDO-BETA-N-ACETYLGLUCOSAMINIDASE |
| 4JOQ_3 | 11 | Rhodobacter sphaeroides | ABC RIBOSE TRANSPORTER, PERIPLASMIC SOLUTE-BINDING PROTEIN |
| 1RZH_1 | 11 | Rhodobacter sphaeroides | REACTION CENTER PROTEIN L CHAIN |
| 3QW9_7 | 11 | Rattus norvegicus | TRANSFORMING GROWTH FACTOR BETA RECEPTOR TYPE 3 |
| 1X1K_2 | 11 | Saimiriine herpesvirus 2 | HOST-GUEST PEPTIDE (PRO-PRO-GLY)4-(PRO-ALLOHYP- |
| 2OU6_4 | 11 | Deinococcus radiodurans | HYPOTHETICAL PROTEIN |
| 3B0T_1 | 11 | Homo sapiens | VITAMIN D3 RECEPTOR |
| 4GQM_2 | 11 | Chlamydia trachomatis | CT009 |
| 4NSV_1 | 11 | Lysobacter enzymogenes | LYSYL ENDOPEPTIDASE |
| 2RH2_1 | 11 | Escherichia coli | DIHYDROFOLATE REDUCTASE TYPE 2 |
| 1Q4V_2 | 10 | Homo sapiens | INSULIN |
| 3CI9_1 | 10 | Homo sapiens | HEAT SHOCK FACTOR-BINDING PROTEIN 1 |
| 3TQ2_1 | 10 | synthetic construct | KE1 |
| 3U5V_5 | 10 | Homo sapiens#Mus musculus | PROTEIN MAX, TRANSCRIPTION FACTOR E2-ALPHA CHIMERA |
| 1K4I_1 | 10 | Magnaporthe grisea | 3,4-DIHYDROXY-2-BUTANONE 4-PHOSPHATE SYNTHASE |
| 4KQI_8 | 10 | Salmonella enterica | NICOTINATE-NUCLEOTIDE--DIMETHYLBENZIMIDAZOLE |
| 4QB0_4 | 10 | Zaire ebolavirus | NUCLEOPROTEIN |
| 2R2Z_1 | 10 | Enterococcus faecalis | HEMOLYSIN |
| 3D4E_2 | 10 | Streptococcus mutans | PUTATIVE BETA-LACTAMASE INHIBITOR PROTEIN |
| 1FV1_7 | 10 | Homo sapiens | MAJOR HISTOCOMPATIBILITY COMPLEX ALPHA CHAIN |
| 1VPK_9 | 10 | Thermotoga maritima | DNA POLYMERASE III, BETA SUBUNIT |
| 1NKD_1 | 10 | Escherichia coli | ROP |
| 2WGK_1 | 10 | - | 3,6-DIKETOCAMPHANE 1,6 MONOOXYGENASE |
| 3F2E_3 | 10 | Rudivirus | SIRV COAT PROTEIN |
| 3BHO_3 | 10 | Homo sapiens | CLEAVAGE AND POLYADENYLATION SPECIFICITY FACTOR |
| 3OXP_3 | 10 | Yersinia pestis | PHOSPHOTRANSFERASE ENZYME II, A COMPONENT |
| 3KBG_2 | 10 | Thermoplasma acidophilum | 30S RIBOSOMAL PROTEIN S4E |
| 3B33_2 | 9 | Vibrio parahaemolyticus | SENSOR PROTEIN |
| 3U23_1 | 9 | Homo sapiens | CD2-ASSOCIATED PROTEIN |
| 2OXO_2 | 9 | unidentified phage | INTEGRASE |
| 1WNA_1 | 9 | Thermus thermophilus | THE HYPOTHETICAL PROTEIN (TT1805) |
| 4PJR_5 | 9 | unidentified | PENTATRICOPEPTIDE REPEAT PROTEIN |
| 1KU3_2 | 9 | Thermus aquaticus | SIGMA FACTOR SIGA |
| 3EN0_4 | 9 | Synechocystis sp. PCC 6803 | CYANOPHYCINASE |
| 3BLN_2 | 9 | Bacillus cereus | ACETYLTRANSFERASE GNAT FAMILY |
| 2A2M_5 | 9 | Bacteroides thetaiotaomicron | HYPOTHETICAL PROTEIN BT3146 |
| 4A4J_1 | 9 | Synechocystis sp. PCC 6803 | COPPER-TRANSPORTING ATPASE PACS |
| 4DB5_2 | 9 | Oryctolagus cuniculus | TUMOR NECROSIS FACTOR LIGAND SUPERFAMILY MEMBER 18 |
| 1J8R_1 | 9 | Escherichia coli | PYELONEPHRITIC ADHESIN |
| 4N4J_1 | 9 | Candidatus Kuenenia stuttgartiensis | HYDROXYLAMINE OXIDOREDUCTASE |
| 1WY3_1 | 9 | Gallus gallus | VILLIN |
| 4F98_2 | 9 | Pseudomonas aeruginosa | HYPOTHETICAL PROTEIN |
| 4QHQ_3 | 9 | Burkholderia cenocepacia | LIPOPROTEIN |
| 2BT9_2 | 9 | Ralstonia solanacearum | LECTIN |
| 4H7R_4 | 9 | synthetic construct | CC-HEX-II |
| 4RN7_5 | 9 | Peptoclostridium difficile | N-ACETYLMURAMOYL-L-ALANINE AMIDASE |
| 3F7E_6 | 9 | Mycobacterium smegmatis | PYRIDOXAMINE 5'-PHOSPHATE OXIDASE-RELATED, FMN- |
| 2CYJ_3 | 9 | Pyrococcus horikoshii | HYPOTHETICAL PROTEIN PH1505 |
| 3MCX_4 | 9 | Bacteroides thetaiotaomicron | SUSD SUPERFAMILY PROTEIN |
| 3PD7_3 | 9 | Homo sapiens | DNA TOPOISOMERASE 2-BINDING PROTEIN 1 |
| 1AHO_1 | 9 | Androctonus australis | TOXIN II |
| 1I71_2 | 9 | Homo sapiens | APOLIPOPROTEIN(A) |
| 1NWZ_2 | 9 | Halorhodospira halophila | PHOTOACTIVE YELLOW PROTEIN |
| 4WHI_3 | 9 | Mycobacterium tuberculosis | BETA-LACTAMASE |
| 1I8A_1 | 9 | Thermotoga maritima | ENDO-1,4-BETA-XYLANASE A |
| 1ZMM_4 | 8 | Homo sapiens | NEUTROPHIL DEFENSIN 4 |
| 4I2L_1 | 8 | Human immunodeficiency virus 1 | GP41 |
| 1I5G_1 | 8 | Crithidia fasciculata | TRYPAREDOXIN II |
| 3Q8J_1 | 8 | Asteropus | ASTEROPSIN A |
| 4RN7_4 | 8 | Peptoclostridium difficile | N-ACETYLMURAMOYL-L-ALANINE AMIDASE |
| 3QRL_4 | 8 | Saccharomyces cerevisiae | TRANSCRIPTION INITIATION FACTOR TFIID SUBUNIT 14 |
| 1XAK_1 | 8 | Severe acute respiratory syndrome-related coronavirus | SARS ORF7A ACCESSORY PROTEIN |
| 1COJ_1 | 8 | Aquifex pyrophilus | PROTEIN (SUPEROXIDE DISMUTASE) |
| 1WHZ_1 | 8 | Thermus thermophilus | HYPOTHETICAL PROTEIN |
| 2G9W_1 | 8 | Mycobacterium tuberculosis | CONSERVED HYPOTHETICAL PROTEIN |
| 1LO7_3 | 8 | Pseudomonas sp. CBS3 | 4-HYDROXYBENZOYL-COA THIOESTERASE |
| 2OB3_2 | 8 | Brevundimonas diminuta | PARATHION HYDROLASE |
| 2XCI_3 | 8 | Aquifex aeolicus | 3-DEOXY-D-MANNO-2-OCTULOSONIC ACID TRANSFERASE |
| 1TXL_1 | 8 | Escherichia coli | METAL-BINDING PROTEIN YODA |
| 4I17_3 | 8 | Bacteroides fragilis | HYPOTHETICAL PROTEIN |
| 4LUK_1 | 8 | Pyrococcus furiosus | GLUCOSE-6-PHOSPHATE ISOMERASE |
| 3WMV_1 | 8 | Mytilus galloprovincialis | LECTIN |
| 1NQ7_1 | 8 | Rattus norvegicus | NUCLEAR RECEPTOR ROR-BETA |
| 3ZJA_5 | 8 | Streptomyces lividans | SL3965 |
| 2X9O_3 | 8 | Synechococcus sp. WH 8020 | 15,16-DIHYDROBILIVERDIN-FERREDOXIN OXIDOREDUCTASE |
| 3NJD_4 | 8 | Mycobacterium smegmatis | ENOYL-COA HYDRATASE |
| 3GT5_2 | 8 | Xylella fastidiosa | N-ACETYLGLUCOSAMINE 2-EPIMERASE |
| 2YZQ_2 | 8 | Pyrococcus horikoshii | PUTATIVE UNCHARACTERIZED PROTEIN PH1780 |
| 4BT7_2 | 8 | Brevibacillus brevis | ALPHA-ACETOLACTATE DECARBOXYLASE |
| 4N3X_2 | 8 | Homo sapiens | RAB5 GDP/GTP EXCHANGE FACTOR |
| 2XDH_1 | 8 | Archaeoglobus fulgidus | COHESIN |
| 4OXX_3 | 8 | Citrobacter braakii | CINDOXIN |
| 3WIT_5 | 8 | Escherichia coli | PUTATIVE VGR PROTEIN |
| 2FI1_4 | 8 | Streptococcus pneumoniae | HYDROLASE, HALOACID DEHALOGENASE-LIKE FAMILY |
| 2O34_3 | 8 | Desulfovibrio vulgaris | HYPOTHETICAL PROTEIN |
| 2VOF_4 | 8 | Mus musculus | BCL-2-RELATED PROTEIN A1 |
| 2XUS_2 | 8 | Homo sapiens | BREAST CANCER METASTASIS-SUPPRESSOR 1 |
| 2X5Y_1 | 8 | Homo sapiens | ZINC FINGER CCCH-TYPE ANTIVIRAL PROTEIN 1 |
| 4BOQ_1 | 8 | Homo sapiens | UBIQUITIN THIOESTERASE OTU1 |
| 3TYS_2 | 8 | Enterococcus faecalis | PREDICTED TRANSCRIPTIONAL REGULATOR |
| 2GGC_1 | 8 | Escherichia coli | METHIONINE AMINOPEPTIDASE |
| 3G46_1 | 8 | Scapharca inaequivalvis | GLOBIN-1 |
| 2MHR_2 | 8 | Themiste hennahi | MYOHEMERYTHRIN |
| 2GZV_3 | 8 | Homo sapiens | PRKCA-BINDING PROTEIN |
| 4DZN_1 | 8 | - | COILED-COIL PEPTIDE CC-PIL |
| 4PN8_5 | 8 | synthetic construct | CC-PENT |
| 1JNI_4 | 8 | Haemophilus influenzae | DIHEME CYTOCHROME C NAPB |
| 4MDW_1 | 8 | Bacillus subtilis | UNCHARACTERIZED PROTEIN YDHK |
| 4J73_4 | 7 | Saccharomyces cerevisiae | COATOMER SUBUNIT BETA' |
| 2WJR_5 | 7 | Escherichia coli | PROBABLE N-ACETYLNEURAMINIC ACID OUTER MEMBRANE CHANNEL |
| 2JG0_1 | 7 | Escherichia coli | PERIPLASMIC TREHALASE |
| 3I5W_2 | 7 | Homo sapiens | DEFENSIN-5 |
| 1Q8D_2 | 7 | Rattus norvegicus | GDNF FAMILY RECEPTOR ALPHA 1 |
| 3CZ1_2 | 7 | Apis mellifera | PHEROMONE-BINDING PROTEIN ASP1 |
| 1YB3_3 | 7 | Pyrococcus furiosus | HYPOTHETICAL PROTEIN |
| 3POD_1 | 7 | - | MBL COLLAGEN-LIKE PEPTIDE |
| 2NV0_4 | 7 | Bacillus subtilis | GLUTAMINE AMIDOTRANSFERASE SUBUNIT PDXT |
| 4Q53_5 | 7 | Bacteroides uniformis | UNCHARACTERIZED PROTEIN |
| 3LDC_2 | 7 | Methanothermobacter thermautotrophicus | CALCIUM-GATED POTASSIUM CHANNEL MTHK |
| 4MNO_5 | 7 | Pyrococcus abyssi | TRANSLATION INITIATION FACTOR 1A |
| 4GGC_2 | 7 | Homo sapiens | CELL DIVISION CYCLE PROTEIN 20 HOMOLOG |
| 4O7Q_2 | 7 | Homo sapiens | INTERFERON-INDUCIBLE PROTEIN AIM2 |
| 3TZ1_2 | 7 | Chlamys nipponensis | TROPONIN C |
| 3LM3_5 | 7 | Parabacteroides distasonis | UNCHARACTERIZED PROTEIN |
| 1VYI_3 | 7 | Rabies virus | RNA POLYMERASE ALPHA SUBUNIT |
| 1RVK_1 | 7 | Agrobacterium fabrum | ISOMERASE/LACTONIZING ENZYME |
| 1NKD_2 | 7 | Escherichia coli | ROP |
| 1V9Y_2 | 7 | Escherichia coli | HEME PAS SENSOR PROTEIN |
| 4PNL_5 | 7 | Homo sapiens | TANKYRASE-2 |
| 4JXR_2 | 7 | Sinorhizobium meliloti | ACETYLTRANSFERASE |
| 3E1R_5 | 7 | Homo sapiens | CENTROSOMAL PROTEIN OF 55 KDA |
| 4TXR_15 | 7 | Homo sapiens | CHARGED MULTIVESICULAR BODY PROTEIN 1B |
| 2VO8_2 | 7 | Clostridium perfringens | EXO-ALPHA-SIALIDASE |
| 3M1X_4 | 7 | Entamoeba histolytica | PUTATIVE ENDORIBONUCLEASE L-PSP |
| 4LKU_3 | 7 | Escherichia coli | LARGE-CONDUCTANCE MECHANOSENSITIVE CHANNEL |
| 2OLT_3 | 7 | Shewanella oneidensis | HYPOTHETICAL PROTEIN |
| 1WHZ_4 | 7 | Thermus thermophilus | HYPOTHETICAL PROTEIN |
| 1TUH_1 | 7 | uncultured bacterium | HYPOTHETICAL PROTEIN EGC068 |
| 2WW6_2 | 7 | Enterobacteria phage T4 sensu lato | FIBRITIN |
| 3DFG_5 | 7 | Xanthomonas campestris | REGULATORY PROTEIN RECX |
| 4GYX_4 | 7 | Homo sapiens | TYPE III COLLAGEN FRAGMENT IN A HOST PEPTIDE STABILIZED BY |
| 3HKM_5 | 7 | Oryza sativa | OS03G0854200 PROTEIN |
| 1ZMI_1 | 7 | Homo sapiens | NEUTROPHIL DEFENSIN 2 |
| 2HO2_5 | 7 | Homo sapiens | AMYLOID BETA A4 PROTEIN-BINDING FAMILY B MEMBER 1 |
| 3AGN_4 | 7 | Ustilago sphaerogena | RIBONUCLEASE U2 |
| 3LWC_3 | 7 | Rhizobium leguminosarum | UNCHARACTERIZED PROTEIN |
| 3BS5_2 | 7 | Homo sapiens | PROTEIN AVEUGLE |
| 2MLT_2 | 7 | Apis mellifera | MELITTIN |
| 1SAU_2 | 7 | Archaeoglobus fulgidus | SULFITE REDUCTASE, DESULFOVIRIDIN-TYPE SUBUNIT GAMMA |
| 2IQY_1 | 7 | Rattus norvegicus | PHOSPHATIDYLETHANOLAMINE-BINDING PROTEIN 1 |
| 4ADI_2 | 7 | Rubella virus | E1 ENVELOPE GLYCOPROTEIN |
| 3HTN_2 | 7 | Bacteroides thetaiotaomicron | PUTATIVE DNA BINDING PROTEIN |
| 2IVY_7 | 7 | Sulfolobus solfataricus | HYPOTHETICAL PROTEIN SSO1404 |
| 3NQN_5 | 7 | Deinococcus radiodurans | UNCHARACTERIZED PROTEIN |
| 4GNR_2 | 7 | Streptococcus pneumoniae | ABC TRANSPORTER SUBSTRATE-BINDING PROTEIN-BRANCHED CHAIN |
| 3LEQ_4 | 7 | Streptomyces avermitilis | UNCHARACTERIZED PROTEIN CVNB5 |
| 3LUU_1 | 7 | Acidithiobacillus ferrooxidans | UNCHARACTERIZED PROTEIN |
| 1M0D_5 | 7 | Enterobacteria phage T7 | ENDODEOXYRIBONUCLEASE I |
| 2BTI_4 | 7 | Yersinia enterocolitica | CARBON STORAGE REGULATOR HOMOLOG |
| 2O6N_2 | 7 | - | RH4B DESIGNED PEPTIDE |
| 2HLY_2 | 7 | Agrobacterium fabrum | HYPOTHETICAL PROTEIN ATU2299 |
| 3ZBD_1 | 7 | Alphacoronavirus 1 | NON-STRUCTURAL PROTEIN 1 |
| 2Z3Q_1 | 7 | Homo sapiens | INTERLEUKIN-15 |
| 4RQA_1 | 7 | Staphylococcus aureus | HYPOXANTHINE PHOSPHORIBOSYLTRANSFERASE |
| 3TY1_1 | 7 | Klebsiella pneumoniae | HYPOTHETICAL ALDOSE 1-EPIMERASE |
| 3AG3_2 | 6 | Bos taurus | CYTOCHROME C OXIDASE SUBUNIT 1 |
| 2V52_10 | 6 | Oryctolagus cuniculus-Mus musculus | ACTIN, ALPHA SKELETAL MUSCLE |
| 1FT5_1 | 6 | Nitrosomonas europaea | CYTOCHROME C554 |
| 4L3U_1 | 6 | Acinetobacter baumannii | UNCHARACTERIZED PROTEIN |
| 3OIO_1 | 6 | Chromobacterium violaceum | TRANSCRIPTIONAL REGULATOR (ARAC-TYPE DNA-BINDING DOMAIN- |
| 2ZVY_5 | 6 | Salmonella enterica | CHEMOTAXIS PROTEIN MOTB |
| 3FYR_2 | 6 | Bacillus subtilis | SPORULATION INHIBITOR SDA |
| 2OPW_1 | 6 | Homo sapiens | PHYHD1 PROTEIN |
| 2Y71_3 | 6 | Mycobacterium tuberculosis | 3-DEHYDROQUINATE DEHYDRATASE |
| 1JO0_1 | 6 | Haemophilus influenzae | HYPOTHETICAL PROTEIN HI1333 |
| 2OZL_2 | 6 | Homo sapiens | PYRUVATE DEHYDROGENASE E1 COMPONENT ALPHA |
| 2VPT_1 | 6 | Ruminiclostridium thermocellum | LIPOLYTIC ENZYME |
| 1SEN_4 | 6 | Homo sapiens | THIOREDOXIN-LIKE PROTEIN P19 |
| 3CBN_1 | 6 | Methanothermobacter thermautotrophicus | CONSERVED PROTEIN MTH639 |
| 2WN3_5 | 6 | Dictyostelium discoideum | DISCOIDIN-1 SUBUNIT A |
| 3WMV_4 | 6 | Mytilus galloprovincialis | LECTIN |
| 3TWE_2 | 6 | - | ALPHA4H |
| 4U7I_2 | 6 | Homo sapiens | SPARTIN |
| 4Q8K_2 | 6 | Pseudoalteromonas sp. SM0524 | ALGINASE |
| 2WGK_2 | 6 | - | 3,6-DIKETOCAMPHANE 1,6 MONOOXYGENASE |
| 1G12_2 | 6 | Grifola frondosa | PEPTIDYL-LYS METALLOENDOPEPTIDASE |
| 2NS9_2 | 6 | Aeropyrum pernix | HYPOTHETICAL PROTEIN APE2225 |
| 4CV7_1 | 6 | Rhodococcus equi | VIRULENCE ASSOCIATED PROTEIN VAPB |
| 2I5I_3 | 6 | Enterococcus faecalis | UPF0249 PROTEIN EF_3048 |
| 3B47_5 | 6 | Geobacter sulfurreducens | METHYL-ACCEPTING CHEMOTAXIS PROTEIN |
| 2QSB_2 | 6 | Thermoplasma acidophilum | UPF0147 PROTEIN TA0600 |
| 1UKK_6 | 6 | Thermus thermophilus | OSMOTICALLY INDUCIBLE PROTEIN C |
| 3SK9_5 | 6 | Thermus thermophilus | PUTATIVE UNCHARACTERIZED PROTEIN TTHB187 |
| 3UI4_5 | 6 | Homo sapiens | PEPTIDYL-PROLYL CIS-TRANS ISOMERASE NIMA-INTERACTING 4 |
| 1T07_2 | 6 | Pseudomonas aeruginosa | HYPOTHETICAL UPF0269 PROTEIN PA5148 |
| 3F40_3 | 6 | Cytophaga hutchinsonii | UNCHARACTERIZED NTF2-LIKE PROTEIN |
| 1BXY_6 | 6 | Thermus thermophilus | PROTEIN (RIBOSOMAL PROTEIN L30) |
| 1X0T_4 | 6 | Pyrococcus horikoshii | RIBONUCLEASE P PROTEIN COMPONENT 4 |
| 3FBL_1 | 6 | Acidianus filamentous virus 1 | PUTATIVE UNCHARACTERIZED PROTEIN |
| 4CG1_1 | 6 | Thermobifida fusca | CUTINASE |
| 4H7R_3 | 6 | synthetic construct | CC-HEX-II |
| 1L6P_1 | 6 | Escherichia coli | THIOL:DISULFIDE INTERCHANGE PROTEIN DSBD |
| 3OFG_1 | 6 | Caenorhabditis elegans | BOCA/MESD CHAPERONE FOR YWTD BETA-PROPELLER-EGF PROTEIN 1 |
| 3DSM_2 | 6 | Bacteroides uniformis | UNCHARACTERIZED PROTEIN BACUNI_02894 |
| 1PZW_1 | 6 | Drosophila melanogaster | TRANSCRIPTION FACTOR GRAUZONE |
| 4IAU_5 | 6 | Geodia cydonium | BETA-GAMMA-CRYSTALLIN |
| 3PE9_4 | 6 | Ruminiclostridium thermocellum | FIBRONECTIN(III)-LIKE MODULE |
| 3S8S_3 | 6 | Homo sapiens | HISTONE-LYSINE N-METHYLTRANSFERASE SETD1A |
| 3L15_2 | 6 | Homo sapiens | TRANSCRIPTIONAL ENHANCER FACTOR TEF-4 |
| 3H2Z_3 | 6 | Shigella flexneri | MANNITOL-1-PHOSPHATE 5-DEHYDROGENASE |
| 2FB6_3 | 6 | Bacteroides thetaiotaomicron | CONSERVED HYPOTHETICAL PROTEIN |
| 4H79_1 | 6 | Thermobifida fusca | CRISPR-ASSOCIATED PROTEIN, CSE2 FAMILY |
| 3HSH_2 | 6 | Homo sapiens | COLLAGEN ALPHA-1(XVIII) CHAIN |
| 2E3H_1 | 6 | Homo sapiens | RESTIN |
| 2VB1_4 | 6 | Gallus gallus | LYSOZYME C |
| 3FYR_1 | 6 | Bacillus subtilis | SPORULATION INHIBITOR SDA |
| 4DQJ_5 | 6 | Pseudomonas phage phi6 | MEMBRANE PROTEIN PHI6 P5 |
| 4I6R_1 | 6 | Enterobacter sp. RFL1396 | REGULATORY PROTEIN |
| 1DXG_1 | 6 | Desulfovibrio gigas | DESULFOREDOXIN |
| 1KS9_2 | 6 | Escherichia coli | 2-DEHYDROPANTOATE 2-REDUCTASE |
| 2TGI_3 | 6 | Homo sapiens | TRANSFORMING GROWTH FACTOR ,BETA 2 |
| 1MXR_1 | 6 | Escherichia coli | RIBONUCLEOTIDE REDUCTASE R2 |
| 1I2T_2 | 6 | Homo sapiens | HYD PROTEIN |
| 2POR_5 | 6 | Rhodobacter capsulatus | PORIN |
| 4KU0_2 | 6 | Enterobacteria phage T4 sensu lato | TAIL-ASSOCIATED LYSOZYME |
| 3OV5_1 | 6 | Xanthomonas citri | UNCHARACTERIZED PROTEIN |
| 3HG7_2 | 6 | Aeromonas salmonicida | D-ISOMER SPECIFIC 2-HYDROXYACID DEHYDROGENASE FAMILY |
| 3NYM_4 | 6 | Neisseria meningitidis | UNCHARACTERIZED PROTEIN |
| 3NUF_1 | 6 | Lactobacillus casei | PRD-CONTAINING TRANSCRIPTION REGULATOR |
| 1TFE_7 | 6 | Thermus thermophilus | ELONGATION FACTOR TS |
| 1CGD_2 | 6 | - | COLLAGEN-LIKE PEPTIDE |
| 3ZT9_3 | 6 | Moorella thermoacetica | SERINE PHOSPHATASE |
| 4P5N_5 | 6 | Cryptococcus neoformans | HYPOTHETICAL PROTEIN CNAG_02591 |
| 3SP7_8 | 6 | Homo sapiens | BCL-2-LIKE PROTEIN 1 |
| 3GIW_1 | 6 | Streptomyces avermitilis | PROTEIN OF UNKNOWN FUNCTION DUF574 |
| 2WOY_5 | 6 | Streptococcus gordonii | AGGLUTININ RECEPTOR |
| 1Z96_2 | 6 | Schizosaccharomyces pombe | UBA-DOMAIN PROTEIN MUD1 |
| 4GLQ_1 | 6 | Thermosynechococcus elongatus | METHYL-ACCEPTING CHEMOTAXIS PROTEIN |
| 1R9W_2 | 6 | Alphapapillomavirus 7 | REPLICATION PROTEIN E1 |
| 4HGU_2 | 6 | Galleria mellonella | SILK PROTEASE INHIBITOR 2 |
| 1ZMI_4 | 6 | Homo sapiens | NEUTROPHIL DEFENSIN 2 |
| 2Q4M_2 | 6 | Arabidopsis thaliana | PROTEIN AT5G01750 |
| 2O30_4 | 6 | Encephalitozoon cuniculi | NUCLEAR MOVEMENT PROTEIN |
| 4HSQ_5 | 6 | Corynebacterium diphtheriae | PUTATIVE FIMBRIAL SUBUNIT |
| 2GB4_5 | 6 | Mus musculus | THIOPURINE S-METHYLTRANSFERASE |
| 4NE3_2 | 6 | Homo sapiens | CENTROMERE PROTEIN S |
| 4H5I_5 | 6 | Saccharomyces cerevisiae | GUANINE NUCLEOTIDE-EXCHANGE FACTOR SEC12 |
| 3BS3_2 | 6 | Bacteroides fragilis | PUTATIVE DNA-BINDING PROTEIN |
| 3F6O_4 | 6 | Rhodococcus jostii | PROBABLE TRANSCRIPTIONAL REGULATOR, ARSR FAMILY |
| 1Y0N_2 | 6 | Pseudomonas aeruginosa | HYPOTHETICAL UPF0270 PROTEIN PA3463 |
| 1F8E_1 | 6 | Influenza A virus | NEURAMINIDASE |
| 3ZHI_2 | 6 | Lactococcus phage TP901-1 | CI |
| 3LBW_1 | 6 | Influenza A virus | M2 PROTEIN |
| 3OV9_5 | 6 | Rift Valley fever virus | NUCLEOPROTEIN |
| 1OK0_2 | 6 | Streptomyces tendae | ALPHA-AMYLASE INHIBITOR HOE-467A |
| 4NSM_1 | 6 | Streptococcus pyogenes | COLLAGEN-LIKE PROTEIN SCLB |
| 1K6K_6 | 6 | Escherichia coli | ATP-DEPENDENT CLP PROTEASE ATP-BINDING SUBUNIT |
| 3NIR_2 | 6 | Crambe hispanica | CRAMBIN |
| 1HXR_4 | 6 | Rattus norvegicus | GUANINE NUCLEOTIDE EXCHANGE FACTOR MSS4 |
| 4LBF_3 | 6 | Homo sapiens | NEUTROPHIL DEFENSIN 1 |
| 2ZQ5_1 | 6 | Mycobacterium tuberculosis | PUTATIVE UNCHARACTERIZED PROTEIN |
| 3NZ3_2 | 6 | Streptococcus pneumoniae | PUTATIVE UNCHARACTERIZED PROTEIN |
| 4OWI_1 | 6 | synthetic construct | P53LZ2 |
| 4GF3_5 | 6 | Yersinia pestis | PUTATIVE YOPH TARGETING PROTEIN |
| 4C6E_2 | 6 | Homo sapiens | DIHYDROOROTASE |
| 4JIF_4 | 5 | Homo sapiens | INTEGRIN BETA-1-BINDING PROTEIN 1 |
| 1C4Q_3 | 5 | Escherichia coli | PROTEIN (SHIGA-LIKE TOXIN I SUBUNIT B) |
| 2E1V_4 | 5 | Chrysanthemum x morifolium | ACYL TRANSFERASE |
| 3Q1N_1 | 5 | Lactobacillus casei | GALACTOSE MUTAROTASE RELATED ENZYME |
| 1T6L_3 | 5 | Human herpesvirus 5 | DNA POLYMERASE PROCESSIVITY FACTOR |
| 3IUF_1 | 5 | Homo sapiens | ZINC FINGER PROTEIN UBI-D4 |
| 1Q7L_1 | 5 | Homo sapiens | AMINOACYLASE-1 |
| 3H93_2 | 5 | Pseudomonas aeruginosa | THIOL:DISULFIDE INTERCHANGE PROTEIN DSBA |
| 4MXT_2 | 5 | Bacteroides uniformis | UNCHARACTERIZED PROTEIN |
| 3S6F_3 | 5 | Deinococcus radiodurans | HYPOTHETICAL ACETYLTRANSFERASE |
| 4JWO_4 | 5 | Planctopirus limnophila | PHOSPHATE BINDING PROTEIN |
| 2BBE_7 | 5 | Shewanella oneidensis | HYPOTHETICAL PROTEIN SO0527 |
| 2CMP_1 | 5 | Bacillus phage SF6 | TERMINASE SMALL SUBUNIT |
| 1RYQ_3 | 5 | Pyrococcus furiosus | DNA-DIRECTED RNA POLYMERASE, SUBUNIT E'' |
| 1ZMA_1 | 5 | Streptococcus pneumoniae | BACTEROCIN TRANSPORT ACCESSORY PROTEIN |
| 4QA8_1 | 5 | Mycobacterium bovis | PUTATIVE LIPOPROTEIN LPRF |
| 2WFU_2 | 5 | Drosophila melanogaster | PROBABLE INSULIN-LIKE PEPTIDE 5 A CHAIN |
| 4KT6_4 | 5 | Streptococcus pyogenes | NICOTINE ADENINE DINUCLEOTIDE GLYCOHYDROLASE |
| 2DSX_2 | 5 | Desulfovibrio gigas | RUBREDOXIN |
| 4IAX_4 | 5 | Homo sapiens | NEUTROPHIL GELATINASE-ASSOCIATED LIPOCALIN |
| 4EW7_1 | 5 | Salmonella enterica | CONJUGATIVE TRANSFER: REGULATION |
| 3ONH_1 | 5 | Saccharomyces cerevisiae | UBIQUITIN-ACTIVATING ENZYME E1-LIKE |
| 2RCI_2 | 5 | Bacillus thuringiensis | TYPE-2BA CYTOLYTIC DELTA-ENDOTOXIN |
| 3LAG_1 | 5 | Rhodopseudomonas palustris | UNCHARACTERIZED PROTEIN RPA4178 |
| 3BS5_3 | 5 | Drosophila melanogaster-Homo sapiens | PROTEIN AVEUGLE |
| 3HFT_2 | 5 | Bordetella bronchiseptica | WBMS, POLYSACCHARIDE DEACETYLASE INVOLVED IN O-ANTIGEN |
| 2AG4_5 | 5 | Homo sapiens | GANGLIOSIDE GM2 ACTIVATOR |
| 2X5P_1 | 5 | Streptococcus pyogenes | FIBRONECTIN BINDING PROTEIN |
| 3A02_4 | 5 | Drosophila melanogaster | HOMEOBOX PROTEIN ARISTALESS |
| 1CGD_3 | 5 | - | COLLAGEN-LIKE PEPTIDE |
| 3IG9_3 | 5 | Enterobacteria phage RB69 | SOC SMALL OUTER CAPSID PROTEIN |
| 3BP3_3 | 5 | Escherichia coli | GLUCOSE-SPECIFIC PHOSPHOTRANSFERASE ENZYME IIB |
| 4OY6_2 | 5 | Streptomyces coelicolor | PUTATIVE SECRETED CELLULOSE-BINDING PROTEIN |
| 2O2X_5 | 5 | Mesorhizobium loti | HYPOTHETICAL PROTEIN |
| 3MQZ_2 | 5 | Leptospirillum rubarum | UNCHARACTERIZED CONSERVED PROTEIN DUF1054 |
| 3LJW_2 | 5 | Homo sapiens | PROTEIN POLYBROMO-1 |
| 2HHZ_3 | 5 | Streptococcus suis | PYRIDOXAMINE 5'-PHOSPHATE OXIDASE-RELATED |
| 4PT1_3 | 5 | Locusta migratoria | ODORANT-BINDING PROTEIN 1D |
| 2O6L_5 | 5 | Homo sapiens | UDP-GLUCURONOSYLTRANSFERASE 2B7 |
| 4NOA_5 | 5 | Pseudomonas aeruginosa | TYPE 4 FIMBRIAL BIOGENESIS PROTEIN PILE |
| 4EZG_4 | 5 | Listeria monocytogenes | PUTATIVE UNCHARACTERIZED PROTEIN |
| 4H0C_4 | 5 | Dyadobacter fermentans | PHOSPHOLIPASE/CARBOXYLESTERASE |
| 4N1V_1 | 5 | Spiroplasma melliferum | DNA-BINDING PROTEIN HU-BETA |
| 3IUF_2 | 5 | Homo sapiens | ZINC FINGER PROTEIN UBI-D4 |
| 1BKV_2 | 5 | - | T3-785 |
| 3S6E_2 | 5 | Mus musculus | RNA-BINDING PROTEIN 39 |
| 4QY7_3 | 5 | Bacillus subtilis | UNCHARACTERIZED PROTEIN YOBA |
| 3NOJ_3 | 5 | Pseudomonas putida | 4-CARBOXY-4-HYDROXY-2-OXOADIPATE ALDOLASE/OXALOACETATE |
| 4MGP_3 | 5 | Xenopus laevis | MAGAININ 2 DERIVATIVE |
| 3U5V_2 | 5 | Homo sapiens#Mus musculus | PROTEIN MAX, TRANSCRIPTION FACTOR E2-ALPHA CHIMERA |
| 1T3Y_5 | 5 | Homo sapiens | COACTOSIN-LIKE PROTEIN |
| 4EMO_5 | 5 | Homo sapiens | SHARPIN |
| 2GZQ_2 | 5 | Plasmodium vivax | PHOSPHATIDYLETHANOLAMINE-BINDING PROTEIN |
| 2YXB_5 | 5 | Aeropyrum pernix | COENZYME B12-DEPENDENT MUTASE |
| 3E4H_1 | 5 | Viola arvensis | VARV PEPTIDE F |
| 2RHF_3 | 5 | Deinococcus radiodurans | DNA HELICASE RECQ |
| 4EVQ_4 | 5 | Rhodopseudomonas palustris | PUTATIVE ABC TRANSPORTER SUBUNIT, SUBSTRATE-BINDING |
| 3ZUI_3 | 5 | Ornithodoros moubata | COMPLEMENT INHIBITOR |
| 2HZC_3 | 5 | Homo sapiens | SPLICING FACTOR U2AF 65 KDA SUBUNIT |
| 3U01_4 | 5 | Rana pipiens | PROTEIN P-30 |
| 2EFJ_5 | 5 | Coffea canephora | 3,7-DIMETHYLXANTHINE METHYLTRANSFERASE |
| 2XOM_2 | 5 | Thermotoga maritima | ARABINOGALACTAN ENDO-1,4-BETA-GALACTOSIDASE |
| 1X6I_3 | 5 | Escherichia coli | HYPOTHETICAL PROTEIN YGFY |
| 3IQT_3 | 5 | Escherichia coli | SIGNAL TRANSDUCTION HISTIDINE-PROTEIN KINASE BARA |
| 3CJM_4 | 5 | Enterococcus faecalis | PUTATIVE BETA-LACTAMASE |
| 3NOJ_5 | 5 | Pseudomonas putida | 4-CARBOXY-4-HYDROXY-2-OXOADIPATE ALDOLASE/OXALOACETATE |
| 2QSK_3 | 5 | Scytonema varium | SCYTOVIRIN |
| 1MWP_5 | 5 | Homo sapiens | AMYLOID A4 PROTEIN |
| 1TOA_2 | 5 | Treponema pallidum | PROTEIN (PERIPLASMIC BINDING PROTEIN TROA) |
| 4OI3_1 | 5 | Streptomyces coelicolor | NICKEL RESPONSIVE PROTEIN |
| 4EA9_4 | 5 | Caulobacter vibrioides | PEROSAMINE N-ACETYLTRANSFERASE |
| 2OPC_2 | 5 | Melampsora lini | AVRL567-A |
| 3ITQ_8 | 5 | Bacillus anthracis | PROLYL 4-HYDROXYLASE, ALPHA SUBUNIT DOMAIN PROTEIN |
| 3P46_4 | 5 | - | SYNTHETIC COLLAGEN PEPTIDE |
| 1YJ7_2 | 5 | Escherichia coli | ESCJ |
| 2QZQ_2 | 5 | Danio rerio | AXIN INTERACTOR, DORSALIZATION ASSOCIATED |
| 4QKD_4 | 5 | Homo sapiens | ALPHA-KETOGLUTARATE-DEPENDENT DIOXYGENASE ALKB HOMOLOG 7, |
| 2OH3_4 | 5 | Magnetospirillum magnetotacticum | COG1633: UNCHARACTERIZED CONSERVED PROTEIN |
| 4AFM_2 | 5 | [Eubacterium] cellulosolvens | ENDOGLUCANASE CEL5A |
| 4P6B_6 | 5 | metagenome | EST-Y29 |
| 2HSB_1 | 5 | Archaeoglobus fulgidus | HYPOTHETICAL UPF0332 PROTEIN AF0298 |
| 3HMS_3 | 5 | Homo sapiens | HEPATOCYTE GROWTH FACTOR |
| 1IXH_2 | 5 | Escherichia coli | PHOSPHATE-BINDING PROTEIN |
| 4R3Q_2 | 5 | Mus musculus | SYNAPTONEMAL COMPLEX CENTRAL ELEMENT PROTEIN 3 |
| 1T6O_3 | 5 | Measles virus | PHOSPHOPROTEIN |
| 3K62_4 | 5 | Caenorhabditis elegans | FEM-3 MRNA-BINDING FACTOR 2 |
| 2H1C_4 | 5 | Neisseria gonorrhoeae | TRAFFICKING PROTEIN B |
| 2YWI_2 | 5 | Geobacillus kaustophilus | HYPOTHETICAL CONSERVED PROTEIN |
| 3GA8_4 | 5 | Escherichia coli | HTH-TYPE TRANSCRIPTIONAL REGULATOR MQSA (YGIT/B3021) |
| 3WX4_3 | 5 | Enterobacteria phage T4 sensu lato | ANTI-RESTRICTION ENDONUCLEASE |
| 3VN0_2 | 5 | Mus musculus | VOLTAGE-GATED HYDROGEN CHANNEL 1 |
| 4KQC_2 | 5 | Brachyspira murdochii | PERIPLASMIC BINDING PROTEIN/LACI TRANSCRIPTIONAL REGULATOR |
| 1TU9_2 | 5 | Pseudomonas aeruginosa | HYPOTHETICAL PROTEIN PA3967 |
| 4OM8_5 | 5 | Mesorhizobium loti | 3-HYDROXYBUTYRYL-COA DEHYDROGENASE |
| 3FK8_2 | 5 | Xylella fastidiosa | DISULPHIDE ISOMERASE |
| 2PQ7_1 | 5 | uncultured Thermotogales bacterium | PREDICTED HD SUPERFAMILY HYDROLASE |
| 4ATM_4 | 5 | Homo sapiens | AMPHIPHYSIN |
| 2BN3_3 | 5 | Bos taurus | INSULIN |
| 3W06_2 | 5 | Arabidopsis thaliana | HYDROLASE, ALPHA/BETA FOLD FAMILY PROTEIN |
| 3P46_2 | 5 | - | SYNTHETIC COLLAGEN PEPTIDE |
| 1TT8_7 | 5 | Escherichia coli | CHORISMATE-PYRUVATE LYASE |
| 4AL0_8 | 5 | Homo sapiens | PROSTAGLANDIN E SYNTHASE |
| 3CIT_9 | 5 | Pseudomonas syringae group genomosp. 3 | SENSOR HISTIDINE KINASE |
| 4OEB_2 | 5 | Pleurotus ostreatus | PLEUROTOLYSIN A |
| 2HA8_4 | 5 | Homo sapiens | TAR (HIV-1) RNA LOOP BINDING PROTEIN |
| 3OYV_2 | 5 | Bacteroides ovatus | IMELYSIN |
| 2HIN_2 | 5 | Enterobacteria phage N15 | REPRESSOR PROTEIN |
| 2FG1_2 | 5 | Bacteroides thetaiotaomicron | CONSERVED HYPOTHETICAL PROTEIN BT1257 |
| 4LE3_9 | 5 | Podospora anserina | BETA-GLUCANASE |
| 4DRI_1 | 5 | Homo sapiens | PEPTIDYL-PROLYL CIS-TRANS ISOMERASE FKBP5 |
| 3LQB_2 | 5 | Danio rerio | LOC792177 PROTEIN |
| 1WY3_3 | 5 | Gallus gallus | VILLIN |
| 3B64_2 | 5 | Leishmania major | MACROPHAGE MIGRATION INHIBITORY FACTOR-LIKE |
| 2ZS0_2 | 5 | Oligobrachia mashikoi | EXTRACELLULAR GIANT HEMOGLOBIN MAJOR GLOBIN SUBUNIT A1 |
| 1U5P_1 | 5 | Gallus gallus | SPECTRIN ALPHA CHAIN, BRAIN |
| 2CC6_5 | 5 | Halobacterium salinarum | VNG1446H |
| 3U3L_5 | 5 | Tabanus yao | TABLYSIN 15 |
| 1ALY_2 | 5 | Homo sapiens | CD40 LIGAND |
| 4ATG_4 | 5 | Antonospora locustae | TAF6 |
| 3T7Z_3 | 5 | Methanocaldococcus jannaschii | NUCLEOLAR PROTEIN NOP 56/58 |
| 3CA7_3 | 5 | Drosophila melanogaster | PROTEIN SPITZ |
